# Exploring 2-Sulfonylpyrimidine Warheads as Acrylamide Surrogates for Targeted Covalent Inhibition: A BTK Story

**DOI:** 10.1021/acs.jmedchem.3c01927

**Published:** 2024-08-09

**Authors:** Ruxandra Moraru, Beatriz Valle-Argos, Annabel Minton, Lara Buermann, Suyin Pan, Thomas E. Wales, Raji E. Joseph, Amy H. Andreotti, Jonathan C. Strefford, Graham Packham, Matthias G. J. Baud

**Affiliations:** † School of Chemistry and Institute for Life Sciences, University of Southampton, Southampton SO17 1BJ, U.K.; ‡ Cancer Sciences, Faculty of Medicine, University of Southampton, Southampton SO16 6YD, U.K.; § Department of Chemistry and Chemical Biology, Northeastern University, Boston, Massachusetts 02115, United States; ∥ Roy J. Carver Department of Biochemistry, Biophysics and Molecular Biology, Iowa State University, Ames, Iowa 50011, United States

## Abstract

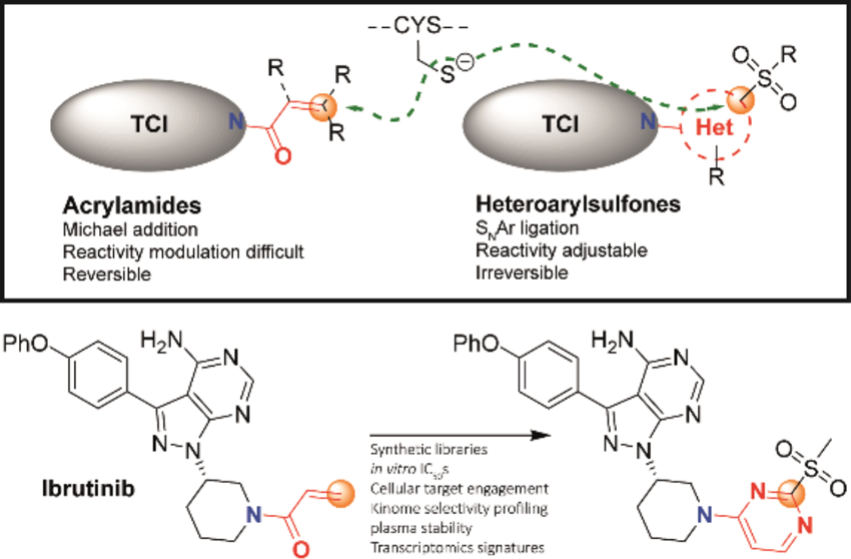

Targeted covalent inhibitors (TCIs) directing cysteine have historically relied on a narrow set of electrophilic “warheads”. While Michael acceptors remain at the forefront of TCI design strategies, they show variable stability and selectivity under physiological conditions. Here, we show that the 2-sulfonylpyrimidine motif is an effective replacement for the acrylamide warhead of Ibrutinib, for the inhibition of Bruton’s tyrosine kinase. In a few iterations, we discovered new derivatives, which inhibit BTK both *in vitro* and *in cellulo* at low nanomolar concentrations, on par with Ibrutinib. Several derivatives also displayed good plasma stability and reduced off-target binding *in vitro* across 135 tyrosine kinases. This proof-of-concept study on a well-studied kinase/TCI system highlights the 2-sulfonylpyrimidine group as a useful acrylamide replacement. In the future, it will be interesting to investigate its wider potential for developing TCIs with improved pharmacologies and selectivity profiles across structurally related protein families.

## Introduction

**Figure 1:**
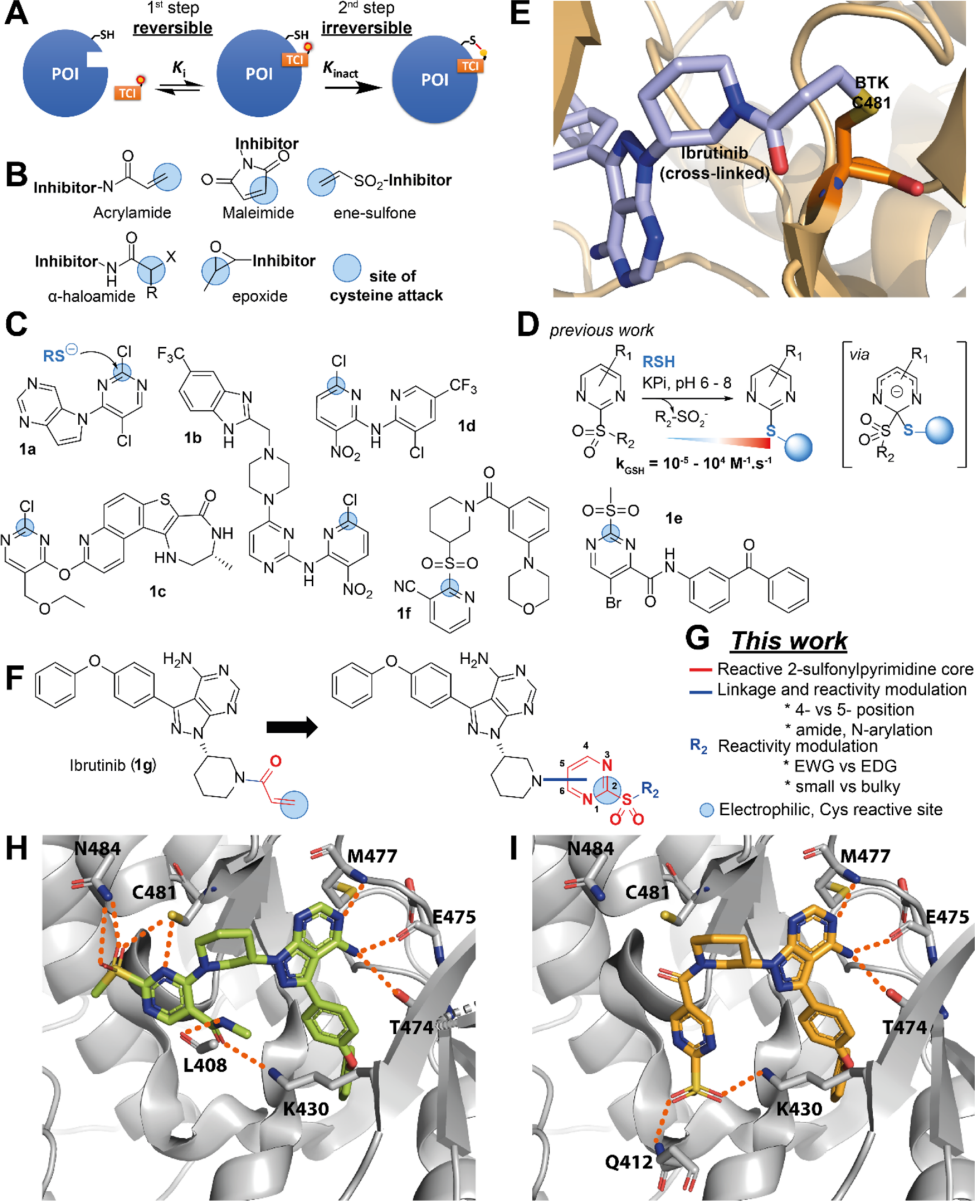
(A) General inhibitory mode of action of TCIs against the protein of interest (POI): Reversible noncovalent complex formation (step 1) followed by the formation of the irreversible covalent complex where the red lines indicate covalent bonds (step 2). E/I: enzyme/inhibitor; (B) representative examples of the most common chemical warheads used in the development of TCIs targeting protein cysteine; (C) recent examples of TCIs reacting with cysteine via an SNAr mechanism; (D) our previous work on characterizing the *in vitro* reactivity of 2-SPs with thiols using NMR, UV, MS, and XRD; (E) crystal structure of Ibrutinib (**1g**) covalently bound to BTK (PDB 5P9J). The cross-link between Ibrutinib (**1g**) and BTK C481 is highlighted; (F) structure of Ibrutinib (**1g**); (G) this work: The design of new drug analogues using 2-SP as warheads linked via amide bonds and *N*-arylation (*vide infra*); and docking poses of representative 2-SP derivatives of Ibrutinib (**1g**) with functionalization at the 4-position (H) and 5-position (I). Recurring hydrogen bonding interactions are shown as dashed orange lines. The example molecules presented in each panel are described later in the manuscript.

TCIs are protein ligands containing a reactive chemical “warhead” that is able to form a covalent bond with the protein target and inhibit its biological activity as a result.^1^ The first step involves reversible binding of the ligand, governed by *K*_i_/*K*_d_. In the second step, an electrophilic moiety from the ligand reacts with a neighboring amino acid side chain to create a covalent, irreversible linkage. The rate of covalent bond formation is characterized by *K*_inact_ and depends on several factors, including the inherent reactivities of both the electrophile and nucleophile, in addition to their relative positioning, which is to a large extent, influenced by the noncovalent preorganization of the system (Figure 1A). Unlike classical bioconjugation agents, which have high chemoselectivity but often lack regio-specificity, TCIs induce site-specific covalent protein modification. This is mainly due to the proximity-accelerated reaction facilitated by prior reversible binding, but also because by design they tend to target amino acid side chains with a low degree of conservation among the proteome. Hence, the potency and selectivity of TCIs depend on both *K*_i_ and *K*_inact_, both of which require careful consideration during TCI design and optimization. As a consequence, the irreversible nature of the inhibition mechanism makes IC_50_ measurements strongly condition-dependent (e.g., incubation time) and variable, limiting their use for characterizing the potency and selectivity of TCIs. The indicator *K*_inact_/*K*_i_, while experimentally more laborious to determine, is extensively employed and provides quantitative information on whether changes in inhibitory activity result from changes in *K*_i_ or *K*_inact_, or both. The importance of dissecting both binding thermodynamics and kinetics for TCIs have been extensively discussed.^2^

Targeted covalent inhibition of prominent disease-associated proteins is a rapidly growing area of drug discovery, presenting opportunities to enhance the potency and/or selectivity of small molecule inhibitors by allowing the covalent, irreversible association of the reactive warhead to specific sites of proteins.^3^ To date, most efforts have been directed to the covalent targeting of cysteine, mainly owing to its superior nucleophilicity and relatively low abundance at surface-exposed binding hotspots. Hence, most reported TCI warheads contain an electrophilic moiety, with α,β-unsaturated carbonyl and α-chloro carbonyl motifs being highly represented. Diverse cysteine reactive warheads have been reported, with acrylamides and related Michael acceptors being employed extensively due to their moderate reactivity (Figure 1A,B).

Tuning the inherent reactivity of the warhead is of prime importance.^4^ It should be reactive enough to allow the covalent modification of the target. At the same time, this reactivity must be moderated to minimize unspecific covalent modification of other less reactive nucleophilic sites at the protein surface and across the wider proteome, and inactivation through hydrolysis and metabolism. A number of cellular thiols, notably glutathione (GSH), are present in high cellular concentrations (up to several mM), and are well-known to “quench” a wide range of electrophilic species. Intrinsically highly reactive electrophiles, such as many maleimide derivatives for example, can show variable chemoselectivity,^5−7^ in addition to cleavage in the physiological environment via retro-Michael reaction,^8−10^ thiol exchange,^8,9,11−13^ hydrolysis^12,14^ or aminolysis,^15^ and can lead to variable efficacy and toxicity due to the formation of dynamic heterogeneous mixtures of conjugates *in vivo*.^16^ Additionally, despite advances in computational methods for *de novo* reactivity estimation, the high sensitivity of some warheads (e.g., acrylamides) to both steric and electronic factors can make it challenging to predict and adjust their reactivity, along with their *in vitro* structure–reactivity relationship and *in vivo* bioactivity profiles.^17,18^

An increasing number of TCIs containing activated aromatic and heteroaromatic electrophiles have been reported in recent years. These inhibitors react via a nucleophilic aromatic substitution (S_N_Ar) mechanism with one or several functional cysteine side chains, leading to the *S*-arylated protein product. Importantly, the S_N_Ar process remains metal-free, and the *S*-aryl covalent motif is generally markedly more stable in aqueous buffers compared to conventional warheads, circumventing stability issues of highly reactive electrophiles (notably maleimides, *vide supra*) and expanding the scope of biological applications. An increasing number of TCIs with moderated S_N_Ar reactivity have been reported in recent years, with notable examples including 2-chloropyridine and 2-chloropyrimidine-based inhibitors **1a**–**d** of MSK1,^19^ S6K2,^20^ MK2,^21,22^ and FGFR4^23^ kinases (Figure 1C), important targets in diverse cancers and autoimmune disorders, respectively. The covalent MK2 inhibitor CC-99677 (**1c**) in particular has progressed to human clinical trials for the treatment of Ankylosing Spondylitis, although it was discontinued in Phase 2 in 2023.^24^

Heteroaryl sulfones have emerged as useful reagents for the metal-free arylation of cysteine.^25^ They also react via an S_N_Ar mechanism (Figure 1D),^25^ leading to the elimination of the sulfinate leaving group. These reagents show preferential selectivity for thiols over other amino acids. Also unlike maleimides, they do not react with other oxidized sulfur species, including sulfenic acids (−SOH) and *S*-nitrosothiol (-SNO), hence presenting key advantages for chemoselective targeting *in vivo*.^26,27^ Recently, cysteine arylation with diverse 2-sulfonylpyridines, 2-sulfonylpyrimidines, 2-sulfonylbenzothiazoles, and other heterocyclic Kocieński-like reagents generally led to highly stable conjugates compared to activated Michael acceptors.^3,9,12^ Heteroaryl sulfones also display diverse reaction rates toward cysteine, modulated by the nature and electrophilicity of the heterocyclic system,^28,29^ and react under very mild conditions.^30^ Recent notable examples (Figure 1C) of heteroaryl sulfone-based TCIs include 2-sulfonylpyrimidine **1e** and 2-sulfonylpyridine **1f**-based inhibitors of *S. aureus* Sortase A^31^ and adenosine deaminase, respectively.^32^

We recently reported a comprehensive structure–reactivity relationship of libraries of 2-sulfonylpyrimidine (2-SP)-based bioorthogonal reagents for protein arylation.^30^ We showed that strategic functionalization of the 2-SP scaffold with electron withdrawing/donating groups (EWG/EDG) allows modulation of the electrophilic reactivity of 2-SP reagents toward cysteine by over 9 orders of magnitude *in vitro* (Figure 1D). Fastest reacting 2-SPs could selectively and quantitatively arylate model peptides and full proteins in a few seconds at low concentration and neutral pH, without the need of a transition metal catalyst. Last but not least, 2-SP derivatives are markedly more soluble and stable in aqueous buffers compared to conventional warheads.

We set out to investigate the potential of 2-SPs as warheads for TCI development, especially as a potential replacement for the acrylamide group. We hypothesized that the tunable reactivity of this scaffold (Figure 1D) combined with its favorable physicochemical properties and superior robustness of the resulting *S*-arylated linkage may offer additional layers of control on the potency of 2-SP functionalized TCIs. At the same time, the directional trajectory of the cysteine nucleophile imposed by the S_N_Ar process (Meisenheimer complex formation) may influence their selectivity profile and reduce off-target reactivity.

We selected the Bruton Tyrosine Kinase (BTK) as a particularly relevant test case to assess the suitability of 2-SP warheads in TCI design. BTK plays a key role in signal transduction downstream of various cell surface receptors on B cells, including the B-cell receptor (BCR) which is a key driver of several subtypes of human B-cell lymphoma and leukemia.^33,34^ Consistent with a critical role for BTK in BCR signal transduction in B cells, BTK inhibitors have proved particularly effective therapies for B-cell malignancies, including chronic lymphocytic leukemia (CLL) and some forms of lymphoma, such as mantle cell, splenic marginal zone, and primary central nervous system lymphomas.^35^ The first-in-class covalent BTK inhibitor Ibrutinib (**1g**, Figure 1E,F) has been FDA-approved since 2013 to treat diverse malignancies, notably B-cell malignancies.^36,37^ It contains an acrylamide group that reacts covalently with C^481^ at the rim of the BTK active site. However, Ibrutinib **1g** has a relatively large number of targets in addition to BTK and whereas effects on some of these targets (e.g., ITK) may contribute to its therapeutic activity, others such as EGFR appear to contribute to toxicity and other deleterious side effects in treated patients.^38^ Historically, much effort has been devoted to moderating the reactivity of well-established electrophiles of BTK inhibitors covalently reacting e.g., via nucleophilic substitution or conjugate addition, comparatively less has been reported on the use of arylation (S_*N*_Ar) chemistry for BTK inhibition.

Here, we present the design, chemical synthesis, and biological assessment of such 2-SP functionalized analogues of Ibrutinib (**1g**). We identified several derivatives displaying highly potent BTK inhibition in cellular assays, and improved selectivity profiles in kinome inhibition studies relative to Ibrutinib (**1g**). Beyond new BTK inhibitors, we are highlighting useful TCI design principles that should find broader applicability in the medicinal chemistry community focusing on TCI development.

## Results and Discussion

### Molecular Design

Our design strategy for the incorporation of 2-SP warheads in place of the acrylamide of Ibrutinib (**1g**) focused on two main aspects. First, we focused on the type of chemical linkage to connect the 2-SP warhead to the piperidine nitrogen of the Ibrutinib core (**2**, *vide infra*). We hypothesized that varying the linkage type between the 2-SP warhead and the Ibrutinib piperidine ring could allow for adjusting the potency and specificity of the resulting TCIs by (i) tuning their intrinsic electrophilic reactivity; and (ii) influencing the conformational flexibility and length between the Ibrutinib core and the warhead, hence relative spatial positioning of the warhead with respect to C481 (Figure 1G). This, together with the well-defined trajectory required for nucleophilic addition to form the Jackson–Meisenheimer complex intermediate, may provide an additional source of target specificity. For this investigation, we focused on *N*-arylation and *N*-acylation of the Ibrutinib piperidine nitrogen, since i/the corresponding amido and *N*-aryl motifs remain uncharged and conformationally biased, recapitulating several key features of the acrylamide, and ii/a wide variety of pyrimidine building blocks are commercially available and/or synthetically tractable, hence amenable to such chemistries. Second, we also explored additional substitution of the remaining aromatic positions and the exocyclic chain with EWG/EDG and/or small/bulky groups to influence reactivity and build an informative SAR.

We performed docking studies (Schrodinger) using the crystal structures of BTK in complex with Ibrutinib (**1g**) (pdb 5P9J, covalent) and a close noncovalent analogue (pdb 5P9I).^39^ This initial investigation aimed to assess the suitability of amide and *N*-aryl linkages, and which of the 4- or 5-position of the pyrimidine would provide more favorable vectors to engage C481. In all computed binding poses (noncovalent docking), the binding mode of biphenyl oxyether and aminopyrimidine-pyrazole motifs was virtually identical to that observed in the crystal structures. Unsurprisingly, we observed more variability for the position, binding mode, and interactions formed by the piperidine-warhead motifs. This part of the ligand is located in the solvent-exposed region and is more flexible, this has been previously discussed by the Mulholland group in the QM/MM molecular dynamics reaction simulations of Ibrutinib (**1g**) and BTK.^37^ The main emerging features from our docking studies were: The 4-substituted derivatives were the highest ranking, positioning the reactive carbon at an average shorter distance to the cysteine sulfur. They consistently engaged in hydrogen bond formation with the C481 thiol via the pyrimidine sp2 nitrogen, and also via the sulfonyl group. At the same time, the sulfonyl group engaged in additional hydrogen bonding with N484 spatially adjacent to C481 (Figure 1H). We also explored a number of substitution patterns at position 5 of the pyrimidine ring. An amide substituent at this position suggested several polar interactions potentially targetable, with the NH forming a new hydrogen bond with the backbone carbonyl of L408 while the *N*-alkyl substituent engaged in hydrophobic contacts with the side chains of L408 and V416 (Figure 1I). The amide carbonyl of the ligand sits at ca. 3 Å from the ammonium group of K430, suggesting potential for hydrogen bonding. In contrast, 5-substituted derivatives did not reproducibly produce poses where the warhead is in suitable distance of C481, but rather engaged in polar interactions with the ammonium group K430 and/or the backbone NH of Q412 via their sulfonyl group, though with more variability.

### Chemical Synthesis

**Scheme 1:**
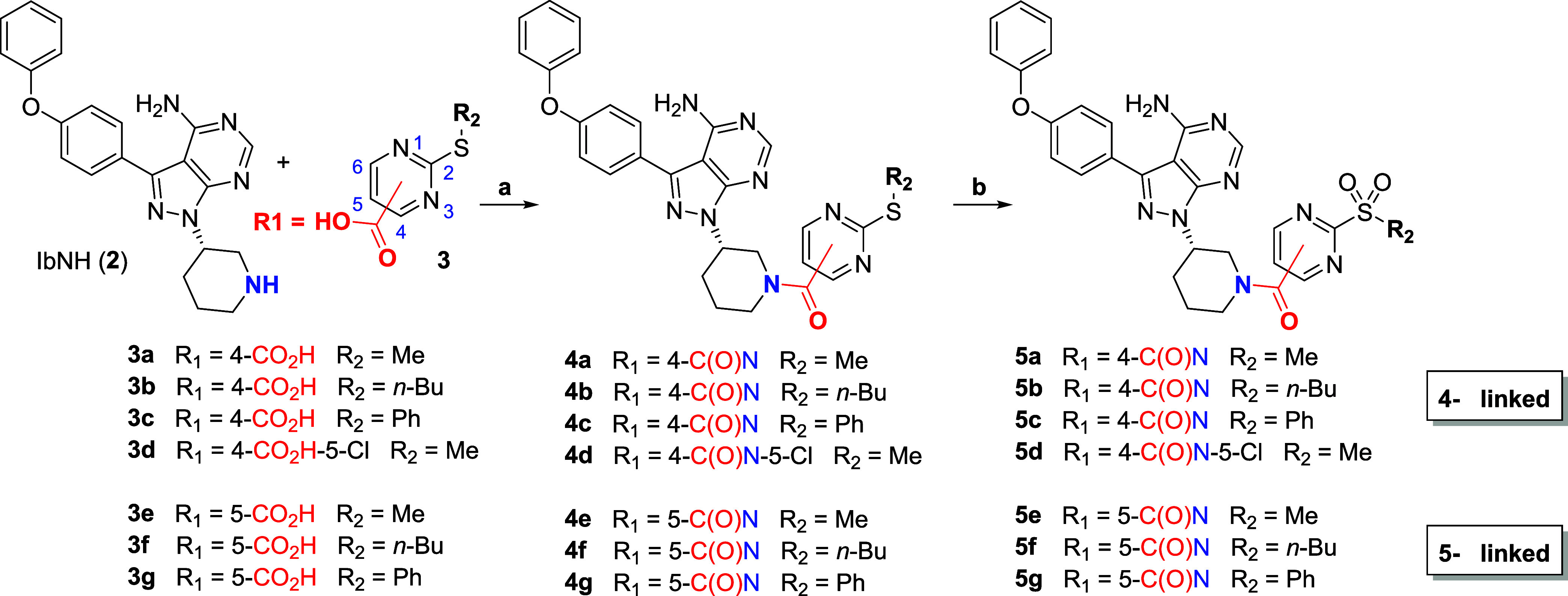
Chemical Synthesis, Amide coupling and 3-step protection/oxidation/deprotection strategy for the synthesis of Ibrutinib derivatives functionalized with representative 2-sulfonylpyrimidine warheads. Isolated yields are provided in the caption. **2** and **3d** were commercially available. Conditions: (a) HATU, Et_3_N, DMF, rt, 16 h, 42–83%; (b) i/Boc_2_O (3.0 equiv, double Boc protection), DMAP, CH_2_Cl_2_, rt, 16 h; ii/ *m*-CPBA, CH_2_Cl_2_, rt, 2 h; iii/TFA, CH_2_Cl_2_, rt, 4 h, 17–75% over three steps.

**Scheme 2:**
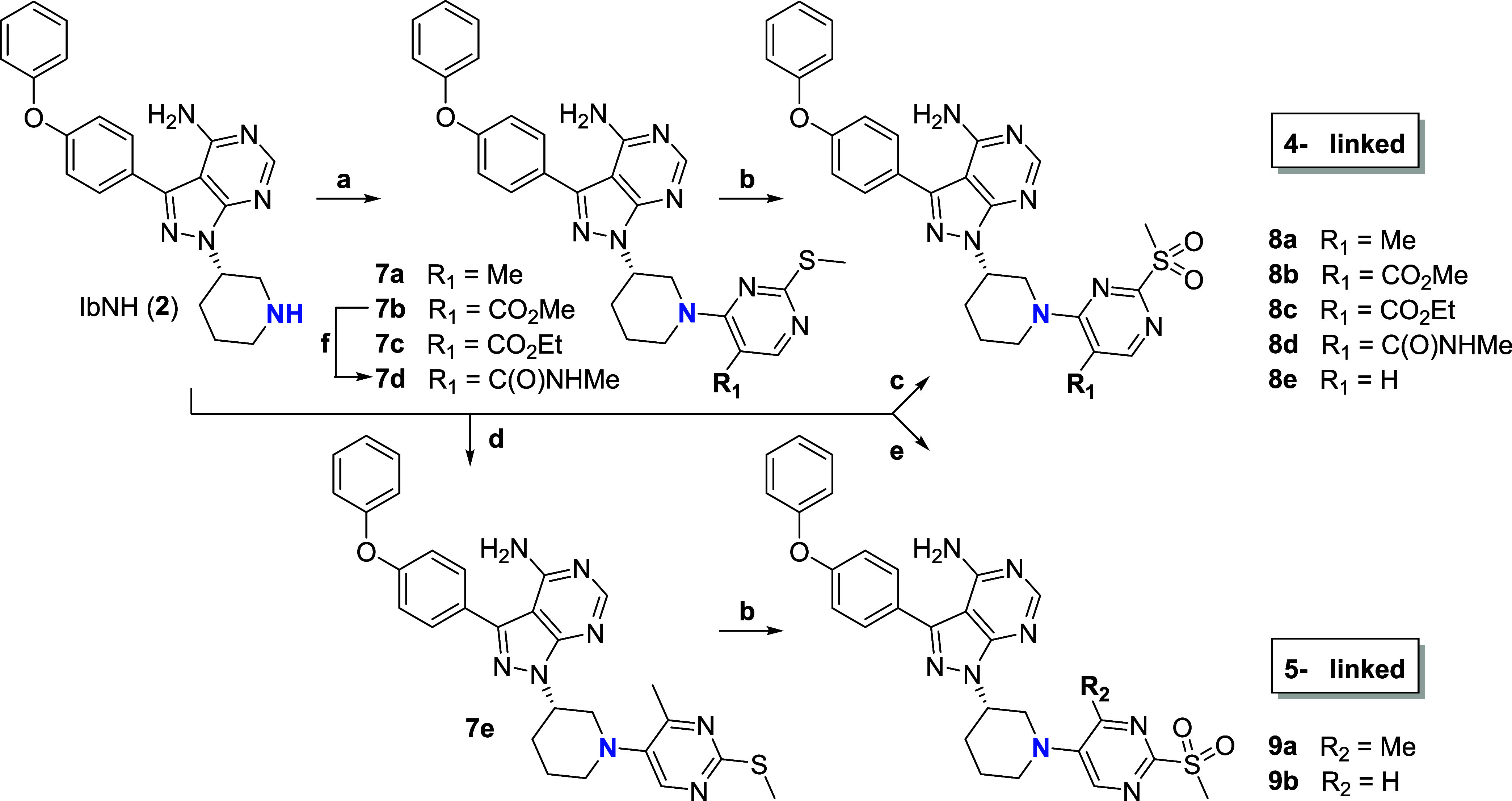
Chemical Synthesis, S_N_Ar, cross-coupling and 3-step protection/oxidation/deprotection strategy to access N-arylated Ibrutinib derivatives functionalized with representative 2-methylsulfonylpyrimidine warheads. Isolated yields are provided in the caption. See Scheme 1 for numbering of positions on the pyrimidine ring. Conditions: (a) pyrimidines **6a**–**c**, Et_3_N, DMF, rt, 16 h, 30–93%; (b) i/Boc_2_O, DMAP, CH_2_Cl_2_, rt, 16 h; ii/*m*-CPBA, CH_2_Cl_2_, rt, 2h; iii/TFA, CH_2_Cl_2_, rt, 4h, 15–46% over three steps; (c) 4-chloro-2-(methylsulfonyl)pyrimidine, CHCl_3_, rt, 16h, 74%; (d) 5-bromo-4-methyl-2-(methylthio)pyrimidine, Pd_2_(dba)_3_, XantPhos, *t*-BuONa, toluene, 100 °C, 16 h, 77%; (e) 5-fluoro-2-(methylsulfonyl)pyrimidine, CHCl_3_, rt, 16 h, 17%; (f) i/10% NaOH, THF, reflux, 4 h; ii/HATU, Et_3_N, MeNH_2_, DMF, rt, 16 h, (89% over 2 steps).

**Scheme 3:**
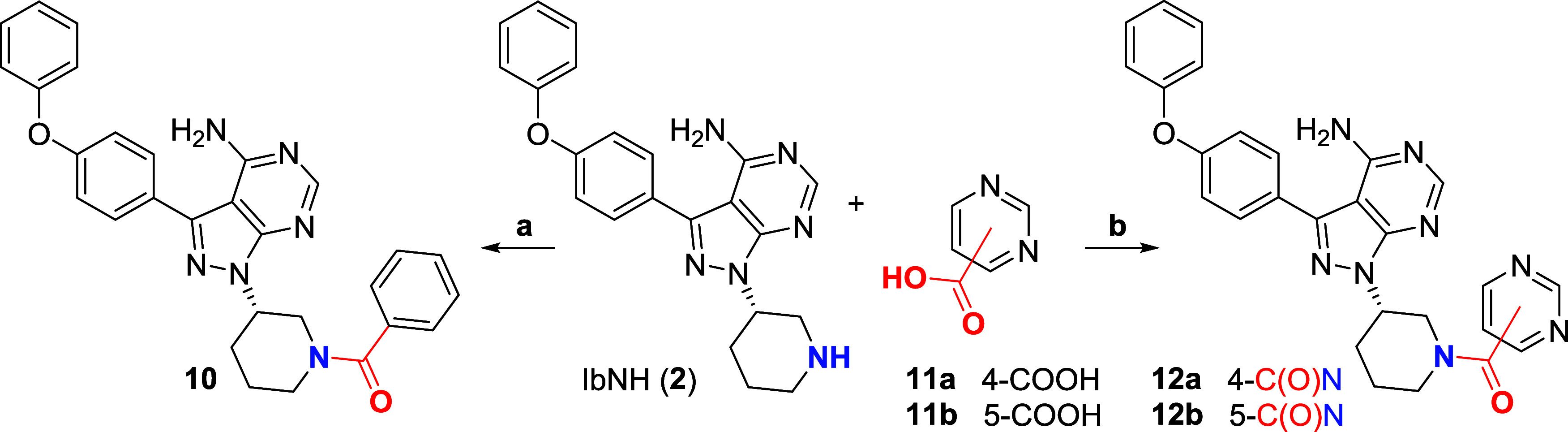
**Chemical Synthesis**, Preparation and isolated yields of noncovalent control compounds lacking the sulfonyl leaving group at position 2. See Scheme 1 for numbering of positions on the pyrimidine ring. Conditions: (a) Bz_2_O, Et_3_N, CH_2_Cl_2_, 16 h, 90%; (b) HATU, Et_3_N, DMF, rt, 16 h, 44–71%.

Amide bond formation between 2-sulfanyl-4/5-carboxyl pyrimidines **3a**–**g** and (*R*)-3-(4-phenoxyphenyl)-1-(piperidin-3-yl)-1H-pyrazolo[3,4-*d*]pyrimidin-4-amine (IbNH, **2**) using HATU afforded intermediates **4a**–**g**. One-pot Boc protection-oxidation-deprotection of the thioether intermediates delivered the corresponding 2-sulfonylpyrimidine derivatives **5a**–**g** in moderate to high yields (Scheme 1). We found that protection of the 4-aminopyrimidine core was necessary as we observed competitive *N*-oxide formation when treating unprotected intermediates **4a**–**g** with diverse oxidizing agents. 4- and 5-halo pyrimidine precursors **6a**–**g** were coupled to **2** by S_*N*_Ar or cross-coupling and converted to *N*-arylated derivatives **8a**–**e** and **9a,b** in a similar manner (Scheme 2). Benzoylation of **2** using benzoic anhydride afforded **10**, while HATU-mediated coupling of 4- and 5-carboxypyrimidines **11a**,**b** afforded **12a,b** (Scheme 3). These derivatives are not substituted at the 2-position and were expected to exhibit reduced activity due to the absence of the sulfonyl leaving group hence noncovalent binding. The preparation of the pyrimidine building blocks **3a**–**g**, **6a**, and **6e** is detailed in the Supporting Information section. Pyrimidines **6b**, **6c**, **11a**, and **11b** are commercially available.

### *In Vitro* Activity

**Figure 2:**
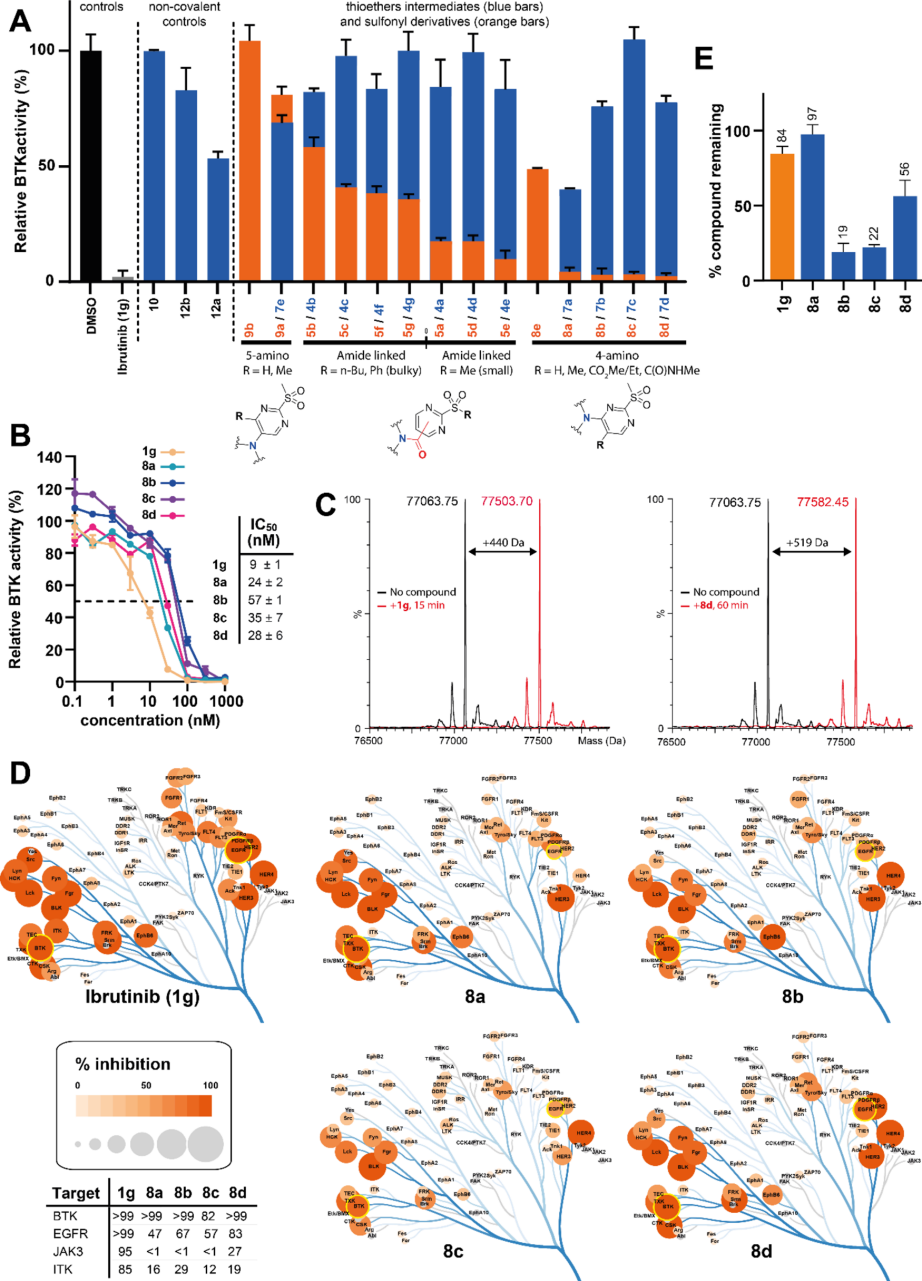
BTK engagement by Ibrutinib (**1g**) and synthetic derivatives *in vitro*: (A) Inhibition of poly-Glu:Tyr in *in vitro* phosphorylation by BTK by 2-SP functionalized Ibrutinib analogues. Relative BTK activity (%, mean ± range, 2–12 replicates) in the presence of compounds (single concentration, 100 nM) normalized against the DMSO control. Note: (tris(2-carboxyethyl)phosphine) (TCEP) was used as a reducing agent in the assay in place of thiol-based ones, to prevent reaction and inactivation of the 2-SP warheads; (**B**) BTK dose–response inhibition and IC_50_ determination for Ibrutinib (**1g**) and derivatives **8a**–**d**. Top panel: % remaining BTK activity for all compounds in a concentration range of 0.1–1000 nM in duplicate (top panel) and IC_50_ calculation (bottom panel); (**C**) modification of intact full-length WT BTK by Ibrutinib (**1g**, left panel) and derivative **8d** (right panel). Data for **8a**–**c** are presented in the Supporting Information, along with all data on the corresponding C481S mutant; and (D) percentage inhibition (ScanTK assay) of a panel of 135 tyrosine kinases by Ibrutinib (**1g**) and new synthetic derivatives **8a**–**d**. (E) Human plasma stability (% remaining) of Ibrutinib (**1g**) and **8a**–**d** after 3 h incubation time at 37 °C.

We first screened all compounds for their ability to inhibit recombinantly expressed wild-type (WT) BTK activity *in vitro* at a fixed compound concentration of 100 nM (Figure 2A, Table S1A). Pleasingly, 2-sulfonyl derivatives (orange) generally displayed high potency, with approximately half of them displaying >80% inhibition of BTK activity. In contrast and as anticipated, the corresponding thioether analogues and noncovalent control compounds (blue) showed comparatively low BTK inhibition, in line with their reversible binding (*vide infra*). Among covalent 2-SP derivatives, the emerging SAR clearly highlighted the four *N*-arylated derivatives (**8a**–**d**) carrying substituents at the 5-position as the most active of the entire series, inhibiting BTK activity by >95%, hence in par with Ibrutinib (**1g**) at the same concentration. Derivatives **5a**, **5d**, and **5e**, connected by an amide linker at either 4- or 5-position of the pyrimidine ring, were slightly less active and inhibited BTK by approximately 80–90%. It is worth noting that their analogues bearing bulky *n*-butyl or phenyl exocyclic substituents **5b**, **5c**, **5f**, and **5g** showed significantly lower inhibitory potency (ca. 40–60% inhibition), consistent with the additional steric constraints imposed at the reactive center.^30^ Derivatives **9,b***N*-arylated at the 5-position were the least potent of the 2-SP series. Again, this is in line with our previous report showing that introducing + M EDGs (e.g., amino, oxyether) at the 5-position of the pyrimidine drastically reduces the reactivity of 2-SP derivatives.^30^ This is also broadly consistent with our docking studies, which suggested additional interactions of the sulfonyl group with the N484 side chain for derivatives arylated at the 4-position of the 2-SP (*vide supra*, Figure 1H). This may in part explain the increased potency of these *N*-arylated analogues and the wider *in vitro* SAR we observed; however, it will be interesting to apply molecular dynamics to these systems in the future and elucidate the precise binding mechanism of these 2-SP derivatives to BTK. While BTK covalent modification by Ibrutinib (**1g**) is well-established, computational studies have only recently highlighted potential mechanistic subtleties, notably associated with proton transfer steps leading to activation of both the nucleophile and electrophile.^37^ In the future it will be interesting to investigate whether 2-SPs and heteroaryl sulfones more generally follow a similar reaction path.

We selected *N*-arylated derivatives **8a**–**d** for titration experiments to determine their IC_50_ values for inhibition of BTK and benchmarked them against Ibrutinib (**1g**) (Figure 2B). Ibrutinib (**1g**) had a mean IC_50_ of 9 nM, which is in line with previous reports.^40,41^ Pleasingly, **8a**–**d** exhibited similar though slightly lower potencies, with IC_50_s ranging from ca. 20–60 nM under the same conditions. Conversely, **8a**–**d** showed little to no activity under the same conditions against the C481S mutant of BTK (Table S1B), lacking the nucleophilic cysteine at position 481. Overall, **8a**–**d** displayed a similar profile to Ibrutinib (**1g**) against wild-type and C481S mutant BTK, consistent with a covalent mode of action toward C481.

We unambiguously confirmed the covalent mechanism of **8a**–**d** by mass spectrometry (Figure 2C, Figure S1). Working with a 2:1 ratio of compound to protein, Ibrutinib (**1g**, control) and **8a**–**d** all modified full-length BTK, albeit at various rates, with evidence for a single modification as measured by mass increases corresponding to each compound tested. Compound **8d**, in particular, induced clean and quantitative modification in under an hour. Conversely, there was no evidence that compounds **8a**–**d** modified the full-length C481S BTK mutant.

Finally, we characterized the inhibition kinetics of Ibrutinib (**1g**), **8a**, **8b** (as ester representative), and **8d** biochemically (Table 1). (**g** showed the highest *K*_inact_/K_i_ (8.16 × 10^6^ M^–1^.s^–1^), mirrored by the highest *K*_inact_ (18.4 × 10^–3^ s^–1^) and the lowest *K*_i_ (2.25 × 10^–9^ M). These values are consistent with previous reports.^42^**8d** showed a potency approaching that of **1g**, with only 3-fold reduction of *K*_inact_/*K*_i_, while **8a** and **8b** were significantly less active and showed 10–20-fold reduced potency. Overall, the 2-sulfonyl-5-amido motif of **8d** emerged as an effective acrylamide replacement, broadly maintaining compound potency both in terms of reversible binding and covalent reactivity toward BTK, at least *in vitro*.

**Table 1:** *K*_inact_/*K*_i_ Determination against BTK for Ibrutinib (**1g**), **8a**, **8b**, and **8d**

	*K*_inact_ (s^–1^)	*K*_i_ (nM)	*K*_inact_/*K*_i_ (M^–1·^s^–1^)
**1g**	0.01836 ± 0.00166	2.25 ± 0.24	8,160,000 ± 175,000
**8a**	0.00659 ± 0.00019	9.08 ± 0.41	726,000 ± 16,900
**8b**	0.00792 ± 0.00019	24.2 ± 0.95	328,000 ± 7,130
**8d**	0.01264 ± 0.00067	5.07 ± 0.36	2,490,000 ± 67,000

### Inhibitor Selectivity

Ibrutinib (**1g**) is known to exhibit off-target inhibition of other kinases, many of which have a cysteine homologous to C481 of BTK.^43,44^ Recently, Buhimschi/Crews and colleagues showed that Ibrutinib (**1g**) exhibits its most significant *in vitro* off-target activity within the TK family in KINOMEscan experiments (Eurofins, scanMAX), while displaying comparatively little off-target across other kinase families.^45^ 1 μM Ibrutinib (**1g**) showed ≥90 inhibition of 39 out of 468 (ca. 8%) human kinases scrutinized, with 36 (including BTK) belonging to the TK family.

To assess *in vitro* target specificity, we measured binding using the KINOMEscan platform (Eurofins, ScanTK) to 135 TKs by new synthetic derivatives **8a**–**d** or Ibrutinib (**1g**), at the same concentration of 1 μM (Figure 2D, Tables S2A,B). The KINOMEscan assay design and principle have been reported previously.^46^ While significantly higher than the *K*_i_/IC_50_ of Ibrutinib (**1g**), Buhimschi/Crews and co-workers showed that it offers good contrast in the data for selectivity assessment.^45^ We reasoned that i/this would allow us to make a useful comparison of our data on Ibrutinib with those previously published, and ii/benchmark the selectivity of **8a**–**d** against Ibrutinib across the TK family. Consistent with previous reports^45^ (Table S2B,C), Ibrutinib (**1g**) exhibited potent off-target binding to multiple kinases beyond BTK, including EGFR, ERBB2–4, SRMS, and BLK showing >99% inhibition under these conditions (Table S2 Bi-ii, columns C and M). Overall, our kinome inhibition data broadly correlated with previous reports (Table S2Biii).^45^**8a**–**d** maintained potent inhibition of BTK, on a par with Ibrutinib (**1g**), and globally reduced off-target effects among top targets (Figure 2D, Table S2Bi,Biv). The most notable difference was a reduced inhibition of WT and mutant EGFR. While Ibrutinib (**1g**) potently inhibited WT EGFR (>99%) and its diverse mutants (89% to >99%), **8a**–**d** showed comparatively reduced inhibition. Also while Ibrutinib (**1g**) exhibited strong inhibition of JAK3 (95%) and ITK (85%), low to no inhibition was observed with **8a**–**d**. As the ScanTK assay was run at a relatively high compound concentration (1 μM), we looked at inhibition at lower compound concentration (Table 2). IC_50_ determination (no preincubation time) confirmed that while Ibrutinib (**1g**) potently inhibits EGFR, pleasingly none of the compounds, **8a**, **8b**, or **8d**, exhibited measurable inhibition. Also, none of the compounds inhibited JAK3 and ITK in these conditions. Rationalizing the reduced inhibition of EGFR by **8a**, **8b**, and **8d** will require further investigation in the future to reveal the factors at play. Different amino acids are found at the *i*+3 position among kinases that contain cysteine at the equivalent position to C481 in BTK, although most are Asn or Asp. Residue 484 in BTK is Asn, while the corresponding *i*+3 residue is Asp in EGFR. QM/MM molecular dynamics reaction simulations have suggested that the difference in basicity between Asn and Asp may be an important factor influencing the microenvironment and formation/stabilization of the reactive thiolate, along with distinct proton transfer steps and overall mechanisms for BTK and EGFR inhibition.^37^ This may be a contributing factor to the altered profiles of **8a**–**d** toward EGFR and its mutants. It is also worth noting that the sulfonylpyrimidine motif itself is a hydrogen bond acceptor and is slightly basic. While we have previously reported *in vacuo* DFT calculations on 2-SPs and model methanethiolate,^30^ it will be interesting in the future to investigate these systems in a dynamic and solvated environment to provide a more detailed picture of the inhibition mechanism, especially proton transfer steps.

**Table 2:** IC_50_ Determination against BTK/EGFR/JAK3/ITK for Ibrutinib (**1g**), **8a**, **8b**, and **8d**

	1 nM BTK			3 nM EGFR	2 nM JAK3	10 nM ITK
	0 min preincubation IC_50_ (nM)	60 min preincubation IC_50_ (nM)	fold change	0 min IC_50_ (nM)	0 min IC_50_ (nM)	0 min IC_50_ (nM)
**1g**	0.62	0.10	6.1	59	>1000	>1000
**8a**	6.0	0.31	19.6	>1000	>1000	>1000
**8b**	79	6.3	12.5	>1000	>1000	>1000
**8d**	3.6	1.2	3.1	>1000	>1000	>1000

### Plasma Stability and Intrinsic Reactivity

We then investigated the impact of the new warheads on the plasma stability of **8a**–**d** vs Ibrutinib (**1g**) by UPLC-MS quantification following a 3h incubation period at 37 °C. 5-alkylester **8b** and **8c** and 5-amido derivative **8d**, overall showed reduced plasma stability compared to Ibrutinib (**1g**), with ∼20–50% remaining postincubation. This is likely resulting, at least in part, from susceptibility to hydrolase-mediated cleavage. GST-catalyzed GSH conjugation to a variety of electrophiles, including halo-nitroarenes, has been well-documented and may also play a role here. In contrast, *N*-arylated analogue **8a** was fully stable in the same conditions, indeed outperforming Ibrutinib (**1g**) (Figure 2E). Also, and similarly to Ibrutinib, **8a** was fully stable when incubated with a 10-fold excess of GSH in PBS, with no evidence of covalent adduct even after up to 6 h at 20 °C, as monitored by LC-MS. **8d** however showed mild reactivity in the same conditions, with a measured pseudo-first-order rate constant *k*_GSH/PBS_ = 47 × 10^–5^ ± 0.5 × 10^–5^ s^–1^ (triplicate). This is broadly in line with the reactivity trends we have previously reported on the corresponding isolated warheads.^30^

### Cell-Based Activity

For analysis of activity in the cells, we investigated the effects of compounds on B-cell receptor (BCR) signaling in B lymphoma cells focusing on BTK’s role in Ca^2+^ mobilization.^33,47^ BTK is activated following BCR ligation in a two-step process that involves phosphorylation on Y^551^ by proximal kinases followed by autophosphorylation on Y^223^. The main substrate for BTK is PLCγ2 which catalyzes the conversion of the plasma membrane lipid PIP_2_ into diacylglycerol (DAG) and IP_3_.^48−50^ IP_3_ triggers the release of Ca^2+^ from the endoplasmic reticulum (and the subsequent influx of extracellular Ca^2+^ via SOCS). PLCγ2 can also be activated directly by SYK^48^ and, in some cells, the initial phase of Ca^2+^ release is mediated by SYK, whereas BTK is required to sustain Ca^2+^ levels.^51,52^ Increased intracellular Ca^2+^, together with DAG, stimulates protein kinase C (PKC) isoforms leading to the activation of MAPKs and NF-κB.^53^ Increased Ca^2+^ also leads to nuclear translocation of nuclear factor of activated T-cells (NFAT), and NF-κB and NFAT in turn induce the expression of target genes, which promote B-cell survival and proliferation (Figure S2).^54−57^

**Figure 3:**
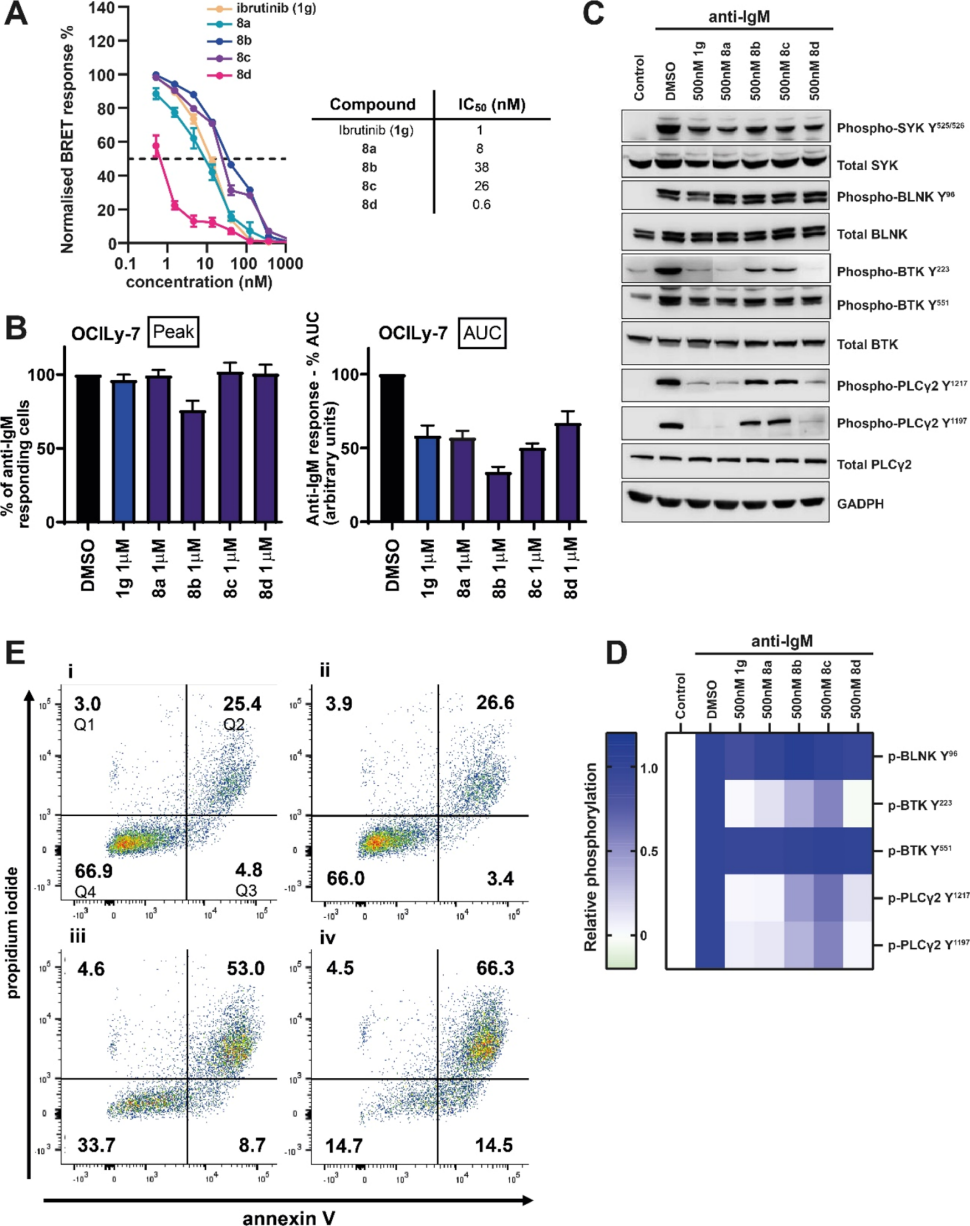
Target engagement in cells and mechanistic studies: (A) BTK dose–response inhibition and IC_50_ determinations for Ibrutinib (**1g**) and derivatives **8a**–**d**, determined by NanoBRET. Left panel: % remaining BTK activity in HEK293 cells for all the compounds at a concentration range of 1–1000 nM by duplicate (left panel) and IC_50_ calculation (right panel). (B) Effect of compounds on anti-IgM-induced signaling in OCI-LY7 cells. The cells were pretreated with compounds (1000 nM) or DMSO for 1 h before analysis of anti-IgM-induced Ca^2+^ fluxes. Figures show representative results (from 3 to 7 separate determinations) for effects on peak (maximum number of responding cells) and duration (area under the curve, AUC). (C) OCI-LY7 cells were pretreated with the indicated compounds (500 nM) or DMSO for 1 h and then treated with anti-IgM or control antibody for 30 s. Expression of the indicated proteins was analyzed by immunoblotting. Representative results (**C**) and heat map (**D**) show relative phosphorylation with values for anti-IgM/DMSO-treated cells set to 1.0 (from 2 or 3 independent experiments). See Figure S3A for additional quantification. (E) Apoptotic activity: TMD8 cells were treated with compounds for 72 h before cell viability was analyzed using annexin V/PI staining. Representative results for cells treated with DMSO (i) or compound **8d** at 10 (ii), 100 (iii), or 1000 nM (iv); See Figure S4 for additional quantification.

First, we used the NanoBRET cellular target engagement assay to directly quantify the occupancy of BTK in intact cells (HEK293) by Ibrutinib (**1g**) and **8a**–**d**.^58^ All compounds showed potent engagement of BTK, with Ibrutinib (**1g**), **8a**, and **8d** being the most potent and displaying low to subnanomolar IC_50_s (Figure 3A).

The effects of compounds on Ca^2+^ release were then analyzed following treatment of OCI-LY7 cells (derived from a diffuse large B-cell lymphoma (DLBCL)) with anti-IgM to cross-link surface IgM (sIgM). We recently showed that in these cells, BTK inhibition accelerates the decline in Ca^2+^ levels following its initial peak, consistent with the principal role for BTK in sustaining Ca^2+^ release in these cells.^38,59^ Consistent with BTK inhibition, the compounds generally had a very similar effect to Ibrutinib (**1g**) as they accelerated the decline in Ca^2+^ levels with only modest effects on the initial peak response which occurs independently of BTK (Figure 3B, Figure S3A). For **8a** and **8d**, we performed similar experiments at lower concentrations. The acceleration of the decline in Ca^2+^ flux was maintained for these compounds at concentrations down to 250 nM (Figure S3B).

We also investigated the effects of the compounds (at 500 nM) on sIgM signaling by analyzing the phosphorylation status of key signaling molecules activated downstream of the BCR. We first investigated phosphorylation of BTK on Y^551^ and Y^223^ which is mediated by SYK or BTK (i.e., autophosphorylation), respectively (Figure 3C,D and S3A). As expected, Ibrutinib (**1g**) strongly inhibited anti-IgM-induced BTK Y^223^ but not Y^551^ phosphorylation. Similar results were obtained for **8a** and **8d**. Ester functionalized **8b** and **8c** also selectively reduced BTK Y^223^ phosphorylation although their effects were not as dramatic as for **8a** and **8d**, despite similar *in vitro* potencies (Figure 2A). This is consistent with their higher cellular IC_50_s (Figure 3A), possibly due to *in situ* ester hydrolysis, again consistent with plasma stability data (Figure 2E). On-target inhibition of BCR signaling was confirmed by analysis of additional upstream (BLNK Y^96^) and downstream (PLCγ2 Y^1197^ and Y^1217^) phosphorylation events, which were unaffected or inhibited, respectively, by the compounds (Figure 3**C,D** and Figure S3A). Overall, these results show that the compounds effectively inhibit BTK in intact cells with little, if any, off-target effect on upstream kinases.

### Apoptosis

We tested the effects of compounds in TMD8 cells, derived from an activated B-cell DLBCL. These cells have constitutive signaling via the BCR and additionally carry *MYD88*^*L265P*^ and *CD79B* mutations which give rise to My-T-BCR complex. This appears to confer a high degree of susceptibility to BTK-induced apoptosis and clinical responses to Ibrutinib (**1g**) in treated DLBCL patients.^60^ Apoptosis was quantified after exposure of cells to compounds for 72 h using annexin V/PI staining and flow cytometry (Figure 3E, Figure S4). An initial screen demonstrated that the compounds **8a**, **8b**, and **8d** induced apoptosis in TMD8 cells and that the relative activity of these compounds appeared to correlate well with their *in vitro* potency (**8a** ≈ **8d** > **8b**) (Figure S4). However, **8c** appeared to be essentially inactive in this assay. Repeat experiments using the two most potent compounds **8a** and **8d** confirmed that they induced apoptosis of TMD8 cells, but their potency appeared to be ∼10-fold less than Ibrutinib (Figure S4).

### Gene Expression

**Figure 4:**
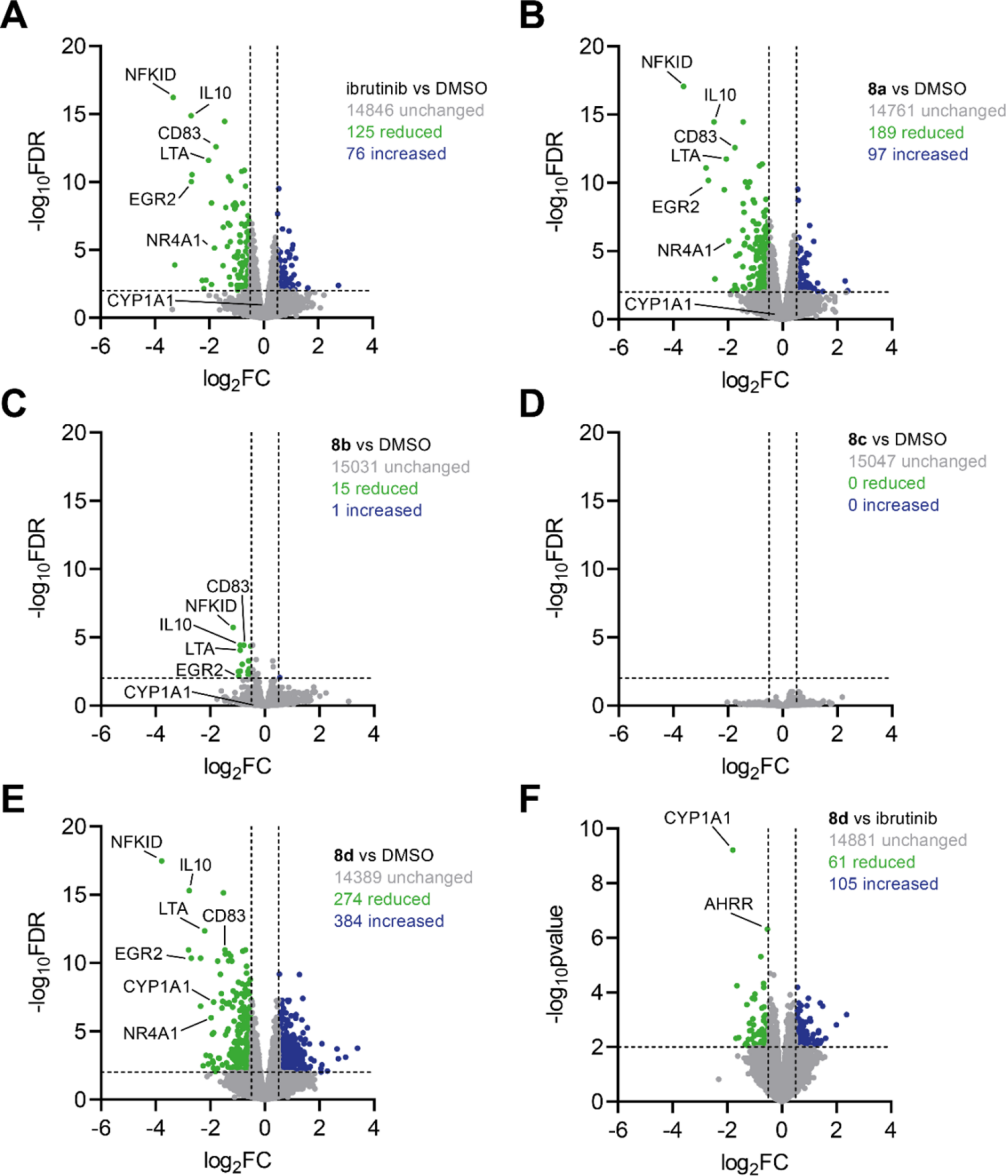
Effect of compounds on gene expression in TMD8 cells. Volcano plots showing changes in gene expression in TMD8 cells in response to treatment (1 μM, 8-h incubation) with (A) Ibrutinib (**1g**), (B) **8a**, (C) **8b**, (D) **8c**, and (E) **8d**. Difference in gene expression profiles between **8d** and Ibrutinib (**1g**). In all panels, significantly downregulated genes are colored green and significantly upregulated genes are colored blue. Cut-offs are log_2_FC ← 0.5/>0.5 and FDR < 0.01 (**A**–**E**) or *p*-value <0.01 (F).

We performed RNA-seq to investigate the effects of Ibrutinib (**1g**) and **8a**–**d** (1 μM for 8 h) on gene expression in TMD8 cells. Ibrutinib significantly downregulated (FDR < 0.01; log_2_FC ← 0.5) and upregulated (FDR < 0.01; log_2_FC > 0.5) the expression of 125 and 76 genes, respectively (Figure 4; significantly regulated genes for all compounds are listed in Table S4). Consistent with the inhibition of BCR signaling the most strongly downregulated genes (by log_2_FC) included *NFKBID*, *LTA, EGR2*, *CD83*, and *NR4A1*, which we previously identified as being induced by anti-IgM in primary chronic lymphocytic leukemia cells,^61^ and *IL-10*, which has been shown to be downregulated by Ibrutinib (**1g**) in DLBCL cells.^62^ Pathway analysis demonstrated that the Ibrutinib (**1g**) signature was very strongly enriched for pathways associated with cell signaling (especially those related to NF-κB, a downstream target of BTK) and that these pathways were generally predicted to be repressed (Table S3A). Moreover, the strongest predicted upstream regulator was an immunoglobulin which was predicted to be inhibited in Ibrutinib-treated cells (Table S3B).

The effects of **8a** on gene expression were very similar to Ibrutinib (**1g**) in terms of number of genes regulated and a bias toward downregulation (Figure 4B). **8b** also induced significant changes in gene expression. However, the extent of regulation was very modest compared to Ibrutinib (**1g**) and **8a** (Figure 4C), and there were no genes that were significantly regulated by **8c** (Figure 4D). Interestingly, **8d** regulated a greater number of genes compared to Ibrutinib (**1g**) and **8a** (Figure 4E) and there was a bias toward increased expression, not downregulation. Despite these differences, interrogation of selected Ibrutinib-regulated genes (*NFKBID*, etc.) confirmed that all compounds (with the exception of **8c**) regulated a set of overlapping target genes. Indeed, GSEA showed an extremely high correlation between gene expression changes by Ibrutinib (**1g**) and each of the other (active) compounds (Figure S5). Thus, with the exception of **8c**, all tested compounds regulated a similar set of target genes as Ibrutinib (although the extent of the effect differed between compounds), and these genes were clearly related to the inhibition of BCR signaling.

Since **8d** regulated the expression of more genes than Ibrutinib (**1g**), we identified genes that were differentially expressed between Ibrutinib (**1g**) and **8d**-treated cells to probe the potential distinct effects of **8d** on gene expression. Differences in gene expression were more modest compared to those between DMSO and drug-treated cells and we therefore relaxed the cutoff for statistical significance in this analysis (p-value <0.01) (Figure 4F). Analysis of downregulated genes identified significant enrichment of pathways associated with the aryl hydrocarbon receptor (AHR) (Table S4A**)**, a transcription factor that induces expression of cytochrome P450 enzymes in response to xenobiotics but also has important functions in immunity and cell homeostasis in response to endogenous ligands.^63^ Indeed, the key AHR target gene *CYP1A1* (and to a lesser extent *AHRR*) was significantly downregulated by **8d** but not any of the other compounds analyzed. Moreover, AHR was identified as an upstream regulator mediating the selective, repressive effect of **8d** on gene expression (Table S4B**)**. AHR is a repressor of noncanonical cell death pathways^64−66^ and consistent with this, genes that were selectively induced by **8d** in TMD8 cells were enriched for necroptosis and pyroptosis (Table S4C**)**.

## Conclusions

Acrylamide-based warheads still dominate modern TCI design strategies. Here, we investigated the 2-sulfonylpyrimidine moiety as a surrogate for the acrylamide group, using Bruton’s tyrosine kinase as a model system. We present the *in vitro* and cellular activity of focused libraries of 2-SP functionalized Ibrutinib derivatives, along with the design strategy and synthetic routes deployed in the process. We show that several *N*-arylated 2-SP functionalized derivatives engage BTK *in vitro* and *in cellulo* with potencies on par with parent Ibrutinib (**1g**). Interestingly several derivatives maintained strong BTK inhibition while displaying less pronounced off-target engagement across a panel of 135 tyrosine kinases. The precise reason(s) for this will require further structural insight into the future, but our initial hypothesis driving the molecular design was that the increased size/sterics of the 2-SP motif combined with the directional trajectory of the cysteine nucleophile in the S_N_Ar process may impose additional constraints on the reaction coordinates when targeting distinct enzyme active sites, hence influence downstream selectivity profiles. This is an interesting finding given the inherent challenge in achieving selectivity in the field of kinases, and 2-SP warheads seem well-positioned to impact other areas beyond kinases. There were differences in plasma stability of the compounds with **8a** outperforming Ibrutinib (**1g**). By contrast, **8b** and **8c** appeared to be more susceptible to degradation and this may explain why they exhibited reduced/lack of inhibitory (signaling and gene expression) or pro-apoptotic activity where cells are exposed to compound for various durations.

Prototypical compounds **8a** and **8d** showed very similar signatures to that of Ibrutinib (**1g**) in RNA-seq experiments. The most notable difference was the downregulation of pathways associated with the aryl hydrocarbon receptor (AHR) and downstream cytochrome P450 enzymes by **8d**. Such response to xenobiotics is typically characterized by nuclear translocation of AHR and transcriptional activation of xenobiotic-responsive elements (XRE) in enhancer regions of target genes such as CYP1A1, CYP1A2, and CYP1B1 oxidases.^67^ For unknown reasons, this response appears to be specifically constitutively activated in TMD8 cells, and selectively inhibited by **8d**. Consistently, inhibition of protein tyrosine kinases (e.g., c-*src*) has been shown to modulate AHR activity in hepatoma cells, possibly driven by Tyr320 phosphorylation.^68^ The differences in tyrosine kinase inhibition profiles we have observed *in vitro* are consistent with this model and while the function of AHR is not fully understood and appears to be highly context-dependent, it has been proposed as an attractive target for therapeutic intervention. AHR plays key roles in the pathogenesis of numerous diseases and disorders, including autoimmunity, inflammatory diseases, endocrine disruption, premature aging, and cancer. Whether downregulation of AHR and CYP450 activity is desirable in a BTK context remains an open question, but our data might suggest a better tolerability in cells for **8d** compared to Ibrutinib (**1g**). This study further underlines that differences in warhead types, and in our case subtle differences in warhead decoration, can be harnessed for fine-tuning gene expression profiles and potentially achieve useful poly pharmacologies. Going forward, the detailed mechanistic relationship between kinome inhibition and transcriptional profiles of BTK inhibitors will be an interesting subject for further studies. More broadly, S_N_Ar-based electrophiles are gaining momentum in TCI design, including in the kinase field. While preparing this manuscript, the Gehringer lab reported new inhibitors of the fibroblast growth factor receptor 4 (FGFR4) kinase domain. Lead inhibitors employed a 2-chloro-5-nitropyridine warhead as an acrylamide replacement to covalently target C552 of FGFR4, and showed selectivity over other FGFR isoforms.^69^ This is yet another testament to the potential of S_N_Ar-based electrophiles for TCI design and performance enhancement of existing lead compounds containing more traditional warheads.

## Experimental Section

### Molecular Docking

The crystal structures of BTK in complex with Ibrutinib (**1g**) (pdb 5P9J) and its noncovalent analogue (pdb 5P9I) were prepared (Schrödinger) using the Protein Preparation Wizard^70^ from Schrodinger, and the corresponding receptor grids were generated using Glide.^71^ Ligands were imported to Maestro^72^ as. sdf files, prepared (Ligprep^73^), and docked (Glide, XP precision) in the grids. No constraint was applied to the system, to prevent bias. Docking poses were subjected to one round of Prime minimization,^74^ then analyzed visually with Maestro and Pymol (www.pymol.org).

### Compounds

Ibrutinib (**1g**) was purchased from MedChemExpress. The Ibrutinib core (IbNH, **2**) was purchased from Fluorochem. Synthetic procedures and compound characterization data, representative NMR spectra, and high-performance liquid chromatography traces are described in the Supporting Information section. The purity of the synthetic compounds was ≥95%, as determined by UPLC analysis on a Waters, Acquity UPLC BEH C18 (50.0 mm × 2.10 mm 1.70 μm) column using a gradient elution from 20% acetonitrile (0.2% formic acid) to 100% acetonitrile (0.2% formic acid) over 5 min at 0.6 mL/min.

### Cell Lines

OCI-LY7 cells (kindly provided by Professor Jude Fitzgibbon, Bart’s Cancer Centre UK) were cultured in IDMI medium (ThermoFisher) supplemented with 20% (v/v) fetal bovine serum (FBS; PAN Biotech), penicillin and streptomycin (Penicillin/streptomycin Sigma, ref: P4333 10 mL/L). TMD8 cells (kindly provided by Professor Louis Staudt, National Cancer Institute, US) were cultured in RPMI-1640 medium (Sigma) supplemented with 10% (v/v) FBS, penicillin, streptomycin, and glutamine (Glutamine Sigma, ref: G7513 2 mM) (all from Sigma). Cell line identity was routinely confirmed using short tandem repeat analysis (Powerplex 16 System, Promega) and the absence of mycoplasma was confirmed using the Mycoplasma PCR detection kit (Applied Biological Materials). Cell lines were typically cultured for a maximum of 6–8 weeks. The sIgM stimulation was performed by treating cells with goat antihuman F(ab’)_2_ anti-IgM or control antibody (both 20 μg/mL; Southern Biotech).

### BTK *In Vitro* Activity Assays

Test compounds were preincubated for 10 min at 22 °C with either full-length His-tagged wild-type BTK^75^ (SignalChem, 15 ng) or C^481^S mutant BTK (30 ng; both sourced from Stratech) in kinase buffer I (Stratech) supplemented with 2 mM MnCl_2_, 100 μM Na_3_VO_4_ and either 1 mM TCEP or 2 mM DTT (all from Merck). The reaction was initiated by the addition of 10 μM ATP (Promega) and 5 ng of poly(4:1, Glu:Tyr) peptide (Stratech) and incubated for 1 h at 22 °C. Kinase activity was measured using the ADP-Glo kinase assay (Promega). For IC_50_s serial dilutions were carried out to achieve concentrations between 0.1 nM and 1000 nM from a 10 mM stock solution. Assays were performed in duplicate and IC_50_s were determined using Prism9 (GraphPad Software, La Jolla, CA, USA). IC_50_s were determined in the same manner. Note: We employed tris(2-carboxyethyl)phosphine (TCEP) as the reducing agent in these experiments, as we have previously shown that 2-SPs react covalently with a number of model thiols such as GSH or dithiothreitol (DTT).^30^ As expected, some 2-methylsulfonyl pyrimidine derivatives showed reduced potency in the presence of DTT. For example, this was particularly marked for derivative **5d**, which showed almost no inhibition in the presence of DTT (Figure S6). We observed a similar trend with Ibrutinib (**1g**) and lead *N*-arylated molecules **8a**–**d**, although they still maintained high potency.

### BTK Expression and Purification

Full-length wild-type and mutant BTK were produced by coexpressing with YopH in BL21(DE3) (Millipore Sigma) as described previously.^76^ Briefly, the culture was grown at 37 °C to an O.D. 600 nm of 0.6 to 0.8. The temperature of the culture was lowered to 18 °C and then induced with 0.1 mM IPTG. The culture was harvested 24 h after induction and the pellets were resuspended in lysis buffer (50 mM KH_2_PO_4_, pH 8.0, 150 mM NaCl, 20 mM imidazole, and 0.5 mg/mL lysozyme) and stored at −80 °C. Cells were lysed by thawing and the action of lysozyme, and 3000 U DNase I (Sigma) and 1 mM PMSF were added to the lysate, incubated at RT for 20 min and then spun at 16,000 rpm for 1 h at 4 °C. Glycerol was added to the supernatant to a final concentration of 10% and was then incubated with Ni-NTA resin (QIAGEN) for 2 h, washed with Tris pH 8.0, 75 mM NaCl, 40 mM imidazole, and eluted in 20 mM Tris pH 8.0, 150 mM NaCl, 250 mM Imidazole, and 10% glycerol. Eluted protein was flash-frozen in liquid nitrogen and stored at −80 °C. The proteins were then concentrated and further purified by size exclusion chromatography (Hiload Superdex 26/60 200 pg, GE Healthcare). The fractions containing pure protein were pooled, concentrated, snap-frozen, and stored at −80 °C. The final buffer consists of 20 mM Tris pH 8.0, 150 mM sodium chloride, and 10% glycerol. Initial BTK Y551 phosphorylation levels of purified BTK protein used in this study are below western immuno-detection.

### Protein Mass Spectrometry

Prior to intact mass analysis, purified full-length wild-type or C481S BTK (20 μM) and compound (40 μM), both in 20 mM Tris pH 8.0, 150 mM NaCl, 10% glycerol, 2% DMSO, were allowed to interact at 23 °C for either 15 min, 1, 2, or 4 h depending on the compound and BTK construct (see Figure S1 for details). Both the apo wild-type and C481S BTK, as unmodified controls, and proteins bound to compounds (20 picomoles) were injected into a Waters M-Class HDX system configured for intact mass analyses at 23 °C. The proteins were injected into the sample loop and desalted for 3 min at 100 μL/min using water (0.1% formic acid) on an in-house packed POROS 20R-2 trap. Proteins were eluted into the mass spectrometer using a 15–70% ACN (0.1% formic acid) gradient in 10 min at a flow rate of 100 μL/min. Mass spectra were acquired using a Waters Synapt HDMSE mass spectrometer operated in TOF-only mode with a standard electrospray source, capillary voltage of 3200 V, and a cone voltage of 40 V with a mass range of 50–2000 *m*/*z*. Intact mass values for both free and labeled wild-type BTK were calculated from the raw *m*/*z* spectra using MaxEnt1 within MassLynx 4.1 (Waters) with a resolution of 0.05 Da and an output mass range of 65,000–85 000 Da.

### Biochemical Characterization

Biochemical enzymatic IC_50_ data were generated by AssayQuant Technologies (Marlborough, MA) using the PhosphoSens Platform. IC_50_s were determined using 1 nM BTK, full length (2–659), N-term GST fusion, Carna Biosciences; 3 nM EGFR, cytoplasmic domain (669–1210), N-Term GST fusion, Carna Biosciences; 2 nM JAK3, catalytic domain (795–1124), N-term His-tagged, Carna Biosciences; 10 nM ITK, full length, GST-tagged, Invitrogen. Final reaction conditions: 50 mM HEPES, pH 7.5, 1.0 mM ATP, 1.0 mM DTT, 0.01% Brij-35, 0.5 mM EGTA, 1.0% glycerol, 10 mM MgCl_2_, 0.20 mg/mL BSA, 15 μM AQT sensor substrate (AQT0101; AQT0734), 2% DMSO, to which was added the appropriate inhibitor and kinase. All reactions were run at room temperature in PerkinElmer 384-well, white, low-volume microplates after sealing using optically clear adhesive film (TopSealA-Plus plate seal, PerkinElmer) in a Biotek Synergy Neo 2 microplate reader with excitation (360 nm) and emission (485 nm) wavelengths. Reaction rate versus log [inhibitor concentration] data were fitted to the four-parameter logistic equation
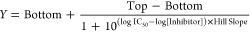


Nonlinear regression was performed using the Solver algorithm in Excel. *K*_i_ values were calculated via the Cheng–Prusoff equation, assuming a reversible, substrate-competitive mode of inhibition:
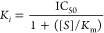
*K*_i_ and *K*_inact_ parameters were determined using 1 nM BTK, full length (2–659), N-term GST fusion, Carna Biosciences. Reaction conditions: 50 mM HEPES, pH 7.5, 1.0 mM ATP, 1.0 mM DTT, 0.01% Brij-35, 0.5 mM EGTA, 1.0% glycerol, 10 mM MgCl_2_, 0.2 mg/mL BSA, 40* μM AQT sensor substrate (AQT0101), 1% DMSO. Reactions were run at room temperature in Corning 384-well White Flat Bottom Polystyrene NBS Microplate after sealing using optically clear adhesive film (TopSealA-Plus plate seal, PerkinElmer) in a Biotek Synergy Neo 2 microplate reader with excitation (360 nm) and emission (485 nm) wavelengths. Two-step *K*_inact_/*K*_i_ fits were extracted from the progress curve analysis of all compounds against BTK, enabling independent *K*_inact_ and *K*_i_ determinations.

### Electrophilic Reactivity Assessment (GSH Assay)

Sample preparation: To PBS (0.01 M phosphate buffer, 0.0027 M potassium chloride, and 0.137 M sodium chloride, pH 7.4) (1020 μL) were successively added the appropriate compound (30 μL from 4 mM DMSO stock, 1 equiv), 4-hydroxybenzoic acid (UV active standard, 30 μL from 4 mM DMSO stock, 1 equiv), and GSH (120 μL from 10 mM stock in same PBS, 10 equiv). Final volume: 1200 μL; final composition: 100 μM compound, 100 μM standard, 1 mM GSH, 5% v/v DMSO. The resulting mixture was kept at 20 °C. Reaction monitoring and rate determination: LC-MS traces of the mixture were acquired every 10 min. Normalized integrations of the starting material and/or product were plotted as a function of time (s), using the 4-hydroxybenzoic acid peak as standard for integral calibration/normalization, allowing extraction of pseudo-first-order reaction rate constants (k’). All measurements were performed in triplicate.

### *In Vitro* Kinase Selectivity Assays

The selectivity of compounds across the kinome was determined using EuroFins ScanTK, at a final compound concentration of 1 μM. For most assays, kinase-tagged T7 phage strains were grown in parallel in 24-well blocks in an *E. coli* host derived from the BL21 strain. *E. coli* were grown to log-phase and infected with T7 phage from a frozen stock (multiplicity of infection = 0.4) and incubated with shaking at 32 °C until lysis (90–150 min). The lysates were centrifuged (6,000*g*) and filtered (0.2 μm) to remove cell debris. The remaining kinases were produced in HEK293 cells and subsequently tagged with DNA for qPCR detection. Streptavidin-coated magnetic beads were treated with biotinylated small molecule “control” ligands for 30 min at room temperature to generate affinity resins for kinase assays. The liganded beads were blocked with excess biotin and washed with blocking buffer (SeaBlock (Pierce), 1% BSA, 0.05% Tween 20, 1 mM DTT) to remove the unbound ligand and to reduce nonspecific phage binding. Binding reactions were assembled by combining kinases, liganded affinity beads, and test compounds in 1x binding buffer (20% SeaBlock, 0.17x PBS, 0.05% Tween 20, 6 mM DTT). Test compounds were prepared as 40x stocks in 100% DMSO and directly diluted to a 1 μM final concentration (2.5% v/v dmso) into the assay. All reactions were performed in polypropylene 384-well plates in a final volume of 0.02 mL. The assay plates were incubated at room temperature with shaking for 1 h and the affinity beads were washed with wash buffer (1× PBS, 0.05% Tween 20). The beads were then resuspended in elution buffer (1× PBS, 0.05% Tween 20, 0.5 μM nonbiotinylated affinity ligand) and incubated at room temperature with shaking for 30 min. The kinase concentration in the eluates was measured by qPCR. The compound(s) were screened at 1 μM, and results for primary screen binding interactions are reported as ’%Ctrl’, where lower numbers indicate stronger inhibition. %Ctrl values were calculated as follows
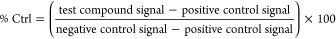

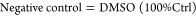

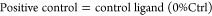


### Ca^2+^ Flux Assays

OCI-LY7 cells were cultured in the presence of indicated concentration of compounds or DMSO for 1 h, incubated with 4 μM Fluo3-AM (Life Technologies) and 0.02% (v/v) Pluronic F-127 (Sigma) for 30 min at 37 °C and compounds were readded following washing. Flow cytometry was performed using a FACS Canto II system (BD Biosciences). Data was acquired for 30 s before cells were stimulated with goat antihuman F(ab’)_2_ anti-IgM and data were acquired for a further 3.5 min. Cells were then treated with ionomycin (1 μM; Merck) and data were acquired for 2 min. Calcium responses were quantified by calculating area under the curve (AUC) in FlowJo (Becton Dickinson) from the point of addition of anti-IgM to the addition of ionomycin.

### Immunoblotting

SDS-PAGE was performed using equal protein loading (15–30 μg) following quantitation of protein content using the BioRad Protein Assay and with the following antibodies; anti-BLNK, anti-Y^96^ phosphorylated-BLNK, anti-BTK, and anti-Y^223^ phosphorylated BTK (all from Cell Signaling Technology), anti-Y^551^ phosphorylated BTK, anti-Y^1197^ phosphorylated-PLCγ2 and anti-Y^1217^ phosphorylated-PLCγ2 (all from Abcam) and anti-GAPDH (Invitrogen). Secondary antibodies were horseradish peroxidase-conjugated antibodies (GE Healthcare). Images were captured using the Amersham ImageQuant 800 System and quantified using ImageJ (http://imagej.nih.gov/ij/). Expression of phosphorylated proteins was normalized to the equivalent total protein with values for DMSO/anti-IgM set to 100%.

### NanoBRET Target Engagement Assay in HEK293 Cells Transiently Transfected with BTK NanoLuc Fusion Vectors

The NanoBRET target engagement intracellular kinase assay was used to quantify the binding of compounds to wild-type BTK (performed at Reaction Biology Corp, Malvern, PA. USA). HEK293 cells expressing NanoLuc-BTK Fusion vector were plated in a 384-well plate as per 4000 per cell and were treated with the appropriate compound (starting at 10 and 1 μM, 10-dose with 3-fold dilution) and Ibrutinib (**1g**) as reference compound/positive control (starting at 10 μM, 10-dose with 3-fold dilution) for 1h. BTK and BTK (C481S) target engagement was measured by NanoBRET assay.^58,77,78^ Curve fits were performed only when % NanoBret signal at the highest concentration of compounds was less than 55%. Experiments were carried out in 384-well format, with ∼4000 cells/well.

### Annexin V/Propidium Iodide Staining

Cells were washed in phosphate-buffered saline and resuspended in 300 μL of annexin V staining buffer (10 mM HEPES HCl (pH 7.4), 140 mM NaCl, 2.5 mM CaCl_2_) supplemented with 2.5 μg/mL fluorescein isothiocyanate-labeled annexin V (kind gift from Dr Patrick Duriez, Protein Core Facility, University of Southampton) and 12.5 μM propidium iodide (Invitrogen). Cells were analyzed by flow cytometry (Canto II system, BD Biosciences) and the percentage of viable cells was determined by calculating the proportion of Annexin V-/PI-cells as a percentage of all cells (results for untreated cells at 72 h set to 100%).

### RNA-seq and Bioinformatics

TMD8 cells were cultured in the presence of compounds (1 μM) or DMSO for 8 h and total RNA was extracted using an RNeasy mini kit (Qiagen). PolyA libraries were sequenced at the Oxford Genomics Centre (Oxford, UK) using a NovaSeq 6000 (Ilumina). Fastq files were aligned against the hg38 reference genome using HISAT2 and initial data quality control was performed using FastQC.^79^ Counts matrices were produced using HTseq-count^80^ and exported for differential expression analysis in EdgeR.^81^ Transcriptomic data were fitted to multifactor GLM models and tested for differential expression using quasi-likelihood f-tests. Multiple testing corrections were performed using the Benjamini–Hochberg procedure. Pathway analysis was performed using Ingenuity Pathway Analysis (IPA) software (Qiagen) based on expression log ratios and comparisons between responses to compounds was performed using gene set enrichment analysis (GSEA; v4.2.3) using weighted enrichment statistic.^82,83^

### Statistics

For statistical comparisons of Ca^2+^ and immunoblots results (performed in Prism9; GraphPad Software Inc.), normal distribution of data was confirmed using Shapiro–Wilk’s tests and analyzed using 1-sample *t*-tests (two-tailed).

### Data Availability

RNA-seq data have been deposited in the ArrayExpress database at EMBL-EBI (www.ebi.ac.uk/arrayexpress) under accession number E-MTAB-14363.

### Plasma Stability Assay

Compound stock solutions were prepared by dissolving purified compound powders in DMSO to a concentration of 40 μM. Human plasma (Cambridge Bioscience, fresh whole human plasma collected in Vacutainers containing sodium heparin) was diluted to 50% using PBS (Gibco, ThermoFisher) pH 7.4 before use. Protocol: 97.5 μL of diluted plasma and 2.5 μL of the appropriate compound stock were mixed thoroughly (vortex). Final composition: 1 μM compound, 2.5% v/v DMSO content. The resulting mixture was equally divided into 2 separate samples (for t = 0 h and t = 3 h measurements). The t_0_ sample was immediately quenched with 300 μL of cold methanol saturated with reserpine (reference). The t = 3 h sample was placed in an incubator/shaker for 3 h at 37 °C followed by the methanol quench. The resulting samples were mixed thoroughly and centrifuged at 3000 rpm for 15 min before 200 μL of the supernatant was transferred into an LC-MS vial for analysis. Ultrahigh performance liquid chromatography was performed using a Waters, Acquity UPLC BEH C18 (50.0 mm × 2.10 mm 1.70 μm) column, using a gradient elution from 20% acetonitrile (0.2% formic acid) to 100% acetonitrile (0.2% formic acid) over 10 min at 0.6 mL/min. The standardized remaining concentration of the compound was determined from the ratio of its integrated UV-absorbance (254 nm) peak (area under peak, AUP) and that of the reserpine standard.
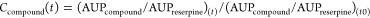


Calculations were performed in Excel using UPLC output files, and plots were generated in Prism9. Results are presented as mean ± s.d. from 3 independent results.

### Synthetic Chemistry

All air/moisture-sensitive reactions were carried out under an inert atmosphere (N_2_ or Ar), using oven or flame-dried glassware and using anhydrous solvents. All solvents and reagents were used as received from standard chemical suppliers unless otherwise stated. Reactions were monitored by thin layer chromatography (TLC) using indicated solvents on aluminum plates (0.25 mm), precoated with silica gel 60 with F254 indicator and visualized under UV light (254 nm) and/or by staining with KMnO_4_ followed by heating. Column chromatography was performed with Merck Kieselgel 60 silica gel or using Biotage Isolera One. Solvents were removed by rotary evaporator below 40 °C and the compounds were further dried using high vacuum pumps. Fourier transform infrared (FT-IR) spectra are reported in wavenumbers (cm^–1^) and were collected on a Nicolet 380 spectrometer fitted with a Diamond platform, as solids or neat liquids.

^1^H NMR and ^13^C NMR spectra were recorded on a Bruker Advance 400 spectrophotometer at 400 and 100 MHz, respectively. Chemical shifts (δ) are reported in ppm (parts per million) and referenced to residual solvent signals: ^1^H δ = 7.26 (CDCl_3_), 2.50 (d^6^-DMSO), 3.31 (CD_3_OD), ^13^C δ = 77.0 (CDCl_3_), 39.4 (d^6^-DMSO), 49.1 (CD_3_OD). Coupling constants (*J*) are reported in Hz and are rounded to the nearest 0.1 Hz. Melting points are uncorrected on an Electrothermal machine. Ultrahigh performance liquid chromatography was performed using a Waters, Acquity UPLC BEH C18 (50.0 mm × 2.1 mm 1.7 μm) column using gradient elution from 20% acetonitrile (0.2% formic acid) to 100% acetonitrile (0.2% formic acid) was performed in 5 min at 0.6 mL/min. High-resolution positive/negative ion electrospray ionization mass spectra were recorded. High-resolution mass spectrometry samples were analyzed using a MaXis (Bruker Daltonics, Bremen, Germany) time-of-flight (TOF) mass spectrometer. Samples were introduced to the mass spectrometer via a Dionex Ultimate 3000 autosampler and μHPLC pump.

#### General Procedure 1

The appropriate carboxylic acid derivative (1.0 equiv) was dissolved in anhydrous DMF (0.1 M) under an argon atmosphere, followed by triethylamine (1.5 equiv) and HATU (1.10 equiv). The reaction was left to stir at rt for 10 min before (*R*)-3-(4-phenoxyphenyl)-1-(piperidin-3-yl)-1H-pyrazolo[3,4-*d*]pyrimidin-4-amine (IbNH, **2**) was added to the mixture (1.00 equiv). The resulting reaction mixture was left to stir at rt for 16 h. It was then diluted with ethyl acetate and the organic phase was washed with sat. aq. NaHCO_3_ (1×) followed by brine (2×). The organic layer was dried (MgSO_4_) and concentrated *in vacuo* and the product was purified by flash column chromatography.

#### General Procedure 2

The appropriate 2-(methylthio)pyrimidine derivative (1.0 equiv) was dissolved in anhydrous DCM (0.1 M) at rt under an inert atmosphere, followed by the addition of di*tert*-butyl dicarbonate (3.0 equiv). The resulting mixture was left to stir at rt for 16 h. It was then diluted with ethyl acetate, and washed with sat. NH_4_Cl (1×) and brine (1×). The organic phase was dried (MgSO_4_) and concentrated *in vacuo*. The residue was then dissolved in anhydrous DCM (0.1 M) at rt under an inert atmosphere, followed by the addition of *m*-CPBA (2.2 equiv). The resulting mixture was left to stir for 2.5 h. The solution was diluted with EtOAc and washed with sat. aq. NaHCO_3_ (2×) and brine (1×). The organic layer was dried (MgSO_4_) and concentrated *in vacuo*. The resulting residue was dissolved in anhydrous DCM (0.1 M) at rt under an inert atmosphere. Trifluoroacetic acid (10 equiv) was added dropwise, and the reaction was left to stir for 2 h. The solution was then diluted with ethyl acetate and neutralized with sat. aq. NaHCO_3_. The organic layer was dried (MgSO_4_) and concentrated *in vacuo* and the product was then purified by flash column chromatography.

##### 2-(Methylthio)pyrimidine-4-carboxylic Acid (**3a**)

2-Chloropyrimidine-4-carboxylic acid (500 mg, 3.20 mmol, 1.0 equiv) was dissolved in methanol (30 mL). Sodium thiomethoxide (243 mg, 3.50 mmol, 1.1 equiv) and potassium carbonate (435 mg, 3.20 mmol, 1.0 equiv) were added to the stirred solution. The mixture was heated at 50 °C for 16 h, concentrated *in vacuo*, and then dissolved in a minimum amount of deionized water. The aqueous phase was acidified using acetic acid until pH ∼ 4. The aqueous phase was extracted with EtOAc (2×) followed by CH_2_Cl_2_ (1×). The combined organic layers were dried (MgSO_4_) and concentrated *in vacuo*. The crude was purified by flash column chromatography (6–10% MeOH in CH_2_Cl_2_ + 1% AcOH) to afford **3a** (310 mg, 58% yield) as a white solid. *R*_f_ 0.2 (10% MeOH in CH_2_Cl_2_); ^1^H NMR (400 MHz, DMSO-*d*_6_) δ ppm 8.87 (d, *J* = 5.0 Hz, 1H), 7.64 (d, *J* = 5.0 Hz, 1H), 2.56 (s, 3H); ^13^C NMR (101 MHz, DMSO-*d*_6_) δ ppm 172.6, 165.4, 160.4, 156.6, 116.4, 14.1; HRMS (ESI+) *m*/*z* calculated for C_6_H_7_N_2_O_2_S [M + H]^+^ 171.0223, found: 171.0222. Spectroscopic data were in accordance with the literature.^84^

##### 2-(Butylthio)pyrimidine-4-carboxylic Acid (**3b**)

The compound was synthesized from 2-chloropyrimidine-4-carboxylic acid (500 mg, 3.20 mmol, 1.0 equiv), which was dissolved in DMF (0.1 M) followed by the dropwise addition of 1-butanethiol (0.40 mL, 4.70 mmol, 1.5 equiv) and K_2_CO_3_ (435 mg, 3.20 mmol, 1.0 equiv). The reaction was left to stir at rt for 16 h. Once the reaction was complete the mixture was diluted with 300 mL of deionized water and extracted with EtOAc (5×). The combined organic layers were washed with brine, dried with MgSO_4_, and concentrated *in vacuo*. The crude was purified by flash column chromatography (10% MeOH in CH_2_Cl_2_ + 1% AcOH) to afford **3b** (58.0 mg, 9%) as a yellow oil. *R*_f_ 0.3 (10% MeOH in CH_2_Cl_2_ + 1% AcOH); FT-IR ν_max_/cm^–1^ (ATR) 1701, 1557, 1349, 1322, 1168, 863, 756, 665; ^1^H NMR (400 MHz, DMSO-*d*_6_) δ ppm 8.86 (d, *J* = 4.9 Hz, 1H), 7.63 (d, *J* = 4.9 Hz, 1H), 3.17 (t, *J* = 7.3 Hz, 2H), 1.63–1.70 (m, 2H), 1.42 (app. sxt, *J* = 7.5 Hz, 2H), 0.91 (t, *J* = 7.3 Hz, 3H); ^13^C NMR (101 MHz, DMSO-*d*_6_) δ ppm 172.5, 165.4, 160.4, 156.5, 116.4, 31.2, 30.3, 21.8, 13.9. HRMS (ESI+) *m*/*z* calculated for C_9_H_13_N_2_O_2_S [M + H]^+^ 213.0692, found: 213.0697.

##### 2-(Phenylthio)pyrimidine-4-carboxylic Acid (**3c**)

The compound was synthesized from 2-chloropyrimidine-4-carboxylic acid (500 mg, 3.20 mmol, 1.0 equiv), which was dissolved in DMF (0.1 M), followed by the addition of K_2_CO_3_ (654 mg, 4.73 mmol, 2.5 equiv) and dropwise addition of thiophenol (0.70 mL, 6.30 mmol, 2.5 equiv). When the reaction was complete, the mixture was diluted with deionized water, acidified with aq. 2 M HCl to ∼ pH 3, and extracted with EtOAc (5×). The combined organic layers were washed with brine, dried with MgSO_4_, and concentrated *in vacuo*. The crude was purified by flash column chromatography (20% acetone in CH_2_Cl_2_ + 1% AcOH) to afford **3c** (364 mg, 50%). *R*_f_ 0.2 (20% Acetone in CH_2_Cl_2_ + 1% AcOH); mp 84–85 °C; FT-IR ν_max_/cm^–1^ (ATR) 1560, 1474, 1284, 1232, 1201, 1166, 733, 702, 684; ^1^H NMR (400 MHz, DMSO-*d*_6_) δ ppm 8.80 (d, *J* = 4.9 Hz, 1H), 7.68 (d, *J* = 4.9 Hz, 1H), 7.60–7.66 (m, 2H), 7.43–7.55 (m, 3H); ^13^C NMR (101 MHz, DMSO-*d*_6_) δ ppm 172.1, 165.2, 160.8, 156.8, 135.4, 129.9 (2C), 129.0, 117.4. HRMS (ESI+) *m*/*z* calculated for C_11_H_9_N_2_O_2_S [M + H]^+^ 233.0379, found: 233.0385.

##### 2-(Methylthio)pyrimidine-5-carboxylic Acid (**3e**)

2-Chloropyrimidine-5-carboxylic acid (2.00 g, 12.6 mmol, 1.0 equiv) was dissolved in THF (60 mL) at rt under an inert atmosphere. Sodium thiomethoxide (0.97 g, 13.9 mmol, 1.1 equiv) and potassium carbonate (1.70 g, 12.6 mmol, 1.0 equiv) were added portion-wise to the stirred solution. The resulting mixture was heated at 60 °C for 16 h and then concentrated *in vacuo*. The residue was dissolved in a minimal amount of deionized water and the aqueous phase was acidified with glacial acetic acid until ∼ pH 4. The white precipitate was washed with 10 mL of cold deionized water and filtered using Buchner filtration. The crude was purified by flash column chromatography (10% MeOH in CH_2_Cl_2_) to afford **3e** (1.30 g, 59% yield) as a white solid. *R*_f_ 0.2 (10% MeOH in CH_2_Cl_2_); ^1^H NMR (400 MHz, DMSO-*d*_6_) δ ppm 9.00 (s, 2H), 2.57 (s, 3H); ^13^C NMR (101 MHz, DMSO-*d*_6_) δ ppm 176.3, 165.4, 158.6, 120.5, 14.3; HRMS (ESI+) *m*/*z* calculated for C_6_H_7_N_2_O_2_S [M + H]^+^ 171.0223, found: 171.0227. Spectroscopic data were in accordance with the literature.^85^

##### 2-(Butylthio)pyrimidine-5-carboxylic Acid (**3f**)

2-Chloropyrimidine-5-carboxylic acid (500 mg, 3.20 mmol, 1.0 equiv) was dissolved in DMF (0.1 M) followed by the dropwise addition of 1-butanethiol (0.40 mL, 4.70 mmol, 1.5 equiv) and K_2_CO_3_ (435 mg, 3.20 mmol, 1.0 equiv). The reaction was left to stir at rt for 16 h. Once the reaction was complete the mixture was diluted with 300 mL of deionized water and extracted with EtOAc (5×). The combined organic layers were washed with brine, dried with MgSO_4_, and concentrated *in vacuo*. The crude was purified by flash column chromatography (50% EtOAc in PE + 0.5% AcOH) to afford **3f** (259 mg, 39%) as a yellow oil. *R*_f_ 0.2 (50% EtOAc in PE + 0.5% AcOH); FT-IR ν_max_/cm^–1^ (ATR) 2952, 2926, 2868, 1671, 1577, 1530, 1384, 1284, 1250, 1203, 1187, 1133, 926, 831, 782, 728, 642; ^1^H NMR (400 MHz, DMSO-*d*_6_) δ ppm 13.58 (br. s, 1H), 8.99 (s, 2H), 3.17 (t, *J* = 7.3 Hz, 2H), 1.59–1.72 (m, 2H), 1.42 (app. sxt, *J* = 7.4 Hz, 2H), 0.90 (t, *J* = 7.4 Hz, 3H); ^13^C NMR (101 MHz, DMSO-*d*_6_) δ ppm 176.0, 165.4, 158.6, 120.5, 31.1, 30.5, 21.8, 13.9. HRMS (ESI+) *m*/*z* calculated for C_9_H_13_N_2_O_2_S [M + H]^+^ 213.0692, found: 213.0694.

##### 2-(Phenylthio)pyrimidine-5-carboxylic Acid (**3g**)

2-Chloropyirmidine-5-carboxylic acid (300 mg, 1.89 mmol, 1.0 equiv) was dissolved in DMF (0.1 M), followed by the addition of K_2_CO_3_ (654 mg, 4.73 mmol, 2.5 equiv) and dropwise addition of thiophenol (0.70 mL, 6.30 mmol, 2.5 equiv). When the reaction was complete, the mixture was diluted with deionized water, acidified with aq. 2 M HCl to ∼ pH 3, and extracted with EtOAc (5×). The combined organic layers were washed with brine, dried with MgSO_4_, and concentrated *in vacuo*. The crude was purified by flash column chromatography (20% Acetone in CH_2_Cl_2_ + 1% AcOH) to afford **3g** (168 mg, 38%) as a white solid. *R*_f_ 0.3 (20% Acetone in CH_2_Cl_2_ + 1% AcOH); mp decomposed at 188 °C; FT-IR ν_max_/cm^–1^ (ATR) 1626, 1571, 1362, 1199, 1140, 847, 797, 739, 704, 684, 640; ^1^H NMR (400 MHz, DMSO-d^6^) δ ppm 8.92 (br s, 1H), 8.80 (s, 2H), 7.59–7.63 (m, 2H), 7.45–7.49 (m, 3H); ^13^C NMR (101 MHz, DMSO-d^6^) δ ppm 171.1, 161.0, 158.8, 135.4, 132.5, 129.8, 129.7, 129.2. HRMS (ESI+) *m*/*z* calculated for C_11_H_9_N_2_O_2_S [M + H]^+^ 233.0379, found: 233.0385.

##### (*R*)-(3-(4-Amino-3-(4-phenoxyphenyl)-1H-pyrazolo[3,4-*d*]pyrimidin-1-yl)piperidin-1-yl)(2-(methylthio)pyrimidin-4-yl)methanone (**4a**)

The compound was synthesized using general procedure 1 from **3a** (200 mg, 1.20 mmol, 1.0 equiv). The final product was dried using MgSO_4_ and concentrated *in vacuo*. The crude was purified by flash column chromatography (30% acetone in CH_2_Cl_2_) to afford **4a** (362 mg, 57% yield) as a white solid. *R*_f_ 0.3 (30% acetone in CH_2_Cl_2_); mp 128–130 °C; FT-IR ν_max_/cm^–1^ (ATR) 1622, 1565, 1476, 1311, 1230, 1128, 751; ^1^H NMR showed two conformers in a 1:1 ratio. When resolvable and supported by 2D NMR, both signals associated with a proton are reported. ^1^H NMR (400 MHz, DMSO-*d*_6_) δ ppm 8.78/8.62 (d, *J* = 5.0 Hz, 1H), 8.29/8.16 (s, 1H), 7.60–7.66/7.66–7.72 (m, 2H), 7.40–7.48 (m, 2H), 7.13/7.37 (d, *J* = 5.0 Hz, 1H), 7.09–7.22 (m, 5H), 4.81–4.92 (m, 1H), 4.49–4.58 + 3.76–3.84 + 3.40–3.49 (m, 2H), 4.12–4.21 + 3.64–3.65 + 3.16–3.33 (m, 2H), 2.52/2.54 (s, 3H), 2.24–2.38 (m, 1H), 2.13–2.23 (m, 1H), 1.85–1.95/2.02–2.12 (m, 1H), 1.62–1.80 (m, 1H) ^13^C NMR (101 MHz, DMSO-*d*_6_) δ ppm 171.7/171.9, 165.1/165.2, 161.5/161.9, 159.3/159.6, 158.6/158.7, 157.6, 156.7/156.8, 156.2/155.9, 154.4/154.5, 143.7/143.9, 130.6, 130.5, 128.3/128.4, 124.3, 119.5, 119.4, 115.0/115.1, 97.8, 52.2/52.9, 45.9/50.6, 42.1/46.9, 29.3/29.9, 23.6/24.8, 14.0/14.1; HRMS for [M + H]^+^ calculated *m*/*z* 539.1972, found *m*/*z* 539.1986.

##### (R)-(3-(4-Amino-3-(4-phenoxyphenyl)-1H-pyrazolo[3,4-*d*]pyrimidin-1-yl)piperidin-1-yl)(2-(butylthio)pyrimidin-4-yl)methanone (**4b**)

The compound was synthesized from **3b** (50.0 mg, 0.24 mmol, 1.00 equiv) using general procedure 1. The final product was dried using MgSO_4_ and concentrated *in vacuo*. The crude was purified by flash column chromatography (30–50% acetone in CH_2_Cl_2_) to afford **4b** (94.0 mg, 42% yield) as a white solid. *R*_f_ 0.3 (40% acetone in CH_2_Cl_2_); mp 113–114 °C; FT-IR ν_max_/cm^–1^ (ATR) 1617, 1560, 1541, 1474, 1412, 1331, 1234, 1201, 1128, 840, 799, 740, 691, 642; ^1^H NMR showed two conformers in a 1:1 ratio. When resolvable and supported by 2D NMR, both signals associated with a proton are reported. ^1^H NMR (400 MHz, DMSO-*d*_6_) δ ppm 8.77/8.61 (d, *J* = 4.9 Hz, 1H), 8.29/8.15 (s, 1H), 7.66–7.72/7.60–7.66 (m, 2H), 7.38–7.47 (m, 2H), 7.11/7.35 (d, *J* = 4.9 Hz, 1H), 7.08–7.21 (m, 5H), 4.79–4.91 (m, 1H), 4.50–4.58 + 3.41–3.51/3.78–3.95 (m, 2H), 4.11–4.22 + 3.64–3.67/3.18–3.33 (m, 2H), 2.98–3.18 (m, 2H), 2.12–2.36 (m, 2H), 1.66–2.12 (m, 2H), 1.50–1.70 (m, 2H), 1.27–1.47 (m, 2H), 0.83/0.90 (t, *J* = 7.3 Hz, 3H); ^13^C NMR showed two conformers. When resolvable and supported by 2D NMR, both signals are provided. ^13^C NMR (101 MHz, DMSO-*d*_6_) δ ppm 171.6/171.3, 165.1, 162.0/161.5, 159.7/159.4, 158.7/158.6, 157.6, 156.8, 156.2/155.9, 154.5/154.4, 143.9/143.6, 130.6, 130.5, 128.4/128.3, 124.2, 119.4 (x2), 115.3/115.1, 97.9/97.8, 53.0/52.2, 50.5/45.8, 46.9/42.2, 31.0/31.2, 30.3/30.1, 29.4/29.3, 24.8/23.5, 21.9/21.8, 14.0/13.9. HRMS (ESI+) *m*/*z* calculated for C_31_H_32_N_8_O_2_S [M + H]^+^ is 581.2442, found: 581.2457.

##### (R)-(3-(4-Amino-3-(4-phenoxyphenyl)-1H-pyrazolo[3,4-*d*]pyrimidin-1-yl)piperidin-1-yl)(2-(phenylthio)pyrimidin-4-yl)methanone (**4c**)

The compound was synthesized from **3c** (90.0 mg, 0.39 mmol, 1.0 equiv) using general procedure 1. The final product was dried using MgSO_4_ and concentrated *in vacuo*. The crude was purified by flash column chromatography (30% acetone in CH_2_Cl_2_) to afford **4c** (167 mg, 71% yield) as a white solid. *R*_f_ 0.2 (30% acetone in CH_2_Cl_2_); mp 106–107 °C; FT-IR ν_max_/cm^–1^ (ATR) 1622, 1560, 1539, 1474, 1439, 1284, 1231, 1198, 842, 801, 748, 688; ^1^H NMR showed two conformers in a 1:1 ratio. When resolvable and supported by 2D NMR, both signals associated with a proton are reported. ^1^H NMR (400 MHz, DMSO-*d*_6_) δ ppm 8.76/8.58 (d, *J* = 5.0 Hz, 1H), 8.28/8.18 (s, 1H), 7.62–7.71 (m, 3H), 7.53–7.58 (m, 1H), 7.47–7.52 (m, 1H), 7.37–7.47 (m, 4H), 7.10–7.21 (m, 5H), 4.71–4.81 (m, 1H), 4.46–4.54 + 3.34–3.42/3.72–3.95 (m, 2H), 3.96–4.05/3.06–3.16 (m, 1H), 3.52–3.63/3.27–3.35 (m, 1H), 2.06–2.36 (m, 2H), 1.38–2.08 (m, 2H); ^13^C NMR showed two conformers. When resolvable and supported by 2D NMR, both signals are provided. ^13^C NMR (101 MHz, DMSO-*d*_6_) δ ppm 171.3/171.0, 164.9/164.8, 162.1/161.6, 160.1/159.8, 158.7/158.6, 157.6, 156.8, 156.2/155.8, 154.5/154.4, 143.9/143.6, 135.6, 130.6, 130.5, 130.0/129.9, 129.9/129.7, 129.0, 128.4, 124.3, 119.5, 119.4, 116.2, 98.0/97.8, 52.8/52.2, 46.8/42.3, 50.4/45.9, 29.8/28.9, 24.7/23.3; HRMS (ESI+) *m*/*z* calculated for C_33_H_28_N_8_O_2_S [M + H]^+^ is 601.2129, found: 601.2136.

##### (R)-(3-(4-Amino-3-(4-phenoxyphenyl)-1H-pyrazolo[3,4-*d*]pyrimidin-1-yl)piperidin-1-yl)(5-chloro-2-(methylthio)pyrimidin-4-yl)methanone (**4d**)

The compound was synthesized from commercially available 5-chloro-2-(methylthio)pyrimidine-4-carboxylic acid **(3d)** (106 mg, 0.52 mmol, 1.0 equiv) using general procedure 1. The final product was dried using MgSO_4_ and concentrated *in vacuo*. The crude was purified by flash column chromatography (30% acetone in CH_2_Cl_2_) to afford **4d** (235 mg, 75% yield) as a white solid. *R*_f_ 0.5 (30% Acetone in CH_2_Cl_2_); mp 170–172 °C; FT-IR ν_max_/cm^–1^ (ATR) 1623, 1564, 1475, 1389, 1230; ^1^H NMR showed two conformers in a 1:1 ratio. When resolvable and supported by 2D NMR, both signals associated with a proton are reported. ^1^H NMR (400 MHz, DMSO-*d*_6_) δ ppm 8.90/8.77 (s, 1H), 8.29/8.15 (s, 1H), 7.67–7.73/7.60–7.65 (m, 2H), 7.39–7.48 (m, 2H), 7.08–7.22 (m, 5H), 4.75–4.86 (m, 1H), 4.51–4.59 + 3.46–3.54/3.60–3.76 (m, 2H), 4.29–4.40/3.20–3.29 (m, 1H), 3.37–3.46/3.10–3.20 (m, 1H), 2.57/2.54 (s, 3H), 2.25–2.41 (m, 1H), 2.13–2.22 (m, 1H), 2.09–1.85/1.78–1.58 (m, 2H). ^13^C NMR showed two conformers. When resolvable and supported by 2D NMR, both signals are provided. ^13^C NMR (100 MHz, DMSO-*d*_6_) δ ppm 170.6, 162.6, 159.5/159.2, 158.7/158.6, 158.4/158.2, 157.6, 156.8, 156.2/155.9, 154.5/154.4, 143.9/143.7, 130.6, 130.5, 128.4/128.3, 124.2, 122.3, 119.4 (x2), 98.0/97.9, 53.1/52.3, 50.0/46.4, 45.3/41.5, 29.7/29.6, 25.0/24.0, 14.6/14.5. HRMS (ESI+) *m*/*z* calculated for C_28_H_25_ClN_8_O_2_S [M + H]^+^ is 573.1588, found: 573.1576.

##### (*R*)-(3-(4-Amino-3-(4-phenoxyphenyl)-1H-pyrazolo[3,4-*d*]pyrimidin-1-yl)piperidin-1-yl)(2-(methylthio)pyrimidin-5-yl)methanone (**4e**)

The compound was synthesized from **3e** (200 mg, 1.20 mmol, 1.0 equiv) using general procedure 1. The final product was dried using MgSO_4_ and concentrated *in vacuo*. The crude was purified by flash column chromatography (30% acetone in CH_2_Cl_2_) to afford **4e** (526 mg, 83% yield) as a white solid. *R*_f_ 0.4 (30% Acetone in CH_2_Cl_2_); mp 148–151 °C; FT-IR ν_max_/cm^–1^ (ATR) 1576, 1487, 1392, 1231, 1201; NMR showed two conformers in a 1:1 ratio. When resolvable and supported by 2D NMR, both signals associated with a proton are reported. ^1^H NMR (400 MHz, DMSO-*d*_6_) δ ppm 8.72/8.54 (br. s, 2H), 8.25/8.11 (br. s, 1H), 7.57–7.71 (m, 2H), 7.38–7.45 (m, 2H), 7.07–7.19 (m, 5H), 4.78–4.95 (m, 1H), 4.45–4.61 + 3.35–3.45/3.72–3.93 (m, 2H), 3.97–4.14 + 3.58–3.71/3.20–3.33 (m, 2H), 2.53 (s, 3H), 2.20–2.33 (m, 1H), 2.10–2.19 (m, 1H), 1.64–2.09 (m, 2H); ^13^C NMR showed two conformers. When resolvable and supported by 2D NMR, both signals are provided. ^13^C NMR (100 MHz, DMSO-*d*_6_) δ ppm 173.0, 165.3, 158.6, 157.6, 156.8, 154.4, 143.9/143.5, 130.7, 130.5, 128.4, 125.6/125.1, 124.2, 119.5, 119.4, 97.9, 52.7/52.1, 51.5/46.3, 47.8/42.4, 29.4/29.1, 24.7/23.1, 14.1; HRMS (ESI+) *m*/*z* calculated for C_28_H_27_N_8_O_2_S [M + H]^+^ is 539.1972, found: 539.1985.

##### (R)-(3-(4-Amino-3-(4-phenoxyphenyl)-1H-pyrazolo[3,4-*d*]pyrimidin-1-yl)piperidin-1-yl)(2-(butylthio)pyrimidin-5-yl)methanone (**4f**)

The compound was synthesized from **3f** (83.0 mg, 0.39 mmol, 1.0 equiv) using general procedure 1. The final product was dried using MgSO_4_ and concentrated *in vacuo*. The crude was purified by flash column chromatography (30% acetone in CH_2_Cl_2_) to afford **4f** (188 mg, 83% yield) as a white solid. *R*_f_ 0.3 (30% acetone in CH_2_Cl_2_); mp 63–64 °C; FT-IR ν_max_/cm^–1^ (ATR) 1623, 1565, 1519, 1487, 1390, 1230, 1165, 843, 801, 754, 692; NMR showed two conformers in a 1:1 ratio. When resolvable and supported by 2D NMR, both signals associated with a proton are reported. ^1^H NMR (400 MHz, DMSO-*d*_6_) δ ppm 8.70/8.56 (br. s, 2H), 8.26/8.12 (br. s, 1H), 7.56–7.69 (br. m, 2H), 7.38–7.45 (m, 2H), 7.05/7.20 (m, 5H), 4.81–4.94 (m, 1H), 3.20–4.57 (m, 4H), 3.02–3.18 (br. m, 2H), 2.11–2.33 (m, 2H), 1.67–2.07 (m, 2H), 1.55–1.69 (br. m, 2H), 1.34–1.45 (br. m, 2H), 0.83–0.94 (br. m, 3H); ^13^C NMR showed two conformers. When resolvable and supported by 2D NMR, both signals are provided. ^13^C NMR (101 MHz, DMSO-*d*_6_) δ ppm 172.7, 165.3, 158.6, 157.6, 156.8, 156.3, 156.0, 154.5, 143.8, 130.6, 130.5, 128.3, 124.2, 122.0, 119.5, 119.4, 97.8, 52.2, 47.2/41.6, 46.2/42.3, 31.2, 30.3, 29.9, 24.7/23.2, 21.8, 14.0; HRMS (ESI+) *m*/*z* calculated for C_31_H_32_N_8_O_2_S [M + H]^+^ is 581.2442, found: 581.2440.

##### (R)-(3-(4-Amino-3-(4-phenoxyphenyl)-1H-pyrazolo[3,4-*d*]pyrimidin-1-yl)piperidin-1-yl)(2-(phenylthio)pyrimidin-5-yl)methanone (**4g**)

The compound was synthesized from **3g** (90.0 mg, 0.39 mmol, 1.0 equiv) using general procedure 1. The final product was dried using MgSO_4_ and concentrated *in vacuo*. The crude was purified by flash column chromatography (30% acetone in CH_2_Cl_2_) to afford **4g** (135 mg, 57% yield) as a white solid. *R*_f_ 0.3 (30% acetone in CH_2_Cl_2_); mp 99–100 °C; FT-IR ν_max_/cm^–1^ (ATR) 1618, 1570, 1519, 1475, 1390, 1230, 1196, 1127, 846, 801, 752, 690; ^1^H NMR showed two conformers in a 1:1 ratio. When resolvable and supported by 2D NMR, both signals associated with a proton are reported. ^1^H NMR (400 MHz, DMSO-*d*_6_) δ ppm 8.71/8.57 (s, 2H), 8.27/8.13 (s, 1H), 7.54–7.75 (m, 4H), 7.38–7.54 (m, 5H), 7.08–7.22 (m, 5H), 4.82–4.94 (m, 1H), 4.46–4.64 + 3.34–3.44/3.71–3.95 (m, 2H), 4.02–4.18/3.21–3.31 (m, 1H), 3.55–3.73/3.28–3.32 (m, 1H), 2.21–2.36 (m, 1H), 2.11–2.23 (m, 1H), 1.64–2.06 (m, 2H); ^13^C NMR showed two conformers. When resolvable and supported by 2D NMR, both signals are provided. ^13^C NMR (101 MHz, DMSO-*d*_6_) δ ppm 172.5, 165.1, 158.7, 157.6, 156.8, 156.5, 156.0, 154.5, 143.6, 135.6, 130.6, 130.5, 130.0, 129.9, 128.8, 128.4, 124.2, 119.5, 119.4, 119.0, 97.9, 52.2, 46.2/42.5, 51.3/47.6, 29.6, 24.8/23.1; HRMS (ESI+) *m*/*z* calculated for C_33_H_28_N_8_O_2_S [M + H]^+^ is 601.2129, found: 601.2133

##### (*R*)-(3-(4-Amino-3-(4-phenoxyphenyl)-1H-pyrazolo[3,4-*d*]pyrimidin-1-yl)piperidin-1-yl)(2-(methylsulfonyl)pyrimidin-4-yl)methanone (**5a**)

The compound was synthesized from **4a** (200 mg, 0.40 mmol, 1.0 equiv) using general procedure 2. The crude was purified by flash column chromatography (20% acetone in CH_2_Cl_2_) to afford **5a** (105 mg, 74% yield) as a white solid. *R*_f_ 0.3 (30% acetone in CH_2_Cl_2_); mp 159–160 °C; FT-IR ν_max_/cm^–1^ (ATR) 2359, 1634, 1568, 1475, 1312, 1231, 1128; ^1^H NMR showed two conformers in a 1:1 ratio. When resolvable and supported by 2D NMR, both signals associated with a proton are reported. ^1^H NMR (400 MHz, DMSO-*d*_6_) δ ppm 9.26/9.12 (d, *J* = 5.0 Hz, 1H), 8.29/8.16 (s, 1H), 8.05/7.83 (d, *J* = 5.0 Hz, 2H), 7.67–7.73/7.58–7.65 (m, 2H), 7.39–7.48 (m, 2H), 7.08–7.22 (m, 5H), 4.82–4.99 (m, 1H), 4.53–4.64/3.72–3.83 (m, 1H), 4.22–4.31/3.29–3.34 (m, 1H), 3.33–3.93/3.47–3.56 (m, 1H), 3.60–3.69/3.21–3.29 (m, 1H), 3.48/3.46 (s, 3H), 2.25–2.41 (m, 1H), 2.13–2.22 (m, 1H), 1.70–2.09 (m, 2H). ^13^C NMR showed two conformers. When resolvable and supported by 2D NMR, both signals are provided. ^13^C NMR (100 MHz, DMSO-*d*_6_) δ ppm 165.4/165.3, 164.4/164.2, 162.8/162.3, 161.3/161.1, 158.7/158.6, 157.6, 156.8/156.7, 156.2/156.0, 154.5/154.3, 144.0/143.7, 130.6, 130.5, 122.9, 128.4/128.3, 124.2/124.3, 119.4, 119.5, 97.9/97.8, 52.8/52.2, 50.5/47.0, 46.0/42.2, 39.4, 29.9, 24.7/23.5; HRMS for [M + H]^+^ calculated *m*/*z* 571.1870 found *m*/*z* 571.1883.

##### (3-(4-Amino-3-(4-phenoxyphenyl)-1H-pyrazolo[3,4-*d*]pyrimidin-1-yl)piperidin-1-yl)(2-(butylsulfonyl)pyrimidin-4-yl)methanone (**5b**)

The compound was synthesized from **4b** (129 mg, 0.22 mmol, 1.0 equiv) using general procedure 2. The final product was dried using MgSO_4_ and concentrated *in vacuo*. The crude was purified by flash column chromatography (30% acetone in CH_2_Cl_2_) to afford **5b** (135 mg, 57% yield) as a white solid. *R*_f_ 0.2 (30% acetone in CH_2_Cl_2_); mp 111–112 °C; FT-IR ν_max_/cm^–1^ (ATR) 1623, 1560, 1519, 1487, 1475, 1310, 1275, 1230, 1165, 1126, 1099, 982, 943, 868, 842, 801, 753, 724, 691; ^1^H NMR showed two conformers in a 1:1 ratio. When resolvable and supported by 2D NMR, both signals associated with a proton are reported. ^1^H NMR (400 MHz, DMSO-*d*_6_) δ ppm 9.26/9.12 (d, *J* = 5.0 Hz, 1H), 8.29/8.12 (s, 1H), 8.05/7.82 (d, *J* = 5.0 Hz, 1H), 7.67–7.73/7.60–7.65 (m, 2H), 7.40–7.48 (m, 2H), 7.10–7.22 (m, 5H), 4.79–4.89/4.89–4.99 (m, 1H), 4.51–4.62 + 3.47–3.55/3.78–3.96 (m, 2H), 4.18–4.29 + 3.24–3.33/3.62–3.68 (m, 2H), 3.47–3.67 (m, 2H), 2.13–2.39 (m, 2H), 1.71–2.12 (m, 2H), 1.65–1.75/1.54–1.64 (m, 2H), 1.36–1.49/1.20–1.34 (m, 2H), 0.77/0.89 (t, *J* = 7.3 Hz, 3H); ^13^C NMR showed two conformers. When resolvable and supported by 2D NMR, both signals are provided. ^13^C NMR (101 MHz, DMSO-*d*_6_) δ ppm 164.9/164.6, 164.3/164.2, 162.9/162.4, 161.4/161.2, 158.7/158.6, 157.6, 156.8/156.7, 156.2/156.0, 154.5/154.4, 144.0/143.6, 130.6, 130.5, 128.4/128.3, 124.2, 123.0/122.9, 119.4 (x2), 97.9, 53.0/52.2, 50.7/50.5, 50.4/46.0, 47.0/42.3, 29.8/29.4, 24.1/24.0, 24.7/23.4, 21.5/21.4, 13.9/13.8. HRMS (ESI+) *m*/*z* calculated for C_31_H_32_N_8_O_4_S [M + H]^+^ is 613.2340, found: 613.2345.

##### (3-(4-Amino-3-(4-phenoxyphenyl)-1H-pyrazolo[3,4-*d*]pyrimidin-1-yl)piperidin-1-yl)(2-(phenylsulfonyl)pyrimidin-4-yl)methanone (**5c**)

The compound was synthesized from **4c** (125 mg, 0.21 mmol, 1.0 equiv) using general procedure 2. The final product was dried using MgSO_4_ and concentrated *in vacuo*. The crude was purified by flash column chromatography (30% acetone in CH_2_Cl_2_) to afford **5c** (39.0 mg, 29% yield) as a white solid. *R*_f_ 0.2 (30% acetone in CH_2_Cl_2_); mp 124–125 °C; FT-IR ν_max_/cm^–1^ (ATR) 1623, 1570, 1520, 1474, 1284, 1195, 1230, 1127, 855, 802, 753, 690, 595; ^1^H NMR showed two conformers in a 1:1 ratio. When resolvable and supported by 2D NMR, both signals associated with a proton are reported. ^1^H NMR (400 MHz, DMSO-*d*_6_) δ ppm 9.20/9.04 (d, *J* = 5.0 Hz, 1H), 8.29/8.01 (s, 1H), 8.02–8.07/7.89–7.95 (m, 2H), 7.99/7.72 (d, *J* = 5.0 Hz, 1H), 7.78–7.87/7.67–7.76 (m, 1H), 7.66–7.76/7.50–7.56 (m, 2H), 7.62–7.71 (m, 2H), 7.39–7.48 (m, 2H), 7.07–7.25 (m, 5H), 4.80–4.90/4.64–4.73 (m, 1H), 4.48–4.58 + 3.43–3.50/3.69–3.81 (m, 2H), 4.03–4.15/3.09–3.20 (m, 1H), 3.34–3.45 (m, 1H), 2.11–2.35 (m, 2H), 1.48–2.10 (m, 2H); ^13^C NMR showed two conformers. When resolvable and supported by 2D NMR, both signals are provided. ^13^C NMR (101 MHz, DMSO-*d*_6_) δ ppm 165.5, 164.0, 161.5/161.2, 158.7/158.6, 157.6, 156.8, 156.2–155.8, 154.5/154.3, 144.0/143.5, 135.2/135.0, 130.5, 130.1, 129.8, 129.6/129.5, 128.4, 124.3, 122.9, 119.5, 119.4, 97.9, 52.9/52.2; 47.1/46.9, 46.0/42.4, 29.5, 24.6/23.2; HRMS (ESI+) *m*/*z* calculated for C_33_H_28_N_8_O_4_S [M + H]^+^ is 633.2027, found: 633.2033.

##### (*R*)-(3-(4-Amino-3-(4-phenoxyphenyl)-1H-pyrazolo[3,4-*d*]pyrimidin-1-yl)piperidin-1-yl)(5-chloro-2-(methylsulfonyl)pyrimidin-4-yl)methanone (**5d**)

The compound was synthesized using general procedure 2 from **4d** (298 mg, 0.52 mmol, 1.0 equiv). The organic layer was dried using MgSO_4_ and concentrated *in vacuo*. The crude was purified by flash column chromatography (30% acetone in CH_2_Cl_2_) to afford **5d** (235 mg, 75% yield) as a white solid. *R*_f_ 0.5 (30% acetone in CH_2_Cl_2_); mp 170–172 °C; FT-IR ν_max_/cm^–1^ (ATR) 1623, 1564, 1519, 1475, 1389, 1230, 1201; ^1^H NMR showed two conformers in a 1:1 ratio. When resolvable and supported by 2D NMR, both signals associated with a proton are reported. ^1^H NMR (400 MHz, DMSO-*d*_6_) δ ppm 9.38/9.29 (s, 1H), 8.30/8.16 (s, 1H), 7.67–7.73/7.58–7.65 (m, 2H), 7.39–7.48 (m, 2H), 7.08–7.22 (m, 5H), 4.90–4.75 (m, 1H), 4.53–4.62/3.65–3.74 (m, 1H), 4.46–4.41/3.21–3.32 (m, 1H), 3.75–3.85/3.53–3.62 (m, 1H), 3.53–3.46/3.05–3.17 (m, 1H), 3.50/3.44 (s, 3H), 2.25–2.41 (m, 1H), 2.13–2.22 (m, 1H), 1.60–1.95 (m, 2H). ^13^C NMR showed two conformers. When resolvable and supported by 2D NMR, both signals are provided. ^13^C NMR (100 MHz, DMSO-*d*_6_) δ ppm 163.5, 161.7, 160 (x2), 158.7/158.6, 157.6, 156.8, 156.2/156.0, 154.5/154.4, 143.9/143.7, 130.6, 130.5, 130.0, 128.3/128.2, 124.3/124.2, 119.4, 119.5, 97.9, 53.0/52.3, 49.8/46.3, 45.5/41.7, 40.0/39.9, 29.7, 25.0/24.1; HRMS for C_28_H_25_ClN_8_O_4_S [M + H]^+^ calculated *m*/*z* 605.1481, found *m*/*z* 605.1482.

##### (*R*)-(3-(4-Amino-3-(4-phenoxyphenyl)-1H-pyrazolo[3,4-*d*]pyrimidin-1-yl)piperidin-1-yl)(2-(methylsulfonyl)pyrimidin-5-yl)methanone (**5e**)

The compound was synthesized from **4e** (300 mg, 0.56 mmol, 1.0 equiv) using general procedure 2. The organic layers were dried using MgSO_4_ and concentrated *in vacuo*. The crude was purified by flash column chromatography (40% acetone in CHCl_3_) to afford **5e** (106 mg, 17% yield) as a pale yellow solid. *R*_f_ 0.1 (30% Acetone in CH_2_Cl_2_); mp 137–138 °C; FT-IR ν_max_/cm^–1^ (ATR) 1622, 1564, 1475, 1231, 1131; ^1^H NMR showed two conformers in a ca. 1:1 ratio. When resolvable and supported by 2D NMR, both signals associated with a proton are reported. ^1^H NMR (400 MHz, CDCl_3_) δ ppm 8.82/8.91 (s, 2H), 8.17/8.28 (s, 1H), 7.50–7.61 (m, 2H), 7.26–7.38 (m, 2H), 6.97–7.154 (m, 5H), 5.30–6.30 (br. s, 2H), 4.72–4.89/4.89–5.08 (m, 1H), 4.18–4.35 + 4.50–4.67 + 3.67–3.92 + 3.48–3.60 + 3.20–3.40 (m, 4H), 3.25/3.31 (s, 3H), 1.58–2.49 (m, 4H). ^13^C NMR (101 MHz, CDCl_3_) δ ppm 166.0/166.1, 163.6/163.8, 158.1, 156.9/157.0, 156.2, 155.9, 154.2, 131.9/132.2, 130.0, 129.9, 127.3/127.5, 119.5, 119.1, 98.6, 51.6/52.8, 48.0/51.6, 42.8/46.4, 39.2/39.3, 29.3/29.9, 23.1/24.7; HRMS for [M + H]^+^ calculated *m*/*z* 571.1870, found *m*/*z* 571.1885.

##### (R)-(3-(4-Amino-3-(4-phenoxyphenyl)-1H-pyrazolo[3,4-*d*]pyrimidin-1-yl)piperidin-1-yl)(2-(butylsulfonyl)pyrimidin-5-yl)methanone (**5f**)

The compound was synthesized from **4f** (147 mg, 0.25 mmol, 1.0 equiv) using general procedure 2. The final product was dried using MgSO_4_ and concentrated *in vacuo*. The crude was purified by flash column chromatography (30% acetone in CH_2_Cl_2_) to afford **5f** (63.0 mg, 41% yield) as a white solid. *R*_f_ 0.2 (30% acetone in CH_2_Cl_2_); mp 100–101 °C; FT-IR ν_max_/cm^–1^ (ATR) 1623, 1565, 1519, 1488, 1392, 1231, 844, 801, 754, 692; ^1^H NMR showed two conformers in a 1:1 ratio. When resolvable and supported by 2D NMR, both signals associated with a proton are reported. ^1^H NMR (400 MHz, DMSO-*d*_6_) δ ppm 9.21/9.11 (s, 2H), 8.31/8.16 (s, 1H), 7.66–7.75/7.59–7.66 (m, 2H), 7.38–7.50 (m, 2H), 7.09–7.26 (m, 5H), 4.88–5.03 (m, 1H), 4.65–4.20/3.14–3.95 (m, 7H), 2.22–2.36 (m, 1H), 2.15–2.23 (m, 1H), 1.76–2.06 (m, 2H), 1.61–1.75 (m, 2H), 1.32–1.50 (m, 2H), 0.78–0.97 (m, 3H); ^13^C NMR showed two conformers. When resolvable and supported by 2D NMR, both signals are provided.^13^C NMR (101 MHz, DMSO-*d*_6_) δ ppm 165.3, 163.9, 158.3, 157.7, 157.4, 156.7, 155.5, 154.3, 144.2/143.8, 132.9/132.7, 130.6, 130.5, 128.2, 124.3, 119.5, 119.4, 97.8, 52.7/52.1, 51.2/47.6, 50.7/50.6, 46.1/42.3, 29.7, 24.6/23.3, 24.1, 21.4, 13.9. HRMS (ESI+) *m*/*z* calculated for C_31_H_32_N_8_O_4_S [M + H]^+^ is 613.2340, found: 613.2334.

##### (R)-(3-(4-Amino-3-(4-phenoxyphenyl)-1H-pyrazolo[3,4-*d*]pyrimidin-1-yl)piperidin-1-yl)(2-(phenylsulfonyl)pyrimidin-5-yl)methanone (**5g**)

The compound was synthesized from **4g** (148 mg, 0.25 mmol, 1.0 equiv) using general procedure 2. The final product was dried using MgSO_4_ and concentrated *in vacuo*. The crude was purified by flash column chromatography (30% acetone in CH_2_Cl_2_) to afford **5g** (54 mg, 34% yield) as a white solid. *R*_f_ 0.2 (30% acetone in CH_2_Cl_2_); mp 121–122 °C; FT-IR ν_max_/cm^–1^ (ATR) 1623, 1565, 1520, 1475, 1231, 1127, 802, 754, 721, 687, 604, 557; ^1^H NMR showed two conformers in a 1:1 ratio. When resolvable and supported by 2D NMR, both signals associated with a proton are reported. ^1^H NMR (400 MHz, DMSO-*d*_6_) δ ppm 9.15/9.05 (s, 2H), 8.28/8.06 (s, 1H), 7.95–8.07 (m, 2H), 7.57–7.87 (m, 5H), 7.38–7.50 (m, 2H), 7.07–7.25 (m, 5H), 4.82–5.01 (m, 1H), 4.53–4.64/3.67–3.78 (m, 1H), 4.13–4.34/3.24–3.30 (m, 1H), 3.82–3.92/3.38–3.47(m, 1H), 3.54–3.65/3.16–3.26 (m, 1H), 2.23–2.34 (m, 1H), 2.11–2.23 (m, 1H), 1.67–2.04 (m, 2H); ^13^C NMR showed two conformers. When resolvable and supported by 2D NMR, both signals are provided. ^13^C NMR (101 MHz, DMSO-*d*_6_) δ ppm 166.0, 163.8, 158.7, 157.6, 156.8, 156.2, 155.9, 154.5, 144.0/143.6, 137.4/137.3, 135.2, 132.7/132.4, 130.6, 130.5, 130.1, 129.7, 128.3, 124.3, 119.5 (x2), 97.9, 52.7/52.1, 46.1/42.4, 51.1/47.6, 29.7, 24.6/23.3; HRMS (ESI+) *m*/*z* calculated for C_33_H_28_N_8_O_4_S [M + H]^+^ is 633.2027, found: 633.2031.

##### 4-Chloro-5-methyl-2-(methylthio)pyrimidine (**6a**)

2,4-Dichloro-5methylpyrimdine (200 mg, 1.20 mmol, 1.0 equiv) was dissolved in THF (0.1 M), followed by the addition of sodium thiomethoxide (95.0 mg, 1.40 mmol, 1.0 equiv). The reaction was left to stir at rt for 16 h. Once the reaction was complete, the solvent was evaporated *in vacuo*. The solid residue was then redissolved in deionized water and the aqueous phase was extracted with EtOAc (5×). The organic layers were combined, washed with brine, dried using MgSO_4_ and concentrated *in vacuo*. The crude was purified by flash column chromatography (0–30% EA in hexane) to afford **6a** (176 mg, 82%) as a white solid. *R*_f_ 0.2 (5% EtOAc in hexane); mp 74–75 °C; FT-IR ν_max_/cm^–1^ (ATR) 1557, 1514, 1363, 1321, 1242, 1211, 1169, 1106, 862, 756; ^1^H NMR (400 MHz, DMSO-*d*_6_) δ ppm 8.28 (s, 1H), 2.56 (s, 3H), 2.14 (s, 3H); ^13^C NMR (101 MHz, DMSO-*d*_6_) δ ppm 172.6, 157.6, 156.4, 127.5, 14.6, 12.8. HRMS (ESI+) *m*/*z* calculated for C_6_H_8_ClN_2_S [M + H]^+^ 175.0091, found: 175.0092.

##### 5-Bromo-4-methyl-2-(methylthio)pyrimidine (**6e**)

5-Bromo-4-methyl-2-chloropyrimidine (70 mg, 0.34 mmol, 1.0 equiv) was dissolved in THF (0.1 M). Sodium thiomethoxide (36 mg, 0.61 mmol, 1.5 equiv) was added to the stirred solution. The reaction was left to stir at rt for 16 h. Once the reaction was complete, the solvent was evaporated by rotary evaporation. The solid residue was then redissolved in deionized water and the aqueous phase was extracted with EtOAc (3×). The organic layers were combined and washed with brine, dried using MgSO_4_, and concentrated *in vacuo*. The crude was purified by flash column chromatography (0–10% EA in hexane) to afford **6e** (44 mg, 63%) as a colorless oil. *R*_f_ 0.5 (10% EtOAc in hexane); FT-IR ν_max_/cm^–1^ (ATR) 1546, 1514, 1394, 1304, 1202, 1029, 758, 559; ^1^H NMR (400 MHz, CDCl_3_) δ ppm 8.40 (s, 1H), 2.56 (s, 3H), 2.54 (s, 3H); ^13^C NMR (101 MHz, CDCl_3_) δ ppm 170, 165, 157, 115, 24, 14. HRMS (ESI+) *m*/*z* calculated for C_6_H_8_BrN_2_S [M + H]^+^ 218.9586, found: 218.9586.

##### 1-(1-(5-Methyl-2-(methylthio)pyrimidin-4-yl)piperidin-3-yl)-3-(4-phenoxyphenyl)-1H-pyrazolo[3,4-*d*]pyrimidin-4-amine (**7a**)

The compound was synthesized from **6a** (81.0 mg, 0.50 mmol, 1.0 equiv), which was dissolved in DMF (0.1 M). IbNH **2** (161 mg, 0.50 mmol, 1.0 equiv) was added to the mixture and stirred until fully dissolved before K_2_CO_3_ (130 mg, 0.90 mmol, 2.0 equiv) was added portion-wise. The reaction was left to stir at 80 °C for 24 h. Deionized water was added to the mixture before extraction with EtOAc (3×). The combined organic layers were washed with brine, dried with MgSO_4_, and concentrated *in vacuo*. The crude was purified using a Biotage Isolera One (5–60% EtOAc in CH_2_Cl_2_) to afford **7a** (82.0 mg, 30%) as a white solid. *R*_f_ 0.3 (30% EtOAc in CH_2_Cl_2_); mp 99–100 °C; FT-IR ν_max_/cm^–1^ (ATR) 1623, 1570, 1520, 1475, 1230, 1127, 852, 801, 753, 690, 603; ^1^H NMR (400 MHz, DMSO-*d*_6_) δ ppm 8.25 (s, 1H), 7.87 (s, 1H), 7.57–7.72 (m, 2H), 7.37–7.49 (m, 2H), 7.04–7.25 (m, 5H), 4.66–4.86 (m, 2H), 4.45–4.67 (m, 1H), 3.39–3.51 (m, 1H), 3.03–3.17 (m, 1H), 2.26–2.45 (m, 4H), 2.04–2.19 (m, 1H), 1.86–2.03 (m, 4H), 1.54–1.69 (m, 1H); ^13^C NMR (101 MHz, DMSO-*d*_6_) δ ppm 168.5, 159.9, 158.7, 157.5, 156.8, 156.0, 155.3, 154.4, 143.5, 130.6, 130.5, 128.5, 124.2, 119.4 (x2), 115.7, 97.9, 52.3, 48.7, 44.2, 30.0, 24.0, 14.1, 12.0. HRMS (ESI+) *m*/*z* calculated for C_28_H_28_N_8_OS [M + H]^+^ is 524.2180, found: 524.2184.

##### Methyl (R)-4-(3-(4-Amino-3-(4-phenoxyphenyl)-1H-pyrazolo[3,4-*d*]pyrimidin-1-yl)piperidin-1-yl)-2-(methylthio)pyrimidine-5-carboxylate (**7b**)

The compound was synthesized from methyl 2,4-dichloropyrimidine-5-carboxylate (107 mg, 0.52 mmol, 1.0 equiv), which was reacted with IbNH **2** (200 mg, 0.52 mmol, 1.0 equiv) and Et_3_N (0.11 mL, 0.78 mmol, 1.5 equiv) in DMF (0.1 M). The reaction was left to stir at rt for 16 h and then sodium thiomethoxide (42.0 mg, 0.59 mmol, 1.1 equiv) was added and was left to stir for another 16 h. To the reaction mixture was added water and it was extracted with EtOAc (3×). The combined organic layers were washed with brine (1×), dried using MgSO_4_ and concentrated *in vacuo*. The crude was purified by flash column chromatography (20% acetone in CH_2_Cl_2_) to afford **7b** (122 mg, 41%) as a white powder. *R*_f_ 0.2 (20% acetone in CH_2_Cl_2_); mp 100–101 °C; FT-IR ν_max_/cm^–1^ (ATR) 1623, 1560, 1520, 1474, 1388, 1308, 1284, 1231, 1164, 1128, 949, 855, 801, 753, 691, 604; ^1^H NMR (400 MHz, DMSO-*d*_6_) δ ppm 8.40 (s, 1H), 8.26 (s, 1H), 7.59–7.66 (m, 2H), 7.40–7.47 (m, 1H), 7.09–7.22 (m, 5H), 4.85–4.95 (m, 1H), 4.33–4.43 (m, 1H), 3.78 (s, 3H), 3.68–3.80 (m, 1H), 3.55–3.63 (m, 1H), 3.33–3.39 (m, 1H), 2.43 (s, 3H), 2.27–2.39 (m, 1H), 2.10–2.18 (m, 1H), 1.93–2.03 (m, 1H), 1.61–1.76 (m, 1H); ^13^C NMR (101 MHz, DMSO-*d*_6_) δ ppm 173.0, 166.4, 160.0, 159.6, 158.7, 157.6, 156.8, 156.1, 154.5, 143.7, 130.6, 130.5, 128.4, 124.2, 119.4 (x2), 105.5, 97.9, 52.6, 52.3, 51.1, 48.6, 29.4, 24.1, 14.0. HRMS (ESI+) *m*/*z* calculated for C_29_H_28_N_8_O_3_S [M + H]^+^ is 569.2078, found: 633.2085.

##### Ethyl (R)-4-(3-(4-Amino-3-(4-phenoxyphenyl)-1H-pyrazolo[3,4-*d*]pyrimidin-1-yl)piperidin-1-yl)-2-(methylthio)pyrimidine-5-carboxylate (**7c**)

The compound was synthesized from ethyl 4-chloro-2-(methylthio)pyrimidine-5-carboxylate (301 mg, 1.29 mmol, 1.0 equiv), IbNH **2** (500 mg, 1.29 mmol, 1.0 equiv), and Et_3_N (0.27 mL, 1.94 mmol, 1.5 equiv) in DMF (0.1 M). The mixture was stirred at rt for 16 h after it was worked up with water and extracted with EtOAc (3×). The combined organic layers were washed with brine, dried using MgSO_4_ and concentrated *in vacuo*. The resulting product was purified by Biotage Isolera One (0–50% EtOAc in CH_2_Cl_2_) to yield **7c** as a white powder (587 mg, 78%). *R*_f_ 0.3 (30% EtOAc in CH_2_Cl_2_); mp 101–102 °C; FT-IR ν_max_/cm^–1^ (ATR) 1626, 1558, 1519, 1487, 1386, 1361, 1231, 1163, 969, 844, 801, 787, 753, 692; ^1^H NMR (DMSO-*d*_6_, 500 MHz): δ (ppm) 8.40 (s, 1H), 8.26 (s, 1H), 7.58–7.63 (m, 2H), 7.41–7.47 (m, 2H), 7.10–7.23 (m, 5H), 4.86–4.95 (m, 1H), 4.31–4.41 (m, 1H), 4.18–4.28 (m, 2H), 3.72–3.80 (m, 1H), 3.56–3.65 (m, 1H), 3.32–3.42 (m, 1H), 2.44 (s, 3H), 2.28–2.37 (m, 1H), 2.12–2.19 (m, 1H), 1.95–2.04 (m, 1H), 1.63–1.75 (m, 1H), 1.24 (t, *J* = 7.1 Hz, 3H); ^13^C NMR (DMSO-*d*_6_, 101 MHz): δ (ppm) 172.9, 166.0, 159.9, 159.6, 158.7, 157.6, 156.8, 156.1, 154.5, 143.7, 130.6, 130.5, 128.4, 124.3, 119.4 (x2), 105.8, 97.9, 61.4, 52.3, 51.1, 48.5, 29.3, 24.1, 14.4, 14.0; HRMS (ESI+) *m*/*z* calculated for C_30_H_30_N_8_O_3_S [M + H]^+^ is 583.2234, found: 583.2241.

##### 4-(3-(4-Amino-3-(4-phenoxyphenyl)-1H-pyrazolo[3,4-*d*]pyrimidin-1-yl)piperidin-1-yl)-*N*-methyl-2-(methylthio)pyrimidine-5-carboxamide (**7d**)

The compound was synthesized using a 2 step procedure. In step 1 **7b** (300 mg, 0.53 mmol, 1.0 equiv) was dissolved in THF (0.1 M) and treated with 10% NaOH (1.2 mL) at reflux for 16 h. Once the reaction was complete, it was left to cool to rt and 2 M HCl was added to reach pH ∼ 3. The final product was obtained as a white powder by filtration (238 mg, 82%). The carboxylic acid product (100 mg, 0.18 mmol, 1.0 equiv) was treated with HATU (75 mg, 0.20 mmol, 1.1 equiv) and Et_3_N (0.040 mL, 0.27 mmol, 1.5 equiv) in DMF (0.1M). The mixture was left to stir at rt for 10 min before MeNH_2_ (0.07 mL, 0.54 mmol, 3.0 equiv) was added and left to stir at rt for a further 16 h. The mixture was diluted with deionized water and extracted with EtOAc (3×). The combined organic layers were washed with brine, dried using MgSO_4_ and concentrated *in vacuo*. The crude was purified by Biotage Isolera One (1–20% MeOH in CH_2_Cl_2_) to afford **7d** (95 mg, 93% yield) as a white solid. *R*_f_ 0.2 (5% MeOH in CH_2_Cl_2_); mp 141–142 °C; FT-IR ν_max_/cm^–1^ (ATR)) 1623, 1559, 1517, 1487, 1387, 1359, 1229, 1164, 1117, 968, 843, 801, 753, 691; ^1^H NMR (500 MHz, acetone-*d*_6_): δ (ppm) 8.13 (s, 1H), 8.03 (s, 1H), 7.57–7.62 (m, 2H), 7.50 (br q, *J* = 4.6 Hz, 1H), 7.27–7.33 (m, 2H), 7.02–7.07 (m, 3H), 6.97–7.00 (m, 2H), 4.88–4.95 (m, 1H), 4.29–4.35 (m, 1H), 3.81–3.87 (m, 1H), 3.44–3.51 (m, 1H), 3.05–3.15 (m, 1H), 2.69 (d, *J* = 4.6 Hz, 3H), 2.32 (s, 3H), 2.23–2.31 (m, 1H), 2.03–2.14 (m, 1H), 1.83–1.92 (m, 1H), 1.64–1.76 (m, 1H); ^13^C NMR (101 MHz, DMSO-*d*_6_): δ (ppm) 171.5, 166.8, 159.9, 158.5, 158.0, 156.8, 156.6, 155.8, 154.6, 143.6, 130.1, 130.0, 128.5, 123.8, 119.2, 119.0, 112.2, 98.0, 52.1, 50.5, 48.1, 29.7, 25.7, 23.8, 13.1; HRMS (ESI+) *m*/*z* calculated for C_29_H_29_N_9_O_2_S [M + H]^+^ is 568.2238, found: 568.2238.

##### 1-(1-(4-Methyl-2-(methylthio)pyrimidin-5-yl)piperidin-3-yl)-3-(4-phenoxyphenyl)-1H-pyrazolo[3,4-*d*]pyrimidin-4-amine (**7e**)

The compound was synthesized by mixing **6e** (57 mg, 0.26 mmol, 1.0 equiv), tris(dibenzylideneacetone) dipalladium(0) (24 mg, 0.03 mmol, 0.1 equiv), IbNH **2** (100 mg, 0.26 mmol, 1.0 equiv), XantPhos (30 mg, 0.05 mmol, 0.2 equiv), and NaO^t^Bu (52 mg, 0.54 mmol, 2.1 equiv) in a microwave vial. Anhydrous toluene (3 mL) was added to the vial under an inert atmosphere and left to stir at 100 °C for 16 h. Once the reaction is complete, it was poured over cold deionized water and extracted with EtOAc (2×). The organic layers were combined and washed with brine, dried using MgSO_4_ and concentrated *in vacuo*. The crude was purified using Biotage Isolera One (0–15% MeOH in CH_2_Cl_2_) to yield a white powder (105 mg, 77%). *R*_f_ 0.2 (5% MeOH in CH_2_Cl_2_); mp 102–103 °C; FT-IR ν_max_/cm^–1^ (ATR) 1588, 1557, 1472, 1407, 1229, 753, 692; ^1^H NMR (400 MHz, DMSO-*d*_6_) δ ppm 8.56 (s, 1H), 8.31 (br s, 1H), 7.71–7.89 (m, 2H), 7.41–7.48 (m, 2H), 7.15–7.23 (m, 1H), 7.07–7.15 (m, 4H), 4.70–4.79 (m, 1H), 3.09–3.16 (m 1H), 2.86–3.05 (m, 2H), 2.49–2.52 (m, 4H), 2.34 (s, 3H), 2.02–2.23 (m, 2H), 1.71–1.84 (m, 1H), 1.52–1.66 (m, 1H); ^13^C NMR (101 MHz, DMSO-*d*_6_) δ ppm 167.2, 164.1, 161.2. 157.7, 156.6, 156.0, 155.3, 154.3, 143.3, 130.7, 130.6, 129.2, 128.4, 124.3, 119.6, 119.0, 98.8, 54.6, 51.3, 45.9, 30.8, 26.5, 21.2, 14.2; HRMS (ESI+) *m*/*z* calculated for C_28_H_28_N_8_OS [M + H]^+^ is 525.2180, found: 525.2182.

##### 1-(1-(5-Methyl-2-(methylsulfonyl)pyrimidin-4-yl)piperidin-3-yl)-3-(4-phenoxyphenyl)-1H-pyrazolo[3,4-*d*]pyrimidin-4-amine (**8a**)

The compound was synthesized from **7a** (82 mg, 0.15 mmol, 1.0 equiv) using general procedure 2. The final product was dried using MgSO_4_ and concentrated *in vacuo*. The crude was purified by Biotage Isolera One (10–80% EtOAc in CH_2_Cl_2_) to afford **8a** (65 mg, 46% yield) as a white solid. *R*_f_ 0.2 (30% EtOAc in CH_2_Cl_2_); mp 125–126 °C; FT-IR ν_max_/cm^–1^ (ATR) 1623, 1587, 1568, 1516, 1488, 1355, 1302, 1232, 1140, 1091, 978, 951, 846, 801, 756, 691; ^1^H NMR (400 MHz, DMSO-*d*_6_) δ (ppm) 8.50 (s, 1H), 8.25 (s, 1H), 7.53–7.69 (m, 2H), 7.42–7.47 (m, 2H), 7.08–7.24 (m, 5H), 4.72–4.91 (m, 1H), 4.59–4.68 (m, 1H), 4.33–4.49 (m, 1H), 3.52–3.73 (m, 1H), 3.30 (s, 3H), 2.34–2.44 (m, 1H), 2.31 (s, 3H), 2.14–2.24 (m, 1H), 1.93–2.07 (m, 1H), 1.55–1.74 (m, 1H); ^13^C NMR (101 MHz, DMSO-*d*_6_) δ ppm 164.1, 163.4, 159.5, 158.7, 157.5, 156.8, 156.1, 154.4, 143.6, 130.6, 130.5, 128.4, 124.2, 119.4 (x2), 113.4, 97.9, 52.0, 48.6, 44.4, 39.4, 29.7, 23.7, 13.2; HRMS (ESI+) *m*/*z* calculated for C_28_H_28_N_8_O_3_S [M + H]^+^ is 557.2078, found: 557.2087.

##### Methyl 4-(3-(4-Amino-3-(4-phenoxyphenyl)-1H-pyrazolo[3,4-*d*]pyrimidin-1-yl)piperidin-1-yl)-2-(methylsulfonyl)pyrimidine-5-carboxylate (**8b**)

The compound was synthesized from **7b** (117 mg, 0.21 mmol, 1.0 equiv) using general procedure 2. The final product was dried using MgSO_4_ and concentrated *in vacuo*. The crude was purified by flash column chromatography (30% acetone in CH_2_Cl_2_) to afford **8b** (19.0 mg, 15% yield) as a white solid. *R*_f_ 0.2 (30% acetone in CH_2_Cl_2_); mp 128–129 °C; FT-IR ν_max_/cm^–1^ (ATR)) 1716, 1623, 1569, 1540, 1488, 1474, 1318, 1288, 1232, 1127, 1076, 950, 842, 792, 751, 692; ^1^H NMR (400 MHz, DMSO-*d*_6_) δ (ppm) 8.68 (s, 1H), 8.26 (s, 1H), 7.56–7.63 (m, 2H), 7.42–7.47 (m, 2H), 7.10–7.22 (m, 5H), 4.86–5.02 (m, 1H), 4.31–4.51 (br. m, 1H), 3.84 (s, 3H), 3.73–3.83 (br. m, 1H), 3.65–3.76 (m, 1H), 3.43–59 (m, 1H), 3.34 (s, 3H), 2.29–2.42 (m, 1H), 2.15–2.21 (m, 1H), 1.95–2.04 (m, 1H), 1.70–1.81 (m, 1H); ^13^C NMR (101 MHz, DMSO-*d*_6_) δ ppm 165.7, 165.5, 160.3, 160.1, 158.7, 157.5, 156.8, 156.1, 154.4, 143.8, 130.6, 130.5, 128.3, 124.3, 119.4 (x2), 111.7, 97.9, 53.3, 52.2, 51.1, 48.5, 39.2, 29.1, 23.6; HRMS (ESI+) *m*/*z* calculated for C_33_H_28_N_8_O_4_S [M + H]^+^ is 601.1976, found: 601.1982.

##### Ethyl (R)-4-(3-(4-Amino-3-(4-phenoxyphenyl)-1H-pyrazolo[3,4-*d*]pyrimidin-1-yl)piperidin-1-yl)-2-(methylsulfonyl)pyrimidine-5-carboxylate (**8c**)

The compound was synthesized from **7c** (100 mg, 0.17 mmol, 1.0 equiv) using general procedure 2. The final product was dried using MgSO_4_ and concentrated *in vacuo*. The crude was purified by Biotage Isolera One (1–15% MeOH in CH_2_Cl_2_) to afford **8c** (36 mg, 34% yield) as a white solid. *R*_f_ 0.2 (5% MeOH in CH_2_Cl_2_); mp 116–117 °C; FT-IR ν_max_/cm^–1^ (ATR) 1716, 1617, 1569, 1540, 1488, 1474, 1313, 1289, 1232, 1127, 1074, 951, 847, 793, 752, 692; ^1^H NMR (DMSO-*d*_6_, 500 MHz): δ (ppm) 8.67 (s, 1H), 8.26 (s, 1H), 7.56–7.63 (m, 2H), 7.42–7.47 (m, 2H), 7.10–7.22 (m, 5H), 4.89–4.98 (m, 1H), 4.20–4.50 (m, 3H), 3.77–3.88 (br. m, 1H), 3.63–3.75 (m, 1H), 3.47–3–59 (m, 1H), 3.35 (s, 3H), 2.29–2.42 (m, 1H), 2.15–2.21 (m, 1H), 1.95–2.04 (m, 1H), 1.70–1.81 (m, 1H), 1.26 (t, *J* = 7.1 Hz, 3H); ^13^C NMR (DMSO-*d*_6_, 101 MHz): δ (ppm) 165.5, 165.3, 160.1, 160.0, 158.7, 157.6, 156.8, 156.1, 154.4, 143.8, 130.6, 130.5, 128.3, 124.3, 119.4 (x2), 112.0, 97.9, 62.2, 52.2, 51.1, 48.4, 39.2, 29.1, 23.7, 14.3; HRMS (ESI+) *m*/*z* calculated for C_30_H_30_N_8_O_5_S [M + H]^+^ is 615.2133, found: 615.2138.

##### 4-(3-(4-Amino-3-(4-phenoxyphenyl)-1H-pyrazolo[3,4-*d*]pyrimidin-1-yl)piperidin-1-yl)-*N*-methyl-2-(methylsulfonyl)pyrimidine-5-carboxamide (**8d**)

The compound was synthesized from **7d** (95 mg, 0.17 mmol, 1.0 equiv) using general procedure 2. The final product was dried using MgSO_4_ and concentrated *in vacuo*. The crude was purified using Biotage Isolera One (1–15% MeOH in CH_2_Cl_2_) to afford **8d** (29 mg, 28% yield) as a white solid. *R*_f_ 0.2 (5% MeOH in CH_2_Cl_2_); mp 167–168 °C; FT-IR ν_max_/cm^–1^ (ATR) 3299, 1623, 1560, 1519, 1488, 1306, 1229, 1133, 975, 951, 844, 754, 692; ^1^H NMR (400 MHz, DMSO-*d*_6_) δ (ppm) 8.68 (q, *J* = 4.5 Hz, 1H), 8.36 (s, 1H), 8.27 (s, 1H), 7.60–7.71 (m, 2H), 7.38–7.47 (m, 2H), 7.10–7.22 (m, 5H), 4.89–4.98 (m, 1H), 4.44–4.58 (m, 1H), 3.92–4.06 (m, 1H), 3.53–3.62 (m, 1H), 3.34 (s, 3H), 3.24–3.30 (m, 1H), 2.72 (d, *J* = 4.5 Hz, 3H), 2.27–2.39 (m, 1H), 2.13–2.23 (m, 1H), 1.91–2.00 (m, 1H), 1.66–1.81 (m, 1H); ^13^C NMR (101 MHz, DMSO-*d*_6_) δ (ppm) 166.3, 164.7, 159.1, 158.7, 157.6, 156.9, 156.8, 156.1, 154.4, 143.9, 130.6, 130.5, 128.3, 124.3, 119.4 (x2), 117.8, 97.9, 52.2, 50.3, 47.4, 39.2, 29.7, 26.5, 24.0; HRMS (ESI+) *m*/*z* calculated for C_29_H_29_N_9_O_4_S [M + H]^+^ is 600.2136, found: 600.2132.

##### (R)-1-(1-(2-(Methylsulfonyl)pyrimidin-4-yl)piperidin-3-yl)-3-(4-phenoxyphenyl)-1H-pyrazolo[3,4-*d*]pyrimidin-4-amine (**8e**)

The compound was synthesized by dissolving 4-chloro-2-(methylsulfonyl)pyrimidine (50 mg, 0.26 mmol, 1.0 equiv) in CHCl_3_ (0.1 M) under an inert atmosphere followed by the addition of IbNH **2** (100 mg, 0.26 mmol, 1.0 equiv) and Et_3_N (54 μL, 0.39 mmol, 1.5 equiv). The reaction mixture was left to stir at rt for 16 h. It was then diluted with EtOAc and the organic phase was washed with sat. aq. NaHCO_3_ (x2) followed by brine (x1). The organic layer was dried using MgSO_4_ and concentrated *in vacuo*. The crude was purified by flash column chromatography (40% acetone in CH_2_Cl_2_) to afford **8e** (105 mg, 74% yield) as a white solid. *R*_f_ 0.2 (20% acetone in CH_2_Cl_2_); mp 159–163 °C; FT-IR ν_max_/cm^–1^ (ATR) 2359, 1634, 1568, 1488, 1312, 1231, 1128, 752; ^1^H NMR (400 MHz, DMSO-*d*_6_) δ ppm 8.29–8.34 (m, 1H), 8.27 (s, 1H), 7.58–7.65 (m, 2H), 7.39–7.48 (m, 2H), 7.07–7.22 (m, 6H), 4.80–4.89 (m, 1H), 4.10–4.70 (very br. m, 2H), 3.56–3.82 (br. m, 1H), 3.34–3.41 (m, 1H), 3.25 (s, 3H), 2.28–2.41 (m, 1H), 2.13–2.23 (m, 1H), 1.89–2.06 (m, 1H), 1.63–1.78 (m, 1H); ^13^C NMR (101 MHz, DMSO-*d*_6_) δ ppm 165.6, 161.7, 158.7, 157.6, 156.8, 156.5, 156.2, 154.5, 143.8, 130.6, 130.5, 128.4, 124.3, 119.4 (x2), 106.4, 97.9, 52.1, 48.1 (br), 44.7 (br), 39.1, 29.8, 23.6; HRMS (ESI+) *m*/*z* calculated for C_27_H_26_N_8_O_3_S [M + H]^+^ is 543.1921, found: 543.1925.

##### 1-(1-(4-Methyl-2-(methylsulfonyl)pyrimidin-5-yl)piperidin-3-yl)-3-(4-phenoxyphenyl)-1H-pyrazolo[3,4-*d*]pyrimidin-4-amine (**9a**)

The compound was synthesized from **7e** (85 mg, 0.16 mmol, 1.0 equiv) using general procedure 2. The final product was dried using MgSO_4_ and concentrated *in vacuo*. The crude was purified using Biotage Isolera One (1–15% MeOH in CH_2_Cl_2_) to afford **9a** (30 mg, 21% yield) as a white solid. *R*_f_ 0.2 (5% MeOH in CH_2_Cl_2_); mp 98–99 °C; FT-IR ν_max_/cm^–1^ (ATR)) 1559, 1473, 1407, 1338, 1307, 1232, 1127, 944, 842, 796, 752, 691; ^1^H NMR (400 MHz, DMSO-*d*_6_) δ (ppm) 8.97 (s, 1H), 8.21 (br s, 1H), 8.02–8.15 (m, 2H), 7.37–7.49 (m, 2H), 7.15–7.23 (m, 1H), 7.05–7.13 (m, 4H), 4.76–4.88 (m, 1H), 3.35 (s, 3H), 3.19–3.28 (m, 1H), 3.08–3.18 (m, 1H), 2.99–3.06 (m, 1H), 2.60–2.72 (m, 1H), 2.47 (s, 3H), 2.10–2.24 (m, 1H), 2.03–2.12 (m, 1H), 1.78–1.88 (m, 1H), 1.58–1.73 (m, 1H); ^13^C NMR (101 MHz, DMSO-*d*_6_) δ (ppm) 162.3, 158.9, 157.5, 156.7, 153.6, 153.3 (2C), 150.8, 144.3, 140.2, 131.0, 130.6,128.5, 124.3, 119.5, 118.7, 100.7, 53.5, 50.0, 45.2, 40.0, 30.2, 25.1, 21.4; HRMS (ESI+) *m*/*z* calculated for C_28_H_28_N_8_O_3_S [M + H]^+^ is 557.2078, found: 557.2076.

##### (R)-1-(1-(2-(Methylsulfonyl)pyrimidin-5-yl)piperidin-3-yl)-3-(4-phenoxyphenyl)-1H-pyrazolo[3,4-*d*]pyrimidin-4-amine (**9b**)

The compound was synthesized by dissolving 5-fluoro-2-(methylsulfonyl)pyrimidine (46 mg, 0.26 mmol, 1.0 equiv) in CHCl_3_ (0.1 M) under an inert atmosphere followed by the addition of IbNH **2** (100 mg, 0.26 mmol, 1.0 equiv) and Et_3_N (54 μL, 0.40 mmol, 1.5 equiv). The solution was left to stir at rt for 16 h. The reaction mixture was diluted with EtOAc and the organic phase was washed with sat. aq. NaHCO_3_ (x2) followed by brine (x1). The organic layer was dried using MgSO_4_ and concentrated *in vacuo*. The crude was purified by flash column chromatography (10% acetone in CH_2_Cl_2_) to afford **9b** (24 mg, 17% yield) as a white solid. *R*_f_ 0.1 (30% acetone in CH_2_Cl_2_); mp 173–176 °C; FT-IR ν_max_/cm^–1^ (ATR) 1624, 1564, 1481, 1318, 1234, 1125; ^1^H NMR (400 MHz, DMSO-*d*_6_) δ ppm 8.66 (s, 2H), 8.29 (s, 1H), 7.60–7.67 (m, 2H), 7.41–7.48 (m, 2H), 7.10–7.23 (m, 5H), 4.84–4.92 (m, 1H), 4.26–4.32 (m, 1H), 4.05–4.15 (m, 1H), 3.57–3.65 (m, 1H), 3.15–3.24 (m, 1H), 3.27 (s, 3H), 2.26–2.39 (m, 1H). 2.11–2.18 (m, 1H), 1.91–1.99 (m, 1H), 1.79–1.87 (m, 1H); ^13^C NMR (101 MHz, DMSO-*d*_6_) δ ppm 158.7, 157.6, 156.7, 156.2, 154.5, 154.0, 144.7, 143.7, 142.5, 130.6, 130.5, 128.3, 124.3, 119.4 (x2), 97.9, 51.8, 50.6, 46.6, 40.4, 29.9, 23.3; HRMS (ESI+) *m*/*z* calculated for C_27_H_26_N_8_O_3_S [M + H]^+^ is 543.1882, found: 543.1918.

##### (*R*)-(3-(4-Amino-3-(4-phenoxyphenyl)-1H-pyrazolo[3,4-*d*]pyrimidin-1-yl)piperidin-1-yl)(phenyl)methanone (**10**)

IbNH **2** (50 mg, 0.13 mmol, 1.0 equiv) was dissolved in anhydrous CH_2_Cl_2_ (1.3 mL) followed by the addition of benzoic anhydride (32 mg, 0.14 mmol, 1.1 equiv) and Et_3_N (62.3 μL, 0.20 mmol, 1.5 equiv). The reaction was stirred for 16 h at rt before diluting with EtOAc and washing the organic layer with sat. aq. NaHCO_3_ (x1) and brine (x1). The organic layer was dried using MgSO_4_ and concentrated *in vacuo*. The crude was purified by flash column chromatography (30% acetone in CH_2_Cl_2_) to afford **10** (58 mg, 90% yield) as a white solid. *R*_f_ 0.3 (30% acetone in CH_2_Cl_2_); mp 138–139 °C; FT-IR ν_max_/cm^–1^ (ATR) 2923, 1562, 1487, 1426, 1230; ^1^H NMR (400 MHz, 120 °C, DMSO-*d*_6_) δ ppm 8.34 (s, 1H), 7.73–7.85 (m, 2H), 7.41–7.60 (m, 7H), 7.18–7.38 (m, 5H), 6.55 (br. s, 2H), 4.91–5.03 (m, 1H), 4.26–4.40 (m, 1H), 3.93–4.05 (m, 1H), 3.68–3.80 (m, 1H), 3.33–3.42 (m, 1H), 2.40–2.51 (m, 1H), 2.29–2.39 (m, 1H), 2.08–2.18 (m, 1H), 1.75–1.88 (m, 1H). ^13^C NMR showed two conformers. When resolvable and supported by 2D NMR, both signals are provided. ^13^C NMR (101 MHz, DMSO-*d*_6_) δ ppm 169.7, 158.7, 157.6, 156.8, 156.1, 154.4, 143.8, 141.1, 130.6, 130.5, 129.9, 128.8, 128.4, 127.1, 124.3, 119.5, 119.4, 97.9, 52.3, 51.0/46.0, 47.9/42.1, 30.1, 24.9/23.6. HRMS (ESI+) *m*/*z* calculated for C_29_H_27_N_6_O_2_ [M + H]^+^ is 491.2190, found: 491.2199

##### (R)-(3-(4-Amino-3-(4-phenoxyphenyl)-1H-pyrazolo[3,4-*d*]pyrimidin-1-yl)piperidin-1-yl)(pyrimidin-4-yl)methanone (**12a**)

The compound was synthesized using general procedure 1 from 4-carboxypyrimidine (40 mg, 0.30 mmol, 1.0 equiv). The organic layer was dried using MgSO_4_ and concentrated *in vacuo*. The crude was purified by flash column chromatography (30% acetone in CH_2_Cl_2_) to afford **12a** (102 mg, 71% yield) as a white solid. *R*_f_ 0.1 (30% acetone in CH_2_Cl_2_); mp 173–174 °C; FT-IR ν_max_/cm^–1^ (ATR) 1618, 1565, 1477, 1230, 1129; ^1^H NMR showed two conformers in a 1:1 ratio. When resolvable and supported by 2D NMR, both signals associated with a proton are reported. ^1^H NMR (400 MHz, DMSO-*d*_6_) δ ppm 9.28/9.11 (d, *J* = 1.5 Hz, 1H), 8.99/8.82 (d, *J* = 5.1 Hz, 1H), 8.29/8.13 (s, 1H), 7.72/7.45 (dd, *J* = 5.1, 1.5 Hz, 1H), 7.66–7.72/7.60–7.66 (m, 2H), 7.39–7.48 (m, 2H), 7.09–7.21 (m, 5H), 4.80–4.93 (m, 1H), 4.59/3.44 (dd, *J* = 12.5, 4.0/12.5, 10.6 Hz, 1H), 4.07–4.17/3.30–3.40 (m, 1H), 3.75–3.92 (m, 1H), 3.54–3.64/3.18–3.29 (m, 1H), 2.24–2.38 (m, 1H), 2.07–2.22 (m, 1H), 2.04–2.14/1.80–1.91 (m, 1H), 1.62–1.77 (m, 1H). ^13^C NMR showed two conformers. When resolvable and supported by 2D NMR, both signals are provided. ^13^C NMR (100 MHz, DMSO-*d*_6_) δ ppm 165.5, 161.5, 161.0, 159.2/158.9, 158.7/158.6, 158.5/158.1, 157.6, 156.8, 156.2/155.9, 154.5/154.4, 143.9/143.6, 130.6, 130.5, 128.4/128.3, 124.2, 120.2/120.1, 119.5, 119.4, 97.9/97.8, 52.7/52.3, 50.6/46.9, 45.8/42.2, 29.9/29.1, 24.8/23.4. HRMS (ESI+) *m*/*z* calculated for C_27_H_24_N_8_O_2_ [M + H]^+^ is 493.2095, found: 493.2104.

##### (R)-(3-(4-Amino-3-(4-phenoxyphenyl)-1H-pyrazolo[3,4-*d*]pyrimidin-1-yl)piperidin-1-yl)(pyrimidin-5-yl)methanone (**12b**)

The compound was synthesized from 5-carboxypyrimidine (40 mg, 0.30 mmol, 1.0 equiv) using general procedure 1. The organic layer was dried using MgSO_4_ and concentrated *in vacuo*. The crude was purified by flash column chromatography (2% MeOH in CH_2_Cl_2_) to afford **12b** (64 mg, 44% yield) as a white solid. *R*_f_ 0.5 (2% MeOH in CH_2_Cl_2_); mp 140–141 °C; FT-IR ν_max_/cm^–1^ (ATR) 2921, 1622, 1565, 1477, 1230, 1130; ^1^H NMR showed two conformers. When resolvable and supported by 2D NMR, both signals associated with a proton are reported. ^1^H NMR (400 MHz, DMSO-*d*_6_) δ ppm **9**.28/9.15 (br s, 1H), 8.93/8.78 (br s, 2H), 8.29/8.14 (br s, 1H), 7.59–7.77 (m, 2H), 7.37–7.49 (m, 2H), 7.05–7.26 (m, 5H), 4.84–5.00 (m, 1H), 4.48–4.69/3.40–3.52 (m, 1H), 4.07–4.24/3.27–3.36 (m, 1H), 3.71–3.90 (m, 1H), 3.51–3.66/3.24–3.35 (m, 1H), 2.23–2.35 (m, 1H), 2.15–2.23 (m, 1H), 2.04–2.15/1.70–1.82 (m, 1H), 1.70–1.81 (m, 1H); ^13^C NMR showed two conformers. When resolvable and supported by 2D NMR, both signals are provided. ^13^C NMR (101 MHz, DMSO-*d*_6_) δ ppm 165.1, 159.3/159.2, 158.7/158.6, 157.6, 156.8, 156.2/156.0, 155.5, 154.5, 143.9/143.6, 130.6 (x2), 130.4/130.3, 128.4, 124.2, 119.5, 119.4, 97.8, 52.6/52.1, 51.4/46.1, 47.7/42.3, 30.0/29.2, 24.6/23.2. HRMS (ESI+) *m*/*z* calculated for C_27_H_24_N_8_O_2_ [M + H]^+^ is 493.2095, found: 493.2104.

## Supporting Information Available

Representative HPLC traces, NMR and MS spectra, Table S1, Figures S1–S5 (PDF)
Tables S2A–C, S3A,B, and S4A–C (XLSX)
Molecular formula strings (CSV)

## Notes

**Material Transfer:** Milligram quantities of the small molecules described in this manuscript are available upon request to M.B. (m.baud@soton.ac.uk)

## Abbreviations Used

AHR: aryl hydrocarbon receptor
AHRR: aryl hydrocarbon receptor repressor
AUC: area under the curve
BCR: B-cell receptor
BLNK: B-cell linker
BTK: Bruton tyrosine kinase
CLL: chronic lymphocytic leukemia
DAG: diacylglycerol
DLBCL: diffuse large B-cell lymphoma
E: enzyme
EGFR: epidermal growth factor receptor
EWG/EDG: electron withdrawing/donating group
FDA: Food and Drug Administration
GADPH: glyceraldehyde 3-phosphate dehydrogenase
GSEA: gene set enrichment analysis
HEK: human embryonic kidney
I: inhibitor
IgM: immunoglobulin M
IP3: inositol triphosphate
MAPKs: mitogen-activated protein kinases
MS: mass spectrometry
NFAT: nuclear factor of activated T-cells
NF-kB: nuclear factor kappa-light-chain-enhancer of activated B cells
nM: nanomolar
NMR: nuclear magnetic resonance
PIP2: phosphatidylinositol bisphosphate
PKC: protein kinase C
POI: protein of interest
RNA: ribonucleic acid
SAR: structure–activity relationship
SD: standard deviation
sIgM: surface immunoglobulin M
S_*N*_Ar: nucleophilic aromatic substitution
SYK: spleen tyrosine kinase
TCIs: targeted covalent inhibitors
UPLC-MS: ultraperformance liquid chromatography–mass spectrometry
UV: ultraviolet
XRD: X-ray diffraction
2-SP: 2-sulfonylpyrimidine
