# Supplementary material for: Exploring 2-Sulfonylpyrimidine Warheads as Acrylamide Surrogates for Targeted Covalent Inhibition: A BTK Story
Source: J Med Chem. 2024 Aug 9;67(16):13572–93. doi: 10.1021/acs.jmedchem.3c01927 (PMC11345841; doi:10.1021/acs.jmedchem.3c01927)

Supporting Information

Exploring 2-Sulfonylpyrimidine Warheads as Acrylamide Surrogates for Targeted Covalent Inhibition: a BTK Story

Ruxandra Moraru[a], Beatriz Valle-Argos[b], Annabel Minton[b], Lara Buermann[b], Suyin Pan[a], Thomas E. Wales[c], Raji E. Joseph[d], Amy H. Andreotti[d], Jonathan C. Strefford[b], Graham Packham[b]*, Matthias G. J. Baud[a]*

[a]School of Chemistry and Institute for Life Sciences, University of Southampton, Southampton, SO17 1BJ, United Kingdom

[b]Cancer Sciences, Faculty of Medicine, University of Southampton, Southampton, SO16 6YD, United Kingdom.

[c]Department of Chemistry and Chemical Biology, Northeastern University, Boston, MA 02115, USA.

[d]Roy J. Carver Department of Biochemistry, Biophysics and Molecular Biology, Iowa State University, Ames, IA 50011, USA.

*To whom correspondence should be addressed:
m.baud@soton.ac.uk, G.K.Packham@soton.ac.uk

Table of Content

BTK Mass spectrometry experiments……………………………………………………………….3

Summary of the BCR pathway…………………………………………………………………6

Compound induced Ca^{2+} response……………………………………………………………...7

Western blot quantifications……………………………………………………………………7

Compound induced Ca^{2+} response (dose dependent)………………………………………...8

Cell viability analysed using annexin V/PI staining……………………………………………..9

Gene set enrichment analysis…………………………………………………………..…….10

Comparison of DTT and TCEP on the activity of BTK and its inhibition by compounds…....11

Inhibition of poly-Glu:Tyr *in vitro* phosphorylation by wild type BTK by 2-SP functionalised Ibrutinib analogues ……………………………………………………………………………12

Inhibition of poly-Glu:Tyr *in vitro* phosphorylation by BTK C481S by Ibrutinib and compounds **8a-d**……………………………………………………………………………………..…..…13

Characterisation of compounds **8a-d**…………………………………………………………14-21

Figure S1. Modification of intact FL WT BTK (top) by **8a (A)**, **8b (B)**, **8c (C)**, **8d (D)**, and Ibrutinib (**E**, control). All experiments show evidence of single modification, although at varying rates. The same experiments with FL C481S BTK mutant (bottom) show no sign of covalent modification. **(F)** Summary of calculated protein mass shifts for each covalent modifier.

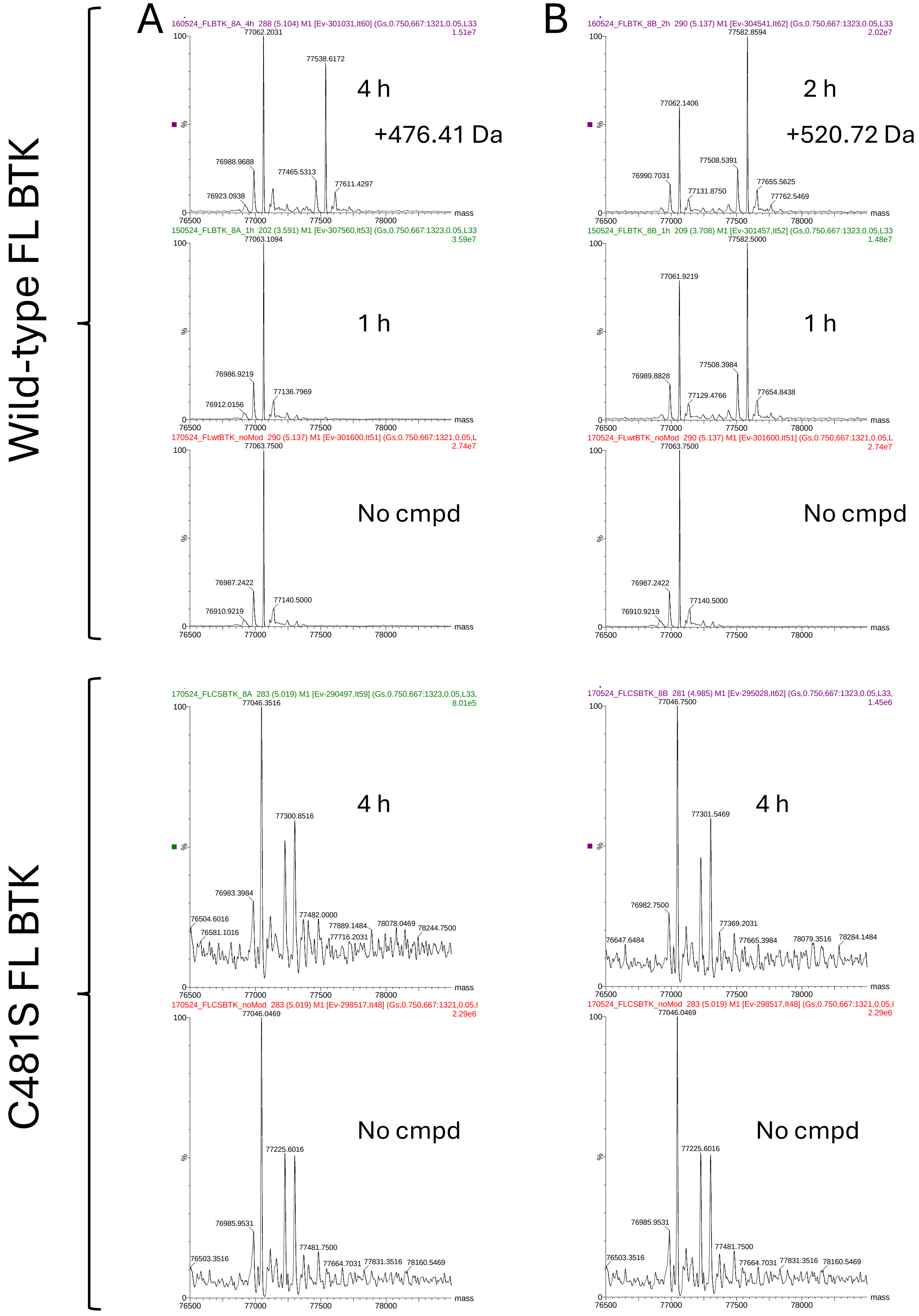

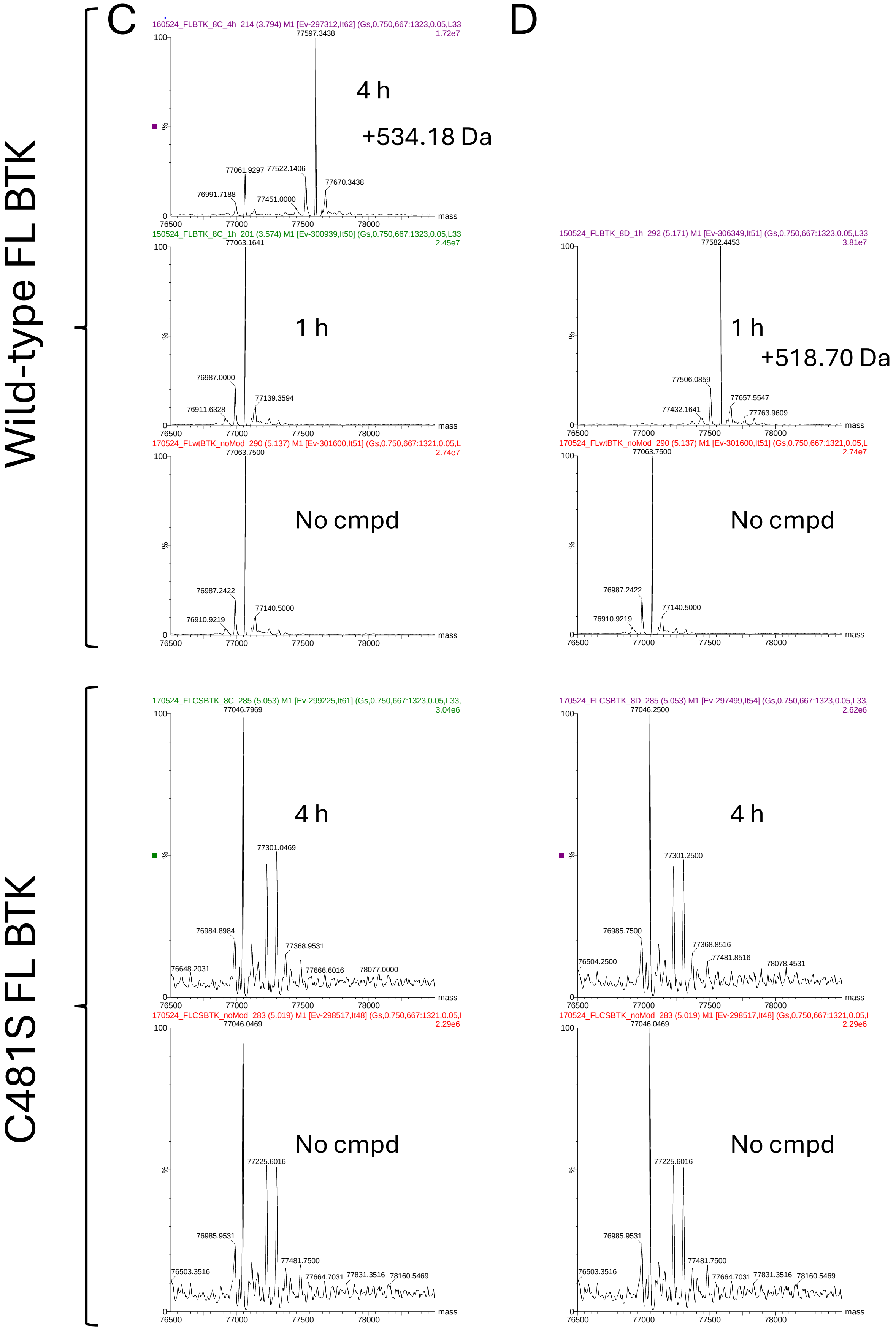

C
D
Wild-type FL BTK
C481S FL BTK
160524_FLBTK_8C_4h 214 (3.794) M1 [Ev-297312,It62] (Gs,0.750,667:1323,0.05,L33
1.72e7
77597.3438
4 h
+534.18 Da
77061.9297
77522.1406
76991.7188
77451.0000
77670.3438
150524_FLBTK_8C_1h 201 (3.574) M1 [Ev-300939,It50] (Gs,0.750,667:1323,0.05,L33
2.45e7
77063.1641
1 h
76987.0000
77139.3594
76911.6328
170524_FLwtBTK_noMod 290 (5.137) M1 [Ev-301600,It51] (Gs,0.750,667:1321,0.05,L
2.74e7
77063.7500
No cmpd
76987.2422
77140.5000
76910.9219
150524_FLBTK_8D_1h 292 (5.171) M1 [Ev-306349,It51] (Gs,0.750,667:1323,0.05,L33
3.81e7
77582.4453
1 h
+518.70 Da
77506.0859
77657.5547
77432.1641
77763.9609
170524_FLCSBTK_8C 285 (5.053) M1 [Ev-299225,It61] (Gs,0.750,667:1323,0.05,L33,
3.04e6
77046.7969
4 h
77301.0469
76984.8984
77368.9531
76648.2031
77666.6016
78077.0000
170524_FLCSBTK_noMod 283 (5.019) M1 [Ev-298517,It48] (Gs,0.750,667:1321,0.05,I
2.29e6
77046.0469
No cmpd
77225.6016
76985.9531
77481.7500
76503.3516
77664.7031
77831.3516
78160.5469
170524_FLCSBTK_8D 285 (5.053) M1 [Ev-297499,It54] (Gs,0.750,667:1323,0.05,L33,
2.62e6
77046.2500
4 h
77301.2500
76985.7500
77368.8516
76504.2500
77481.8516
78078.4531
100
%
0
76500
77000
77500
78000
mass

E

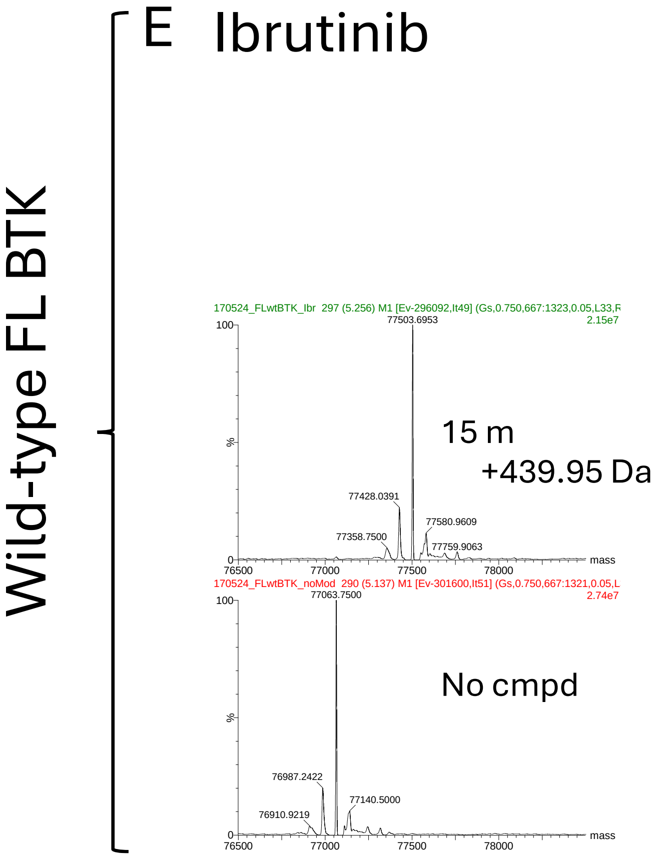

F

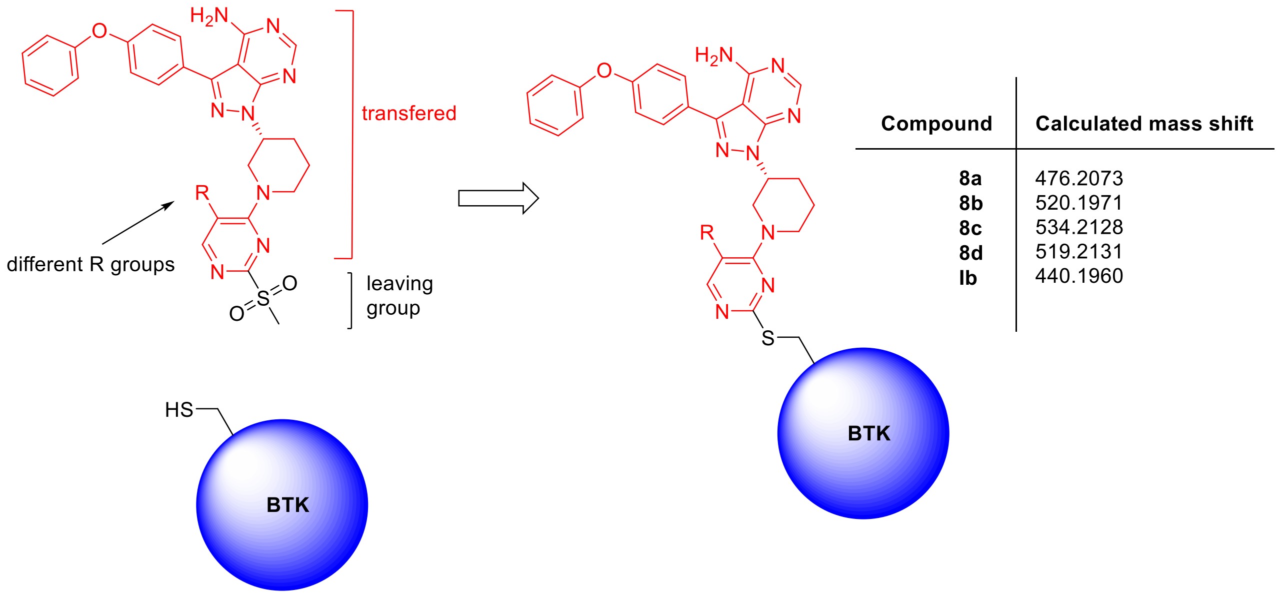

Compound	Calculated mass shift
**8a**	476.2073
**8b**	520.1971
**8c**	534.2128
**8d**	519.2131
**Ib**	440.1960

Figure S2. Activation of BTK and PLCγ2 downstream of the BCR. The BCR comprises an antigen-binding immunoglobulin coupled to CD79A and CD79B signal transduction molecules. BCR engagement leads to activation of proximal kinases, such as LYN and SYK. BTK is activated by SYK-mediated phosphorylation and autophosphorylation and, with the scaffold protein BLNK, mediates activation of PLCγ2. Once activated, PLCγ2 cleaves phosphatidylinositol 4,5-bisphosphate (PI(4,5)P2) to generate inositol 1,4,5-trisphosphate (IP3) and diacylglycerol (DAG), leading to increased intracellular Ca^{2+} (iCa^{2+}) and activation of PKC isoforms. Key downstream effects include activation of MAP kinases and Ca^{2+}-dependent transcription factors, such as nuclear factor-κB (NF-κB) and, *via* calmodulin (CaM) and calcineurin, nuclear factor of activated T-cells (NFAT), resulting in increased transcription of genes involved in control of survival, migration and proliferation. Note that not all pathways activated downstream of the BCR are shown.

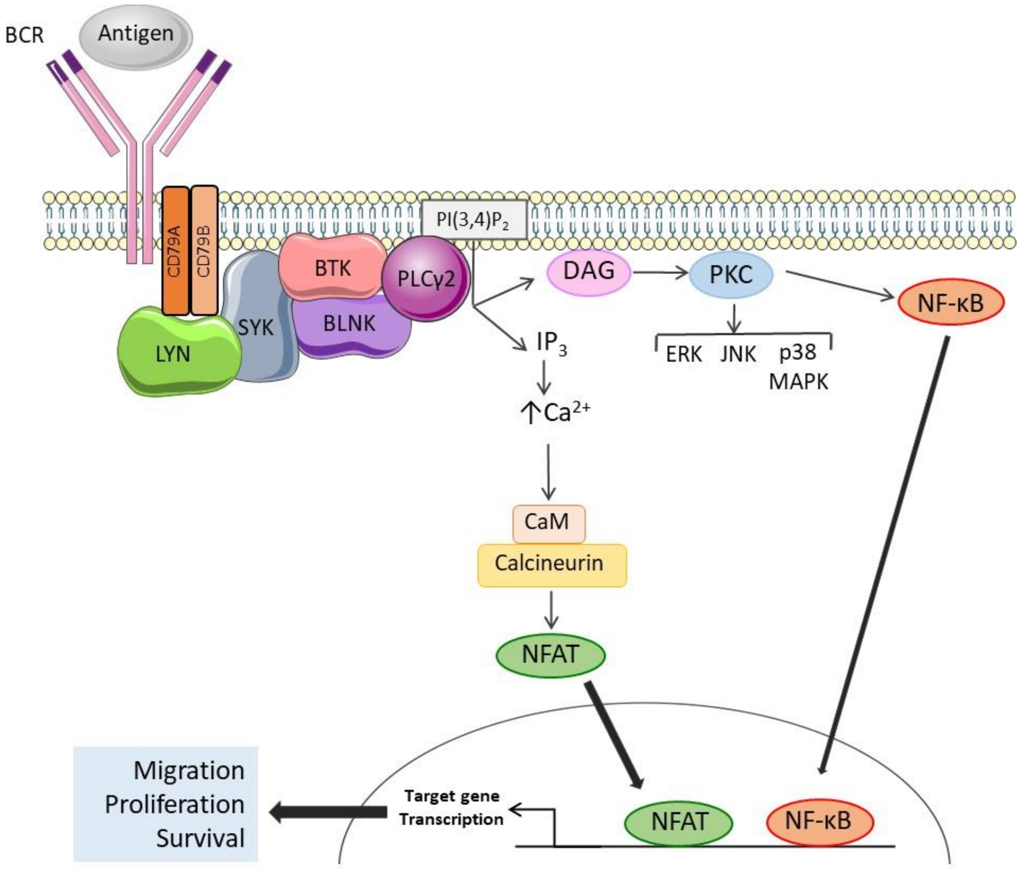

Figure S3. (A) Quantification of effects of compounds on anti-IgM-induced signalling in OCI-LY7 cells (relates to Figure 3 of the article main text). **Top:** Relative Ca^{2+} response (AUC; mean ±SD) with values for DMSO-treated cells set to 100%. Data are derived from 3-7 separate determinations. **Middle:** Relative phosphorylation for BTK Y^{223}, BTK Y^{551} and BLNK Y^{96} (mean ±SD derived from 3 independent experiments) **Bottom:** and PLCγ2 Y^{1197} and Y^{1217} (mean ±range derived from 2 independent experiments) with values for anti-IgM/DMSO—treated cells set to 1.0. Where shown, P-values give the significance of the effects of compounds compared to DMSO (one sample t-tests). **(B)** Effect of Ibrutinib (left), **8a** (middle) and **8d** (right) on anti-IgM-induced Ca^{2+} fluxes in OCI-LY7 cells. Cells were treated with indicated concentrations of the compounds or DMSO for 1 hour before analysis of anti-IgM-induced Ca^{2+} responses. Arrows show time of addition of anti-IgM and then ionomycin. X-axis: time in seconds.

(A)

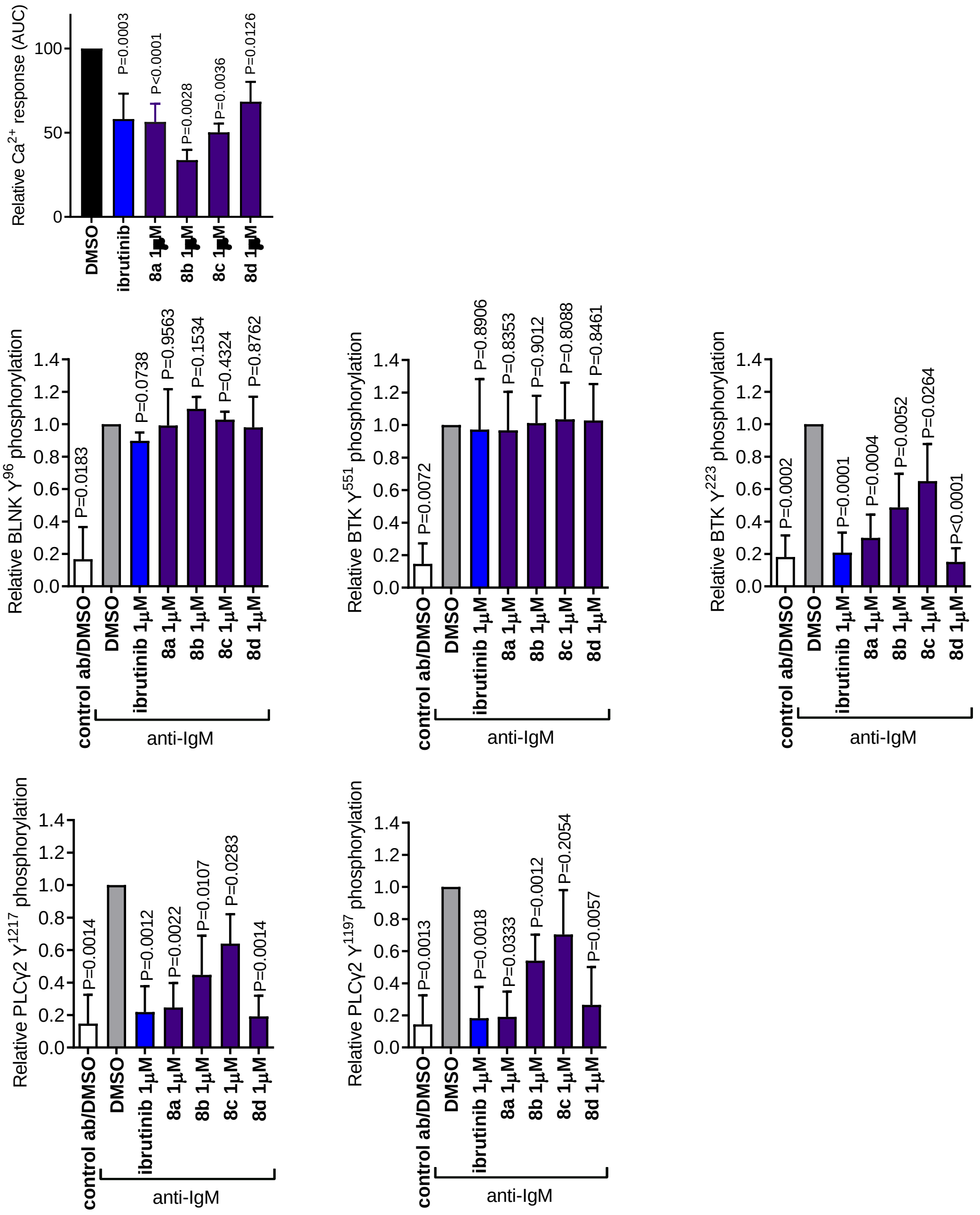

(B)

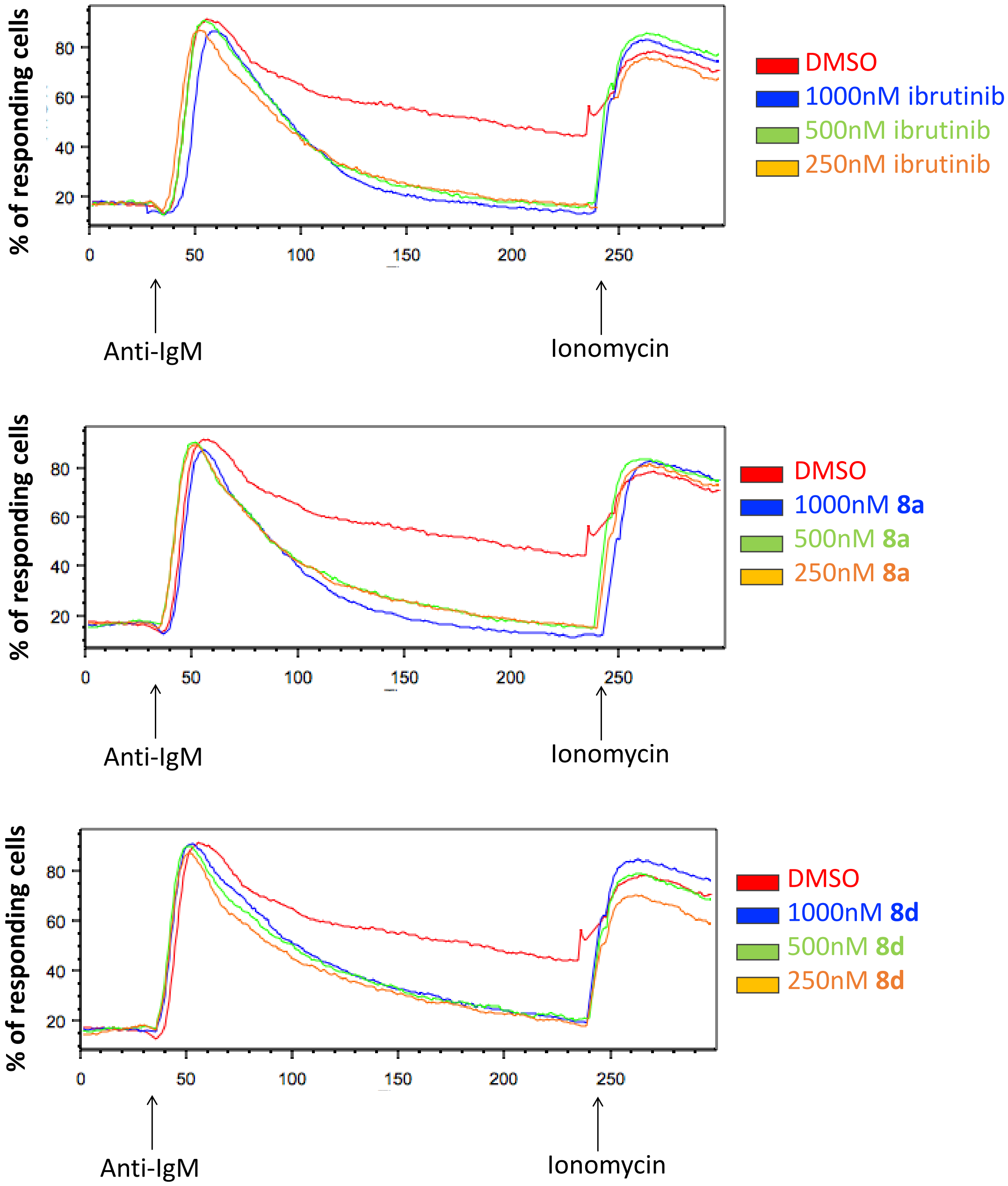
% of responding cells
80
60
40
20
0
50
100
150
200
250
Anti-IgM
Ionomycin
DMSO
1000nM ibrutinib
500nM ibrutinib
250nM ibrutinib
% of responding cells
80
60
40
20
0
50
100
150
200
250
Anti-IgM
Ionomycin
DMSO
1000nM **8a**
500nM **8a**
250nM **8a**
% of responding cells
80
60
40
20
0
50
100
150
200
250
Anti-IgM
Ionomycin
DMSO
1000nM **8d**
500nM **8d**
250nM **8d**

Figure S4. TMD8 cells were treated with the indicated concentrations of compounds for 72 hours before cell viability was analysed using annexin V/PI staining. Graph shows relative viability (i.e., annexin V^-/PI^- cells) with results for DMSO-treated cells set to 100% (derived from duplicate determinations).

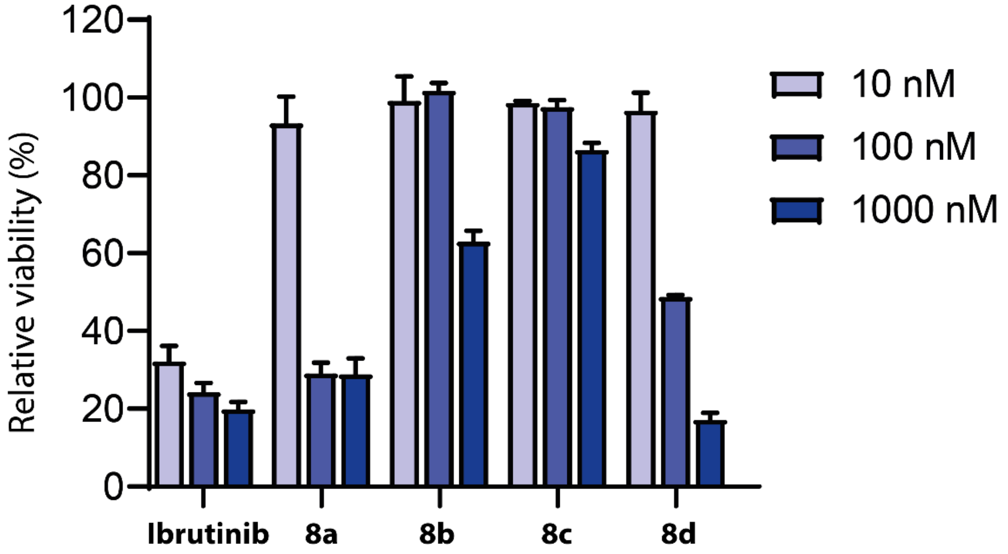

Figure S5. Gene set enrichment analysis. Figure shows results of comparisons between the gene expression signatures induce by the indicated compounds and the set of 125 genes that were downregulated by Ibrutinib (top row) and the set of 76 genes that were upregulated by Ibrutinib (bottom row).

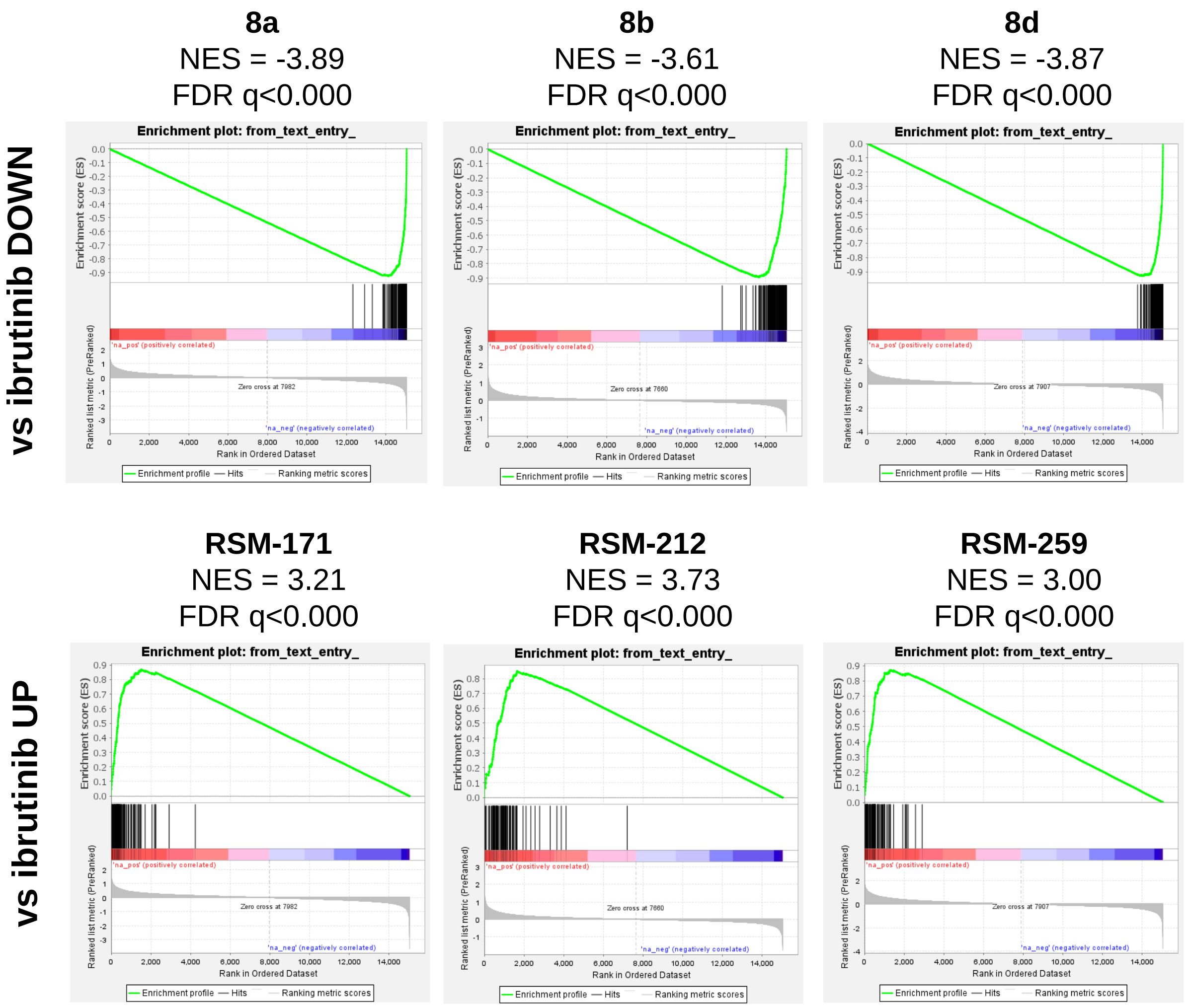

Figure S6. Left: activity of WT BTK in the presence of DTT (2 mM) or TCEP (1 mM) (mean±range of duplicate determinations). Right: effect of compounds on wild type BTK activity in the presence of TCEP or DTT. Compounds (100 nM) were pre-incubated with BTK in the presence of TCEP or DTT for 10 min before initiating the reaction by addition of ATP. Graphs show relative BTK kinase activity (mean±range from 2-4 determinations) relative to DMSO control (i.e. no compound, set to 100%).

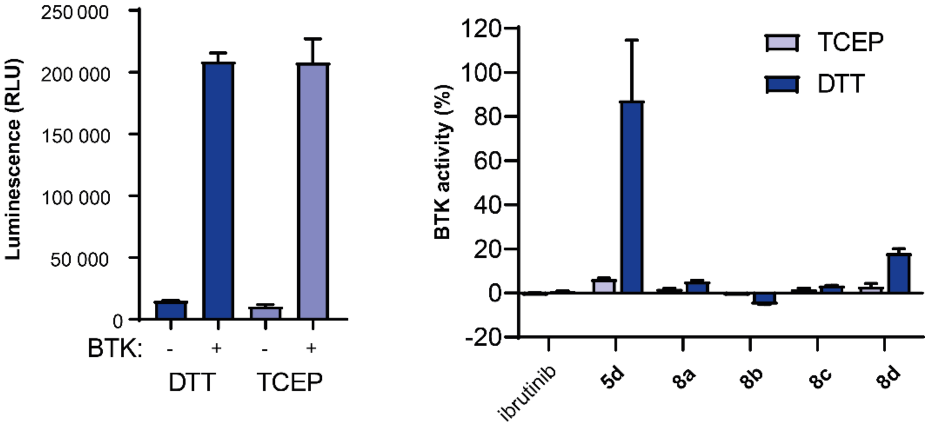

Table S1A. Inhibition of poly-Glu:Tyr *in vitro* phosphorylation by wild type BTK by 2-SP functionalised Ibrutinib analogues. Relative BTK activity (%, mean ± range, 2-12 replicates) in the presence of compounds (single concentration, 100 nM) normalised against DMSO control. The colour coding is the same as used in Figure 2A, blue = thioether intermediates, orange = sulfonyl derivatives.

	Compound	Structure	Remaining BTK activity (%)	Compound	Structure	Remaining BTK activity (%)
**Controls**	DMSO (control)		100 ± 2			
	Ibrutinib		2.1 ± 0.8			
**Non-covalent controls**	**10**	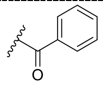	100 ± 0.2			
	**12b**	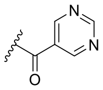	83 ± 7			
	**12a**	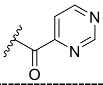	53 ± 2			
**5-amino**				**9b**	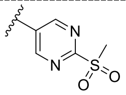	103 ± 5
	**7e**	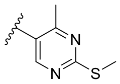	69 ± 2	**9a**	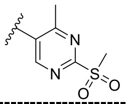	80 ± 3
**Amide linked**	**4b**	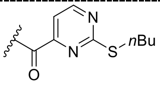	82 ± 1	**5b**	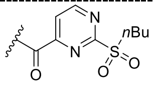	58 ± 3
	**4c**	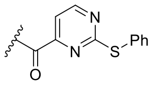	98 ± 5	**5c**	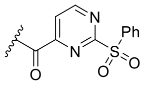	40 ± 1
	**4f**	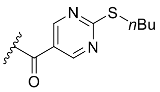	84 ± 5	**5f**	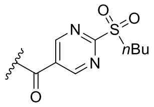	38 ± 2
	**4g**	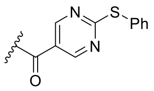	100 ± 6	**5g**	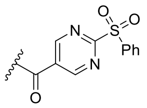	35 ± 1
	**4a**	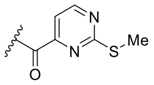	84 ± 8	**5a**	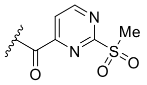	17 ± 1
	**4d**	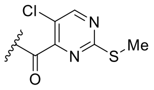	100 ± 6	**5d**	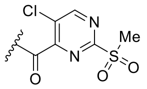	17 ± 2
	**4e**	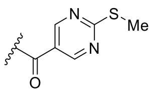	83 ± 9	**5e**	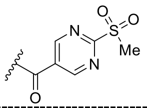	9 ± 2
**4-amino**				**8e**	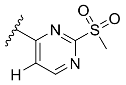	48 ± 0.3
	**7a**	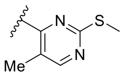	40 ± 0.3	**8a**	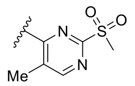	4 ± 1
	**7b**	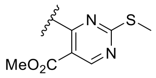	76 ± 2	**8b**	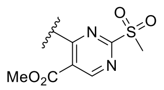	2 ± 1
			105 ± 4			
	**7c**	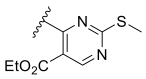		**8c**	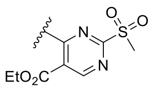	3 ± 0.4
	**7d**	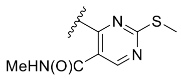	78 ± 2	**8d**	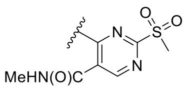	2.0 ± 0.5

Table S1B. Inhibition of poly-Glu:Tyr *in vitro* phosphorylation by BTK C481S by Ibrutinib and compounds **8a-d**. Relative BTK activity (%, mean ± range, 2 replicates) in the presence of compounds (100 nM) normalised against DMSO control.

Compound	Structure	Remaining BTK activity (%)
DMSO (control)		100 ± 6
Ibrutinib		50 ± 1
**8a**	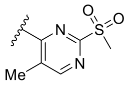	87 ± 11
**8b**	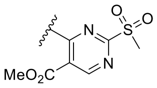	94 ± 1
**8c**	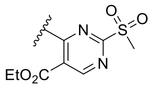	83 ± 0.1
**8d**	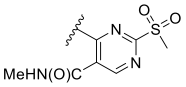	81 ± 4

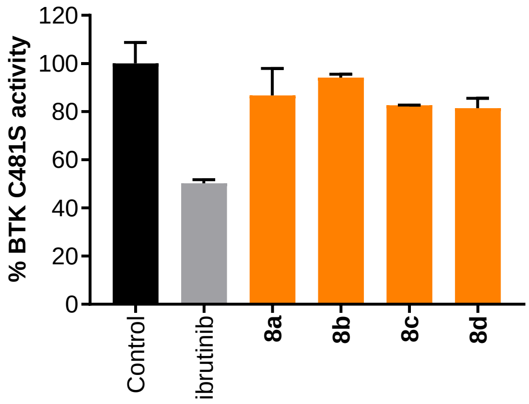

COPY OF REPRESENTATIVE SPECTRAL DATA

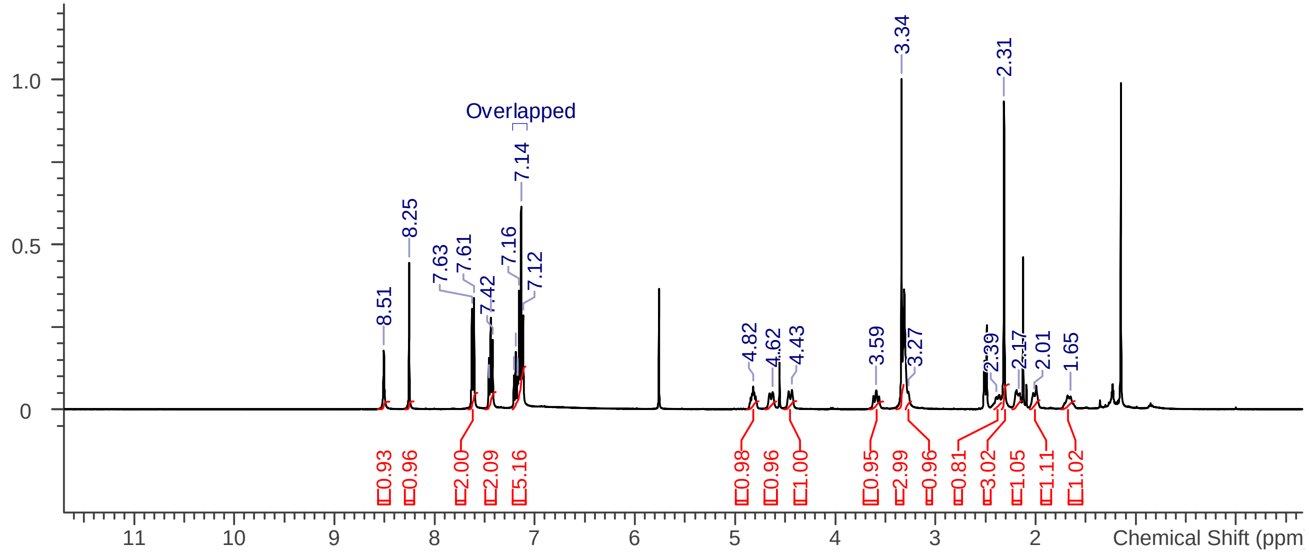

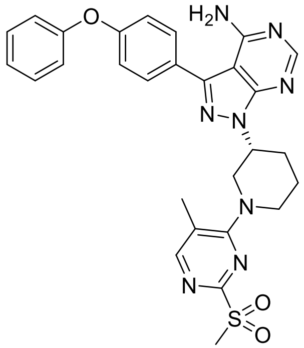

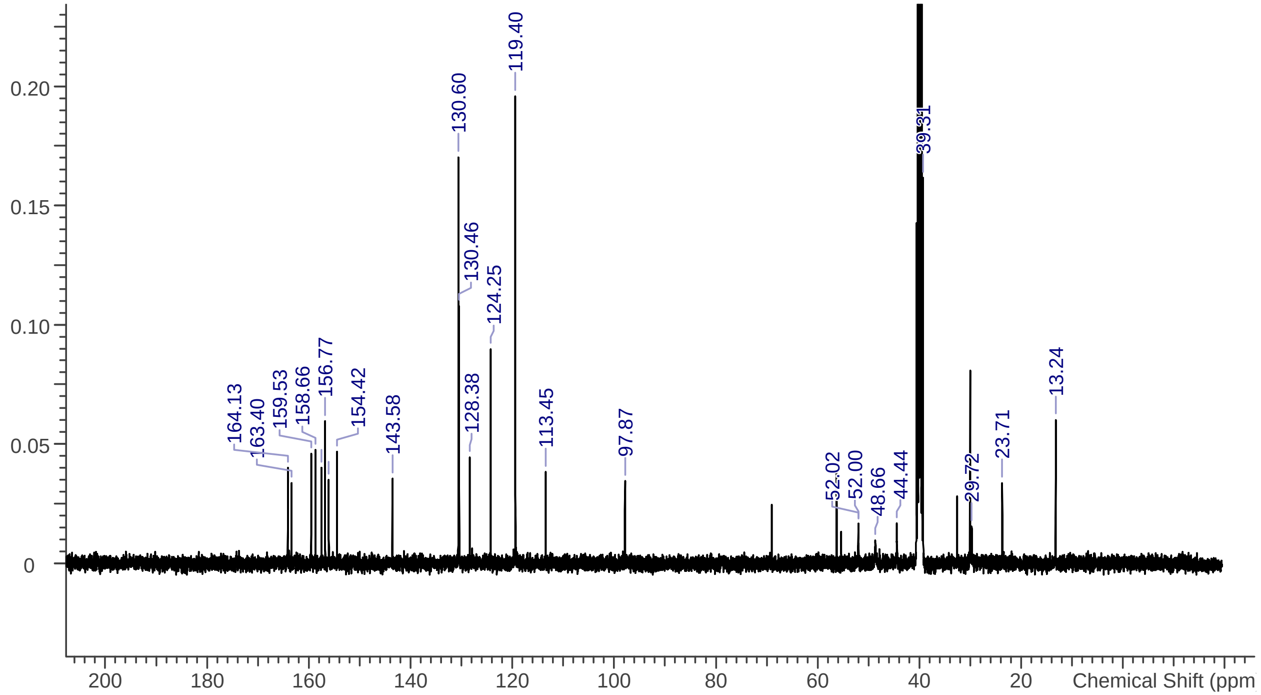

Chemistry - maXis HPLC-ESI Accurate Mass Report

Analysis Info

Analysis Name	D:\Data\Chemistry\2021\Nov\RSM 301_RA6_01_42966.d	Acquisition Date	26/11/2021 14:28:40
Method	soton lcms pos 120 to 1500.m	Operator	MSWEB@SOTON.AC.UK
Sample Name	RSM 301	Instrument / Ser#	maXis 17
Comment	Analyst: RB		

Acquisition Parameter

Source Type	ESI	Ion Polarity	Positive	Set Nebulizer	4.0 Bar
Focus	Not active	Set Capillary	4000 V	Set Dry Heater	230 °C
Scan Begin	120 m/z	Set End Plate Offset	-500 V	Set Dry Gas	6.0 l/min
Scan End	1500 m/z	Set Collision Cell RF	300.0 Vpp	Set Divert Valve	Waste

Cmpd 1, 2.6 min

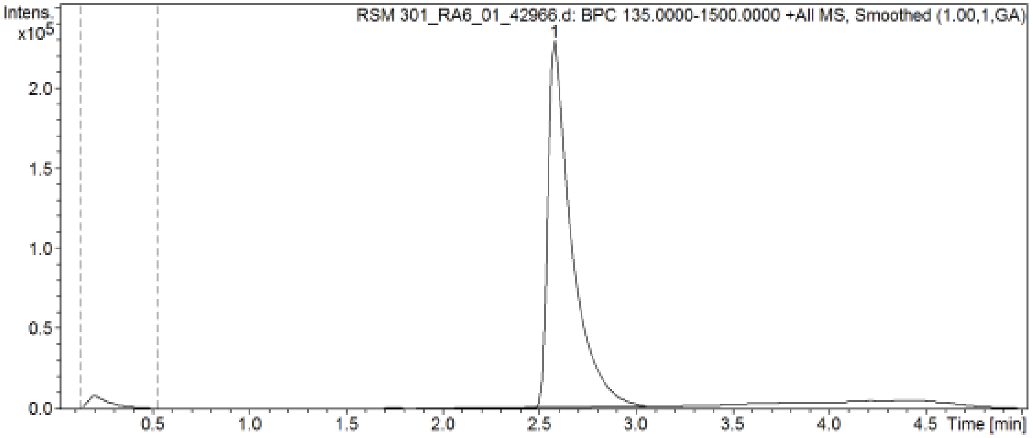

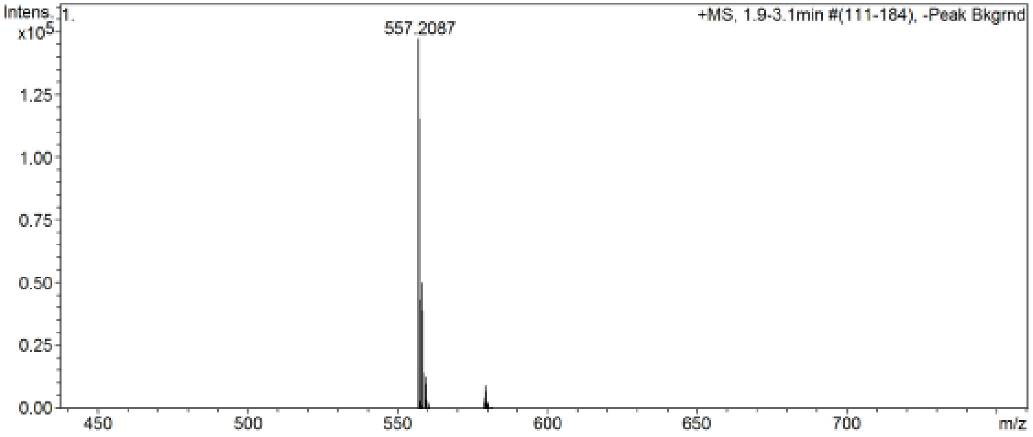

Absorbance, NL 1.546E06

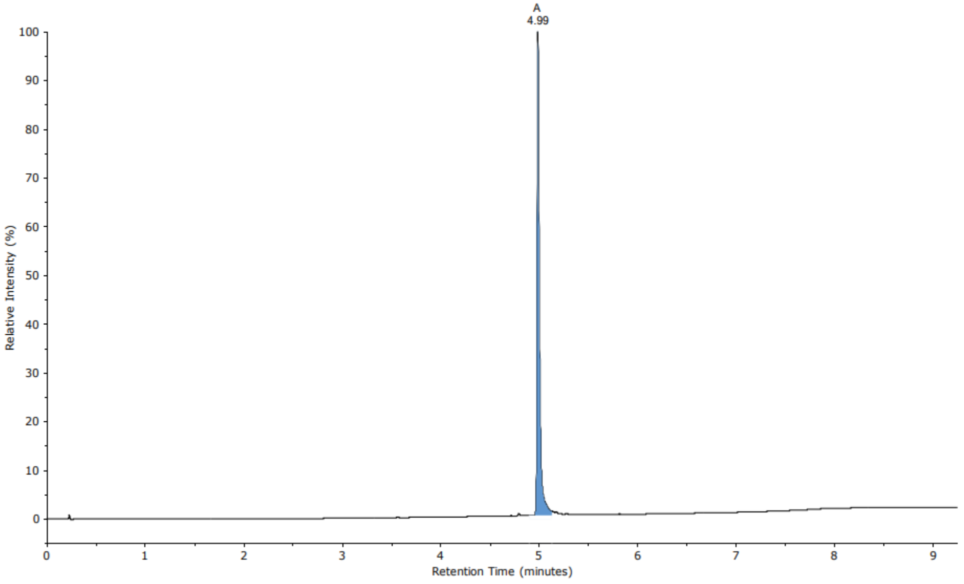

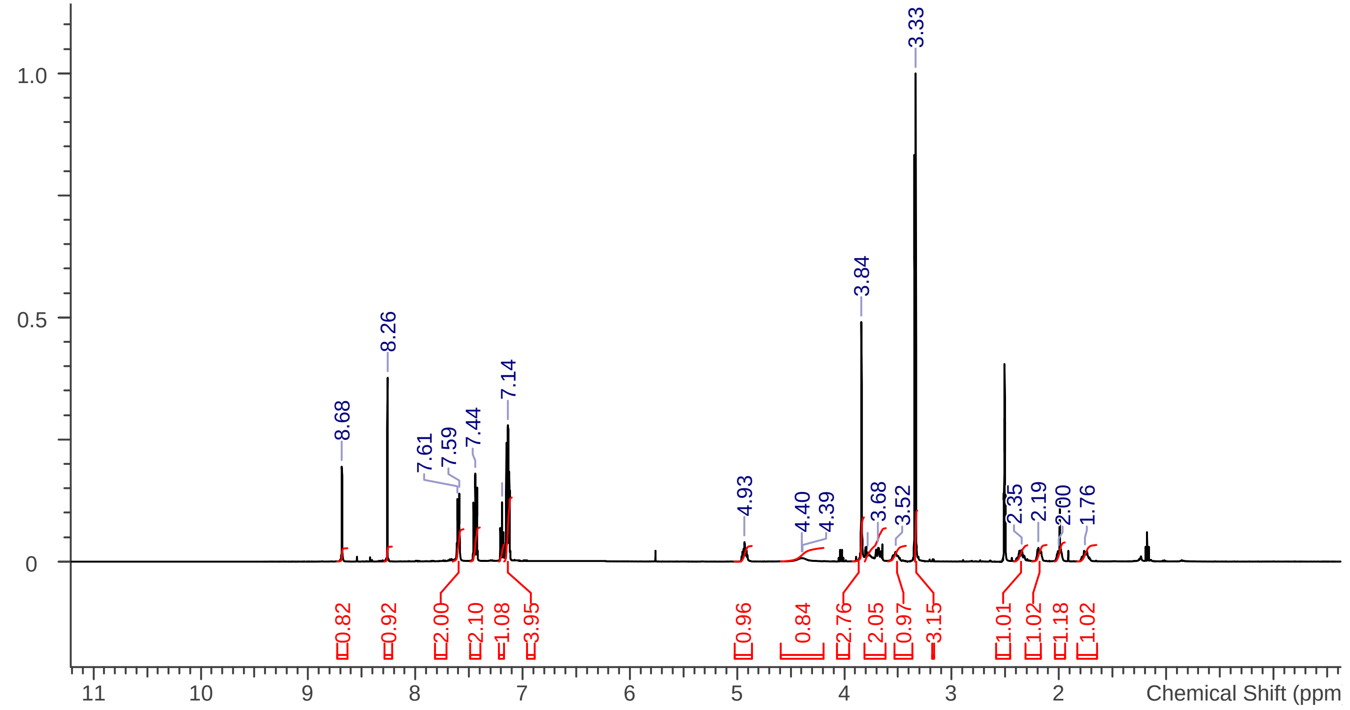
8.68
8.26
7.61
7.59
7.44
7.14
4.93
4.40
4.39
3.84
3.68
3.52
3.33
2.35
2.19
2.00
1.76
0.82
0.92
2.00
2.10
1.08
3.95
0.96
0.84
2.76
2.05
0.97
3.15
1.01
1.02
1.18
1.02
Chemical Shift (ppm

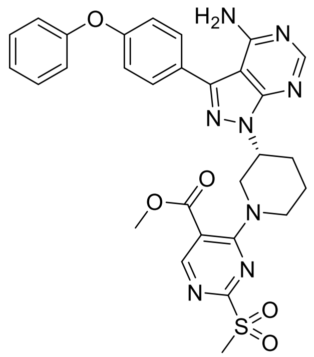
H2N
N
N
N
N
O
N
O
O
N
N
S
O
O

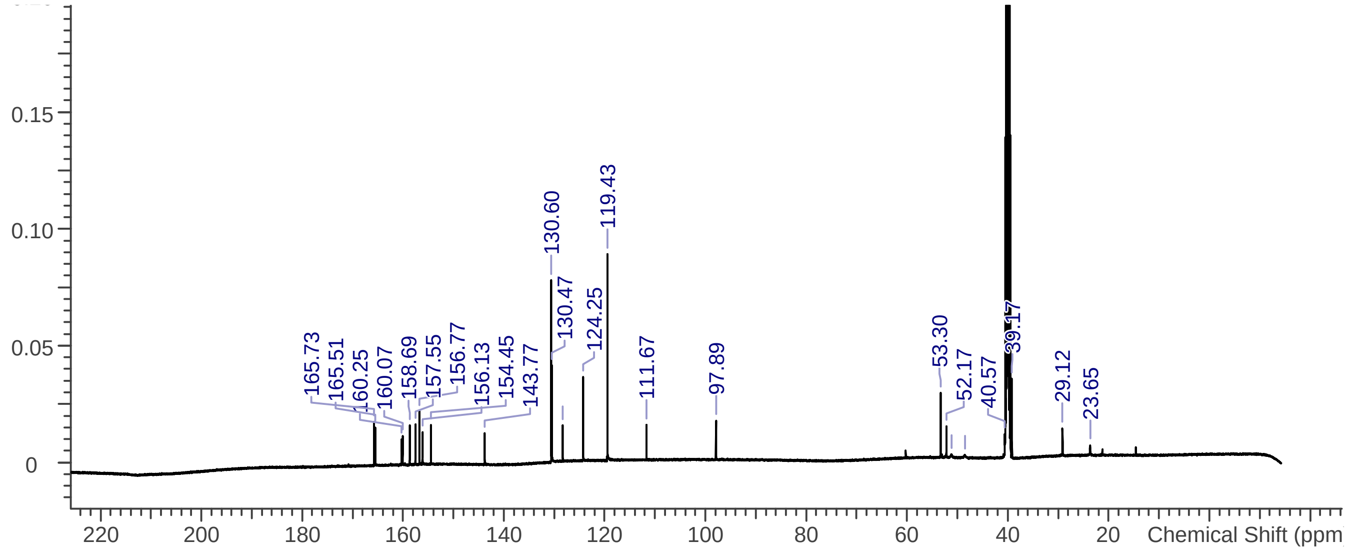
165.73
165.51
160.25
160.07
158.69
157.55
156.77
156.13
154.45
143.77
130.60
130.47
124.25
119.43
111.67
97.89
53.30
52.17
40.57
39.17
29.12
23.65
Chemical Shift (ppm

Chemistry - maXis HPLC-ESI Accurate Mass Report

Analysis Info

Analysis Name	D:\Data\Chemistry\2021\Nov\RSM 212_RA8_01_42968.d	Acquisition Date	26/11/2021 14:41:17
Method	soton lcms pos 120 to 1500.m	Operator	MSWEB@SOTON.AC.UK
Sample Name	RSM 212	Instrument / Ser#	maXis 17
Comment	Analyst: RB		

Acquisition Parameter

Source Type	ESI	Ion Polarity	Positive	Set Nebulizer	4.0 Bar
Focus	Not active	Set Capillary	4000 V	Set Dry Heater	230 °C
Scan Begin	120 m/z	Set End Plate Offset	-500 V	Set Dry Gas	6.0 l/min
Scan End	1500 m/z	Set Collision Cell RF	300.0 Vpp	Set Divert Valve	Waste

Cmpd 1, 2.4 min

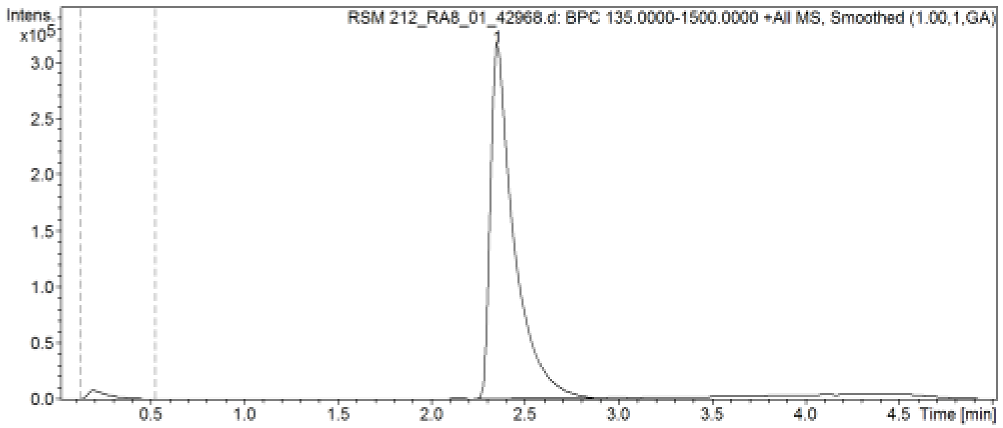

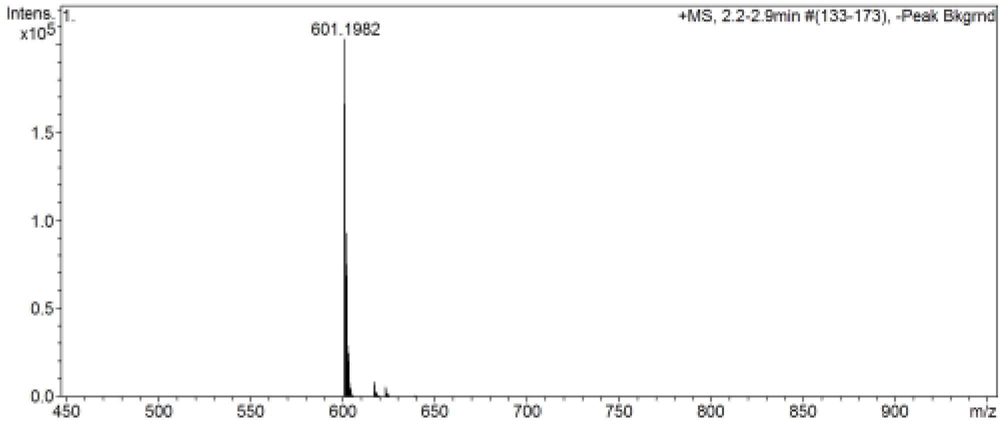

Absorbance, NL 1.771E06

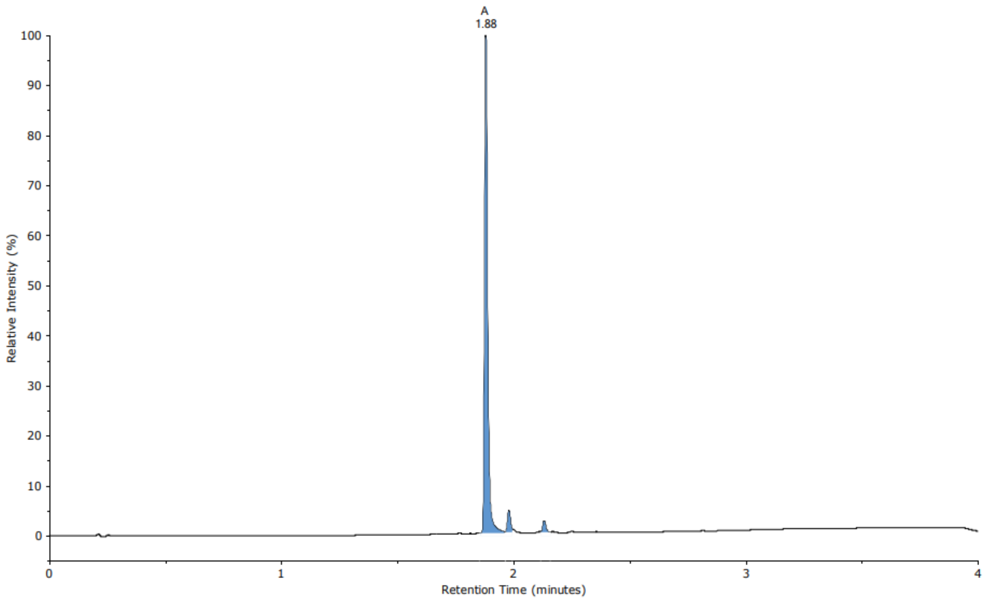

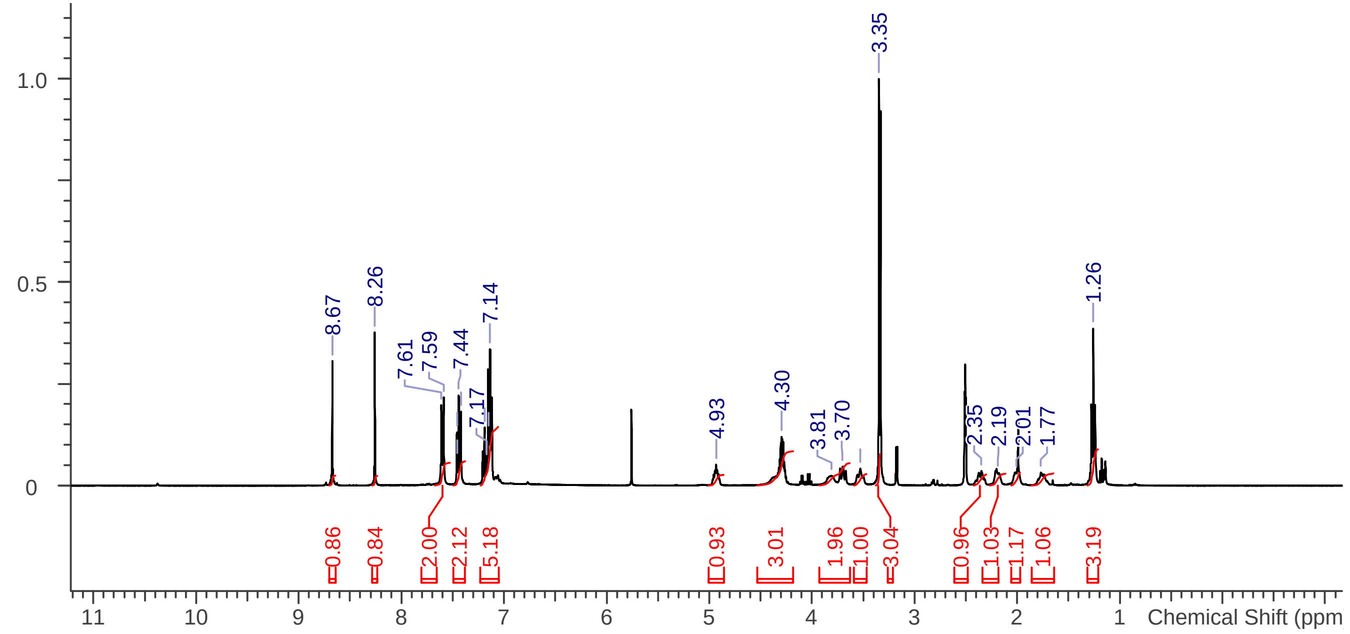

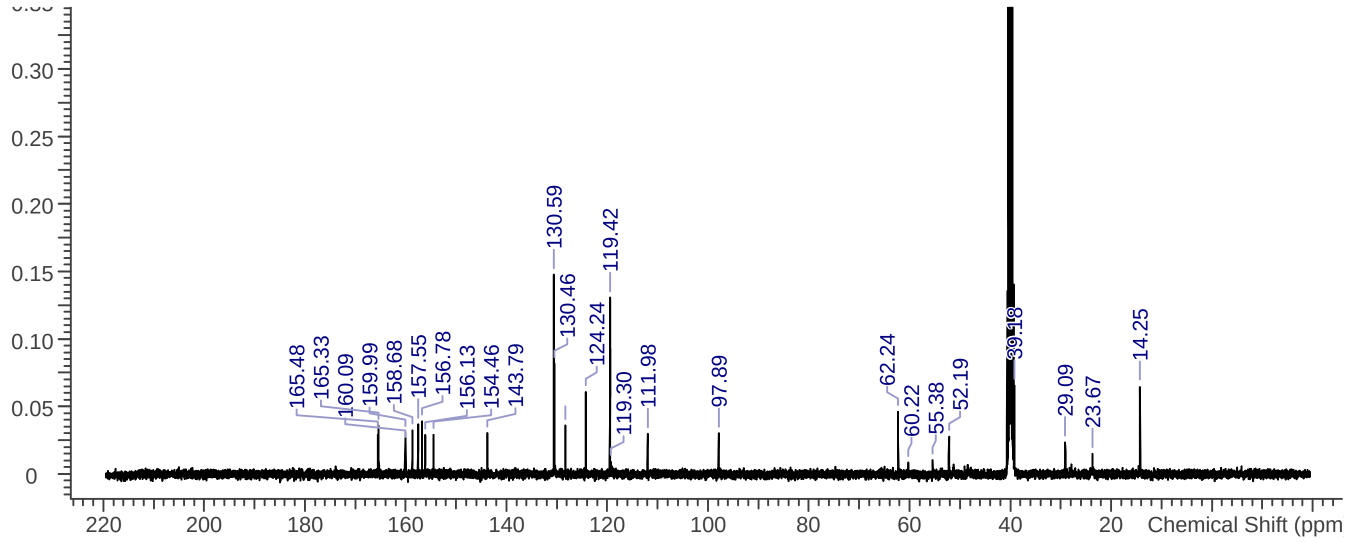

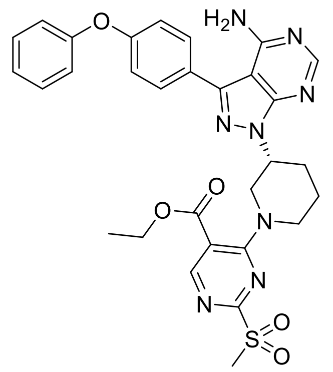

Acquisition Parameter					
Source Type	ESI	Ion Polarity	Positive	Set Nebulizer	4.0 Bar
Focus	Not active	Set Capillary	4500 V	Set Dry Heater	230 °C
Scan Begin	150 m/z	Set End Plate Offset	-500 V	Set Dry Gas	6.0 l/min
Scan End	1500 m/z	Set Collision Cell RF	300.0 Vpp	Set Divert Valve	Waste

Cmpd 1, 2.5 min

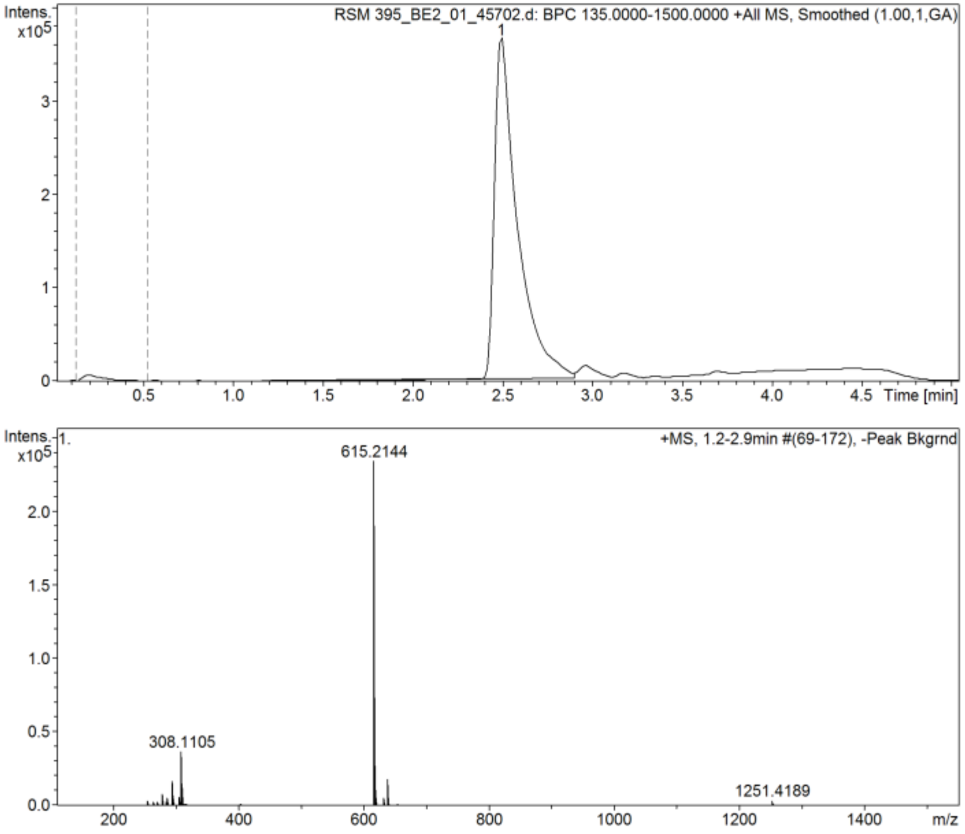

Absorbance, NL 2.728E05

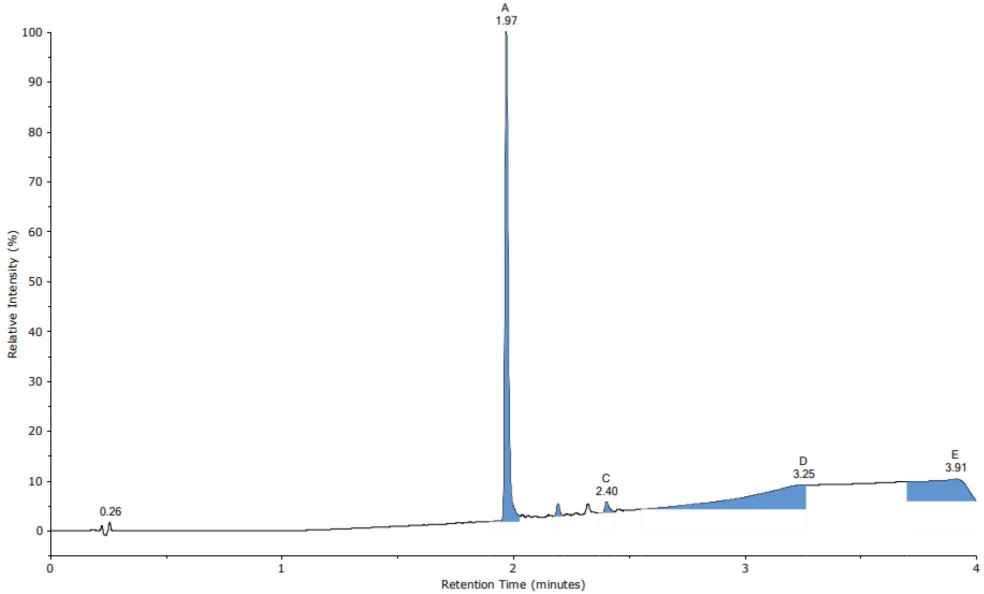

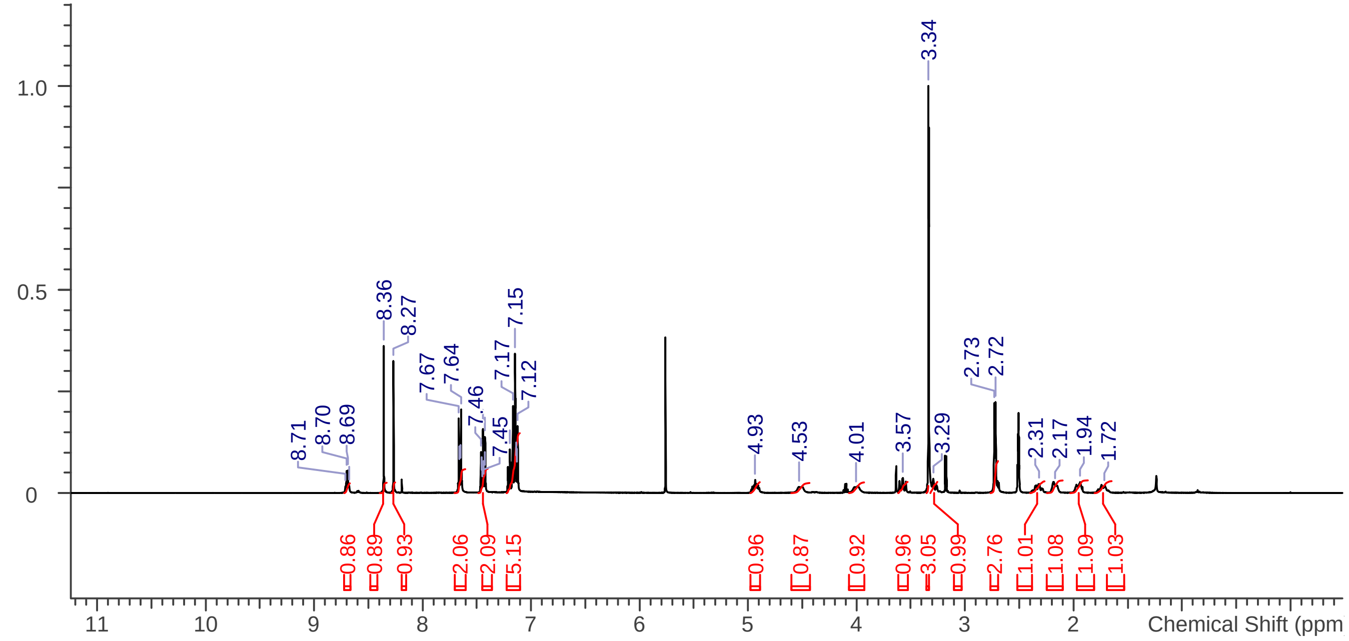
8.71
8.70
8.69
8.36
8.27
7.67
7.64
7.46
7.45
7.17
7.15
7.12
4.93
4.53
4.01
3.57
3.34
3.29
2.73
2.72
2.31
2.17
1.94
1.72
0.86
0.89
0.93
2.06
2.09
5.15
0.96
0.87
0.92
0.96
3.05
0.99
2.76
1.01
1.08
1.09
1.03
1.0
0.5
0
11
10
9
8
7
6
5
4
3
2
Chemical Shift (ppm

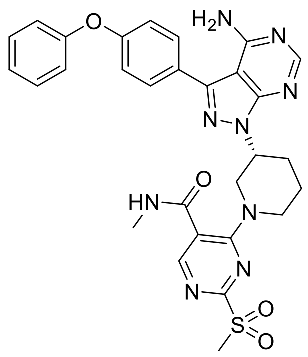
H2N
N
N
O
N
N
N
O
HN
N
N
N
S
O
O

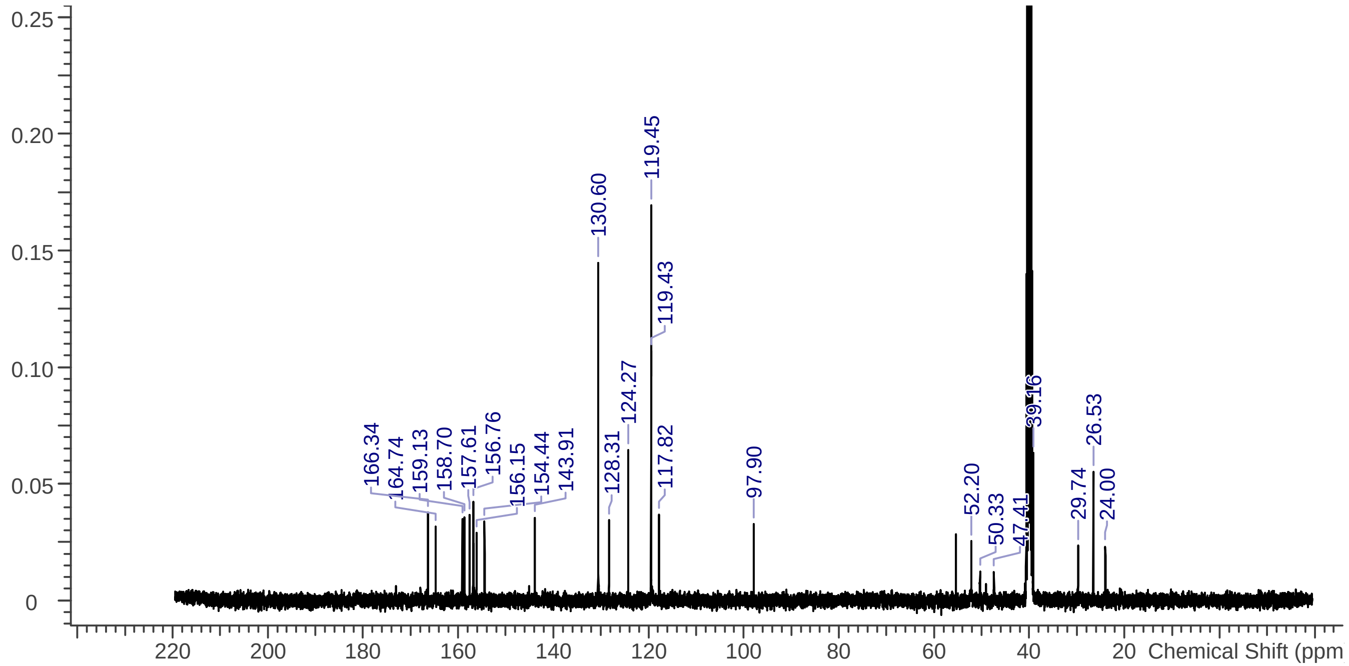
166.34
164.74
159.13
158.70
157.61
156.76
156.15
154.44
143.91
130.60
128.31
124.27
119.45
119.43
117.82
97.90
52.20
50.33
47.41
39.16
29.74
26.53
24.00
0.25
0.20
0.15
0.10
0.05
0
220
200
180
160
140
120
100
80
60
40
20
Chemical Shift (ppm

Chemistry - maXis HPLC-ESI Accurate Mass Report

Analysis Info

Analysis Name	D:\Data\Chemistry\2022\Nov\RSM 400_RA4_01_44731.d	Acquisition Date	17/11/2022 09:34:57
Method	soton lcms pos 120 to 1500.m	Operator	MSWEB@SOTON.AC.UK
Sample Name	RSM 400	Instrument / Ser#	maXis 17
Comment	Analyst: JMH		

Acquisition Parameter

Source Type	ESI	Ion Polarity	Positive	Set Nebulizer	4.0 Bar
Focus	Not active	Set Capillary	4500 V	Set Dry Heater	230 °C
Scan Begin	120 m/z	Set End Plate Offset	-500 V	Set Dry Gas	6.0 l/min
Scan End	1500 m/z	Set Collision Cell RF	300.0 Vpp	Set Divert Valve	Waste

Cmpd 1, 2.2 min

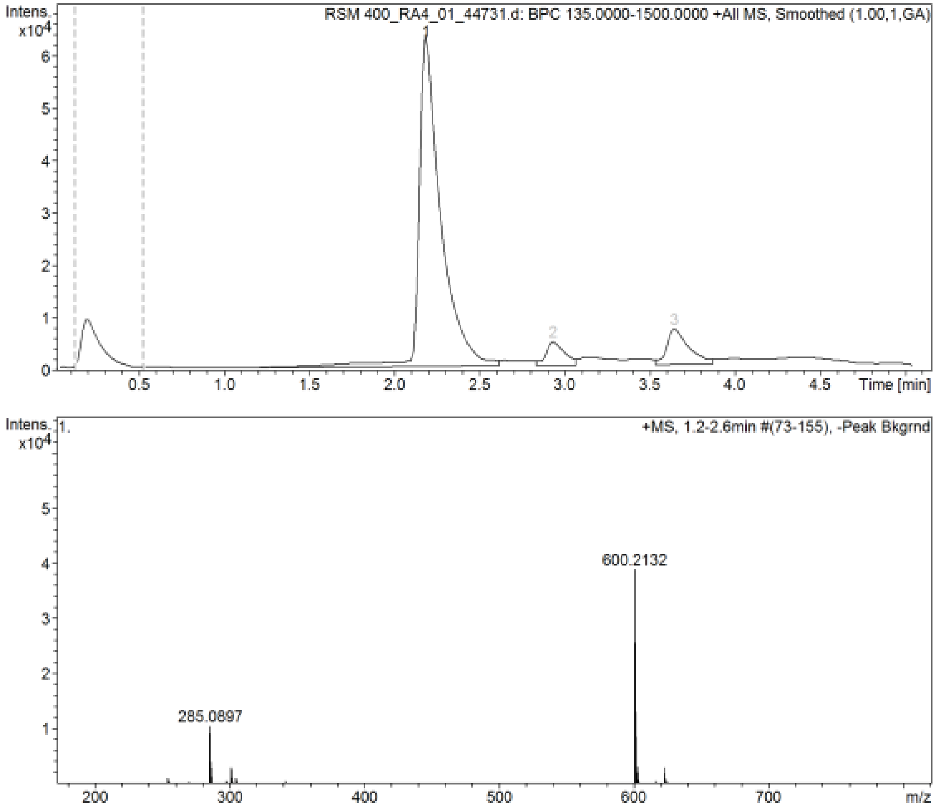

Absorbance, NL 3.031E06

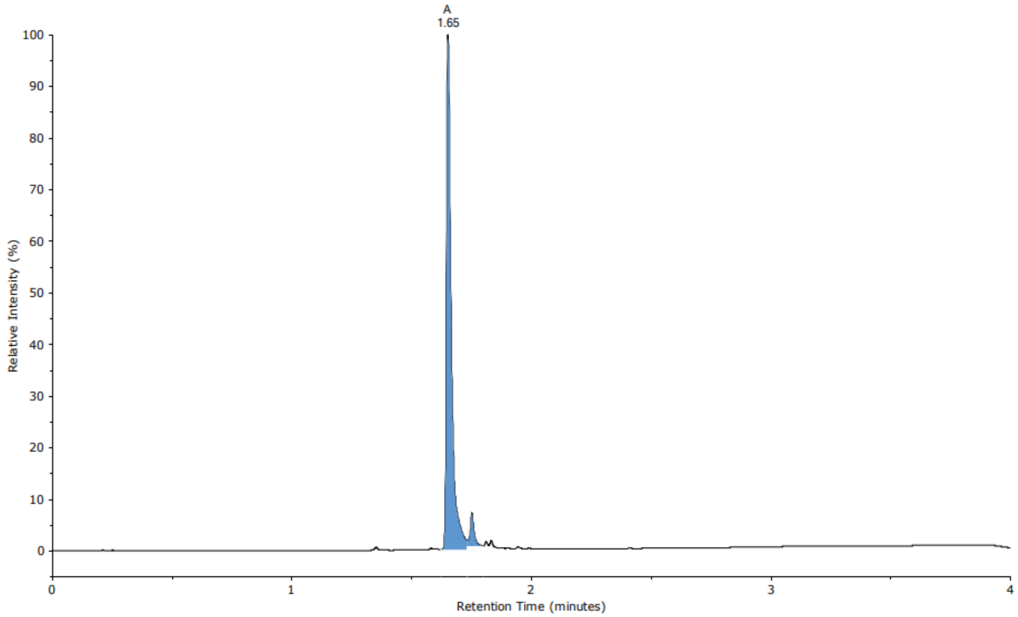

Supplement: Supplementary file 1 — jm3c01927_si_001.pdf [file jm3c01927_si_001.pdf]
